# Design, synthesis, *in vitro* and *in vivo* evaluation of benzylpiperidine-linked 1,3-dimethylbenzimidazolinones as cholinesterase inhibitors against Alzheimer’s disease

**DOI:** 10.1080/14756366.2019.1699553

**Published:** 2019-12-20

**Authors:** Jun Mo, Tingkai Chen, Hongyu Yang, Yan Guo, Qi Li, Yuting Qiao, Hongzhi Lin, Feng Feng, Wenyuan Liu, Yao Chen, Zongliang Liu, Haopeng Sun

**Affiliations:** a School of Pharmacy, China Pharmaceutical University, Nanjing, People’s Republic of China; b Department of Natural Medicinal Chemistry, China Pharmaceutical University, Nanjing, People’s Republic of China; c School of pharmacy, Yantai University, Yantai, People’s Republic of China; d Jiangsu Food and Pharmaceutical Science College, Huaian, People’s Republic of China; e School of Pharmacy, Nanjing University of Chinese Medicine, Nanjing, People’s Republic of China

**Keywords:** Alzheimer’s disease, cholinesterase inhibitor, 1,3-dimethylbenzimidazolinone derivatives, molecular docking, structural modification, structure-activity relationship

## Abstract

Cholinesterase inhibitor plays an important role in the treatment of patients with Alzheimer’s disease (AD). Herein, we report the medicinal chemistry efforts leading to a new series of 1,3-dimethylbenzimidazolinone derivatives. Among the synthesised compounds, **15b** and **15j** showed submicromolar IC_50_ values (**15b**, eeAChE IC_50_ = 0.39 ± 0.11 µM; **15j**, eqBChE IC_50_ = 0.16 ± 0.04 µM) towards acetylcholinesterase (AChE) and butyrylcholinesterase (BChE). Kinetic and molecular modelling studies revealed that **15b** and **15j** act in a competitive manner. **15b** and **15j** showed neuroprotective effect against H_2_O_2_-induced oxidative damage on PC12 cells. This effect was further supported by their antioxidant activity determined in a DPPH assay *in vitro*. Morris water maze test confirmed the memory amelioration effect of the two compounds in a scopolamine-induced mouse model. Moreover, the hepatotoxicity of **15b** and **15j** was lower than tacrine. In summary, these data suggest **15b** and **15j** are promising multifunctional agents against AD.

## 1. Introduction

Alzheimer’s disease (AD) is a chronic and progressive neurodegenerative disorder that is characterised by memory loss, disorientation, deficits in cognitive functions, and behavioural abnormalities^1–3^. Today, it is estimated that over 50 million people worldwide live with dementia, and this number is estimated to increase to more than 152 million by 2050. The current cost of the disease is about a trillion US dollars a year, and that is forecast to double by 2030^4^. Although the exact cause of AD is not yet understood, numerous literatures have suggested the involvement of several factors for the development of the disease, including low levels of acetylcholine (ACh) in the hippocampus and cortex area of the brain, the deposition of amyloid β (Aβ) peptide, the neurofibrillary tangles (p-Tau), and the oxidative stress, which is recently proved to play vital roles in the pathogenesis of AD^5–7^. Various drugs have been used over the past several years for the treatment of AD. Currently, there are only four drugs against AD in clinical, namely donepezil, rivastigmine, galantamine, and memantine^8^. Therefore, it is necessary to develop novel compounds with potential therapeutic value^9^^,^^10^.

Cholinergic system, which plays an important role in the regulation of learning, cognition, and memory processes^11^, has been extensively studied for the design of anti-AD drugs^12^^,^^13^. AChE contains two distinct binding sites: the catalytic active site (CAS), located at the bottom of the gorge, is the binding site for both substrates and inhibitors; the peripheral anionic site (PAS), situated at the entrance of the gorge, is the binding site of enzyme inhibitors; a mid-gorge recognition site between CAS and PAS is also identified^14^^,^^15^. BChE is expressed in neuroglia and is also found in the intestine, liver, kidney, heart, lung, and serum. It plays a major role in the metabolism of ester-containing compounds^16^. It can also hydrolyse ACh. In progressed AD patient, the level of AChE dramatically decreases due to the loss of neuron. However, it has been discovered that the level of BChE does not decline, or may even increase in progressed AD^17^. Therefore, BChE is a potential therapeutic target for restoring ACh levels in the brain, improving cognitive impairment, reducing adverse effects, especially in progressed AD patients. In summary, both acetylcholinesterase inhibitors (AChEIs) and butyrylcholinesterase inhibitors (BChEIs) are urgently needed for the treatment of AD^18–21^.

**Figure 1.**
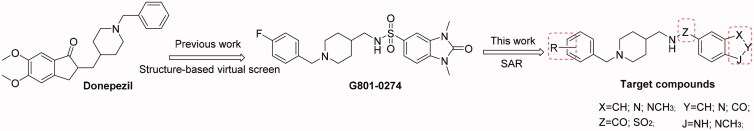
Design of a series of 1,3-dimethylbenzimidazolinone derivatives as cholinesterase inhibitors.

In our previous study, we discovered several new hits as ChEs inhibitors through pharmacophore-based virtual screening. **G801-0274** is one of the active hits^19^. With the aim to understand the structure-activity relationship (SAR), as well as to identify the pharmacophoric scaffold of this compound, structural modifications (Figure 1) and biological activity evaluations were performed and reported in this paper. Our data revealed a new type of ChEI with both *in vitro* and *in vivo* activity. It merits further medicinal chemistry efforts to identify therapeutic agents against AD.

## 2. Results and discussion

### 2.1. Chemistry

**Scheme 1.**
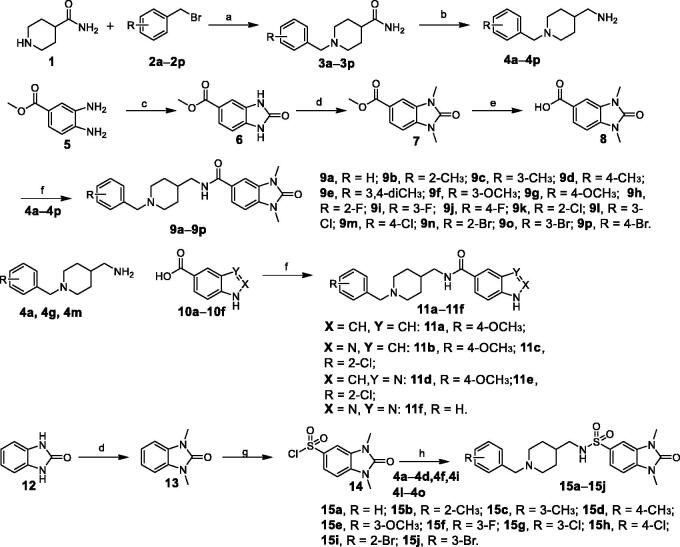
Reagents and conditions: (a) K_2_CO_3_, CH_3_OH, 85 °C, 5 h; (b) LiAlH_4_, THF, reflux, 5 h; (c) di(1*H*-imidazol-1-yl)methanone, CHCl_3_, 60 °C, 12 h; (d) NaH, MeI, DMF, r.t.; (e) THF, H_2_O, LiOH, 50 °C 2 h; (f) PyBOP, DIPEA, DMF, r.t, 4 h; (g) Chlorosulfonic acid, rt, 6 h; and (h) Et_3_N, CH_2_Cl_2_, r.t, 4 h.

The routes for the synthesis of the desired compounds **9a**–**9p**, **11a**–**11f**, **15a**–**15j** are illustrated in Scheme 1. Firstly, 4-piperidinecarboxamide (**1**) was treated with substituted benzyl bromides (**2a–2p**) using potassium carbonate in methanol to get 1–(1-substituted phenyl)piperidine-4-carbox-amides (**3a–3p**). **3a–3p** were treated with LiAlH_4_ in dry THF at 75 °C for 4 h to afford (1-benzylpiperidin-4-yl)methanamine derivatives (**4a–4p**). Secondly, compound **5** was treated with di(1*H*-imidazol-1-yl)methanone in chloroform at 60 °C for 12 h to obtain compound **6**, which was condensed with methyl iodide to yield compound **7**. Compound **7** was treated with lithium hydroxide to afford carboxylic acids **8**, which was condensed with (1-benzylpiperidin-4-yl)methanamine derivatives (**4a–4p**) to acquire target compounds **9a–9p**.

Compounds **10a–10f** were condensed with **4a, 4g,** and **4m** to yield target compounds **11a**–**11f**. Compound **12** was reacted with methyl iodide to yield compound **13**, which was then transformed to **14** using chlorosulfonic acid. The target compounds **15a**–**15j** were successfully obtained through condensation of **14** and different piperidine including **4a–4d, 4f, 4i,** and **4l–4o**.

### 2.2. Pharmacology

#### 2.2.1. AChE and BChE inhibitory activity of the target molecules

The synthesised compounds **9a–9p**, **11a–11f,** and **15a**–**15j** were assessed to determine their anti-cholinesterase activity. AChE and BChE inhibition activities were evaluated by the method described by Ellman^20^, wherein donepezil and tacrine were used as the reference compounds. The data were expressed as IC_50_ values (Table 1). *In vitro* assays proved that most of the compounds effectively inhibited ChEs in the micromolar range. Firstly, we explored the influence of sulphonamide group on the ChEs inhibitory activity by replacing it using amide group. We synthesised compounds **9a–9p**. Compared to **9a**, methyl substitution (**9b–9d**) led to a decrease of AChE inhibitory activity. The position of the methyl group was also considered, showing that the activity was meta- > ortho- > para-. For the multi-substituted compound, the 3,4-diCH_3_ (**9e**) showed reduced inhibitory activity. Next, we explored the impact of the methoxy group on ChEs inhibitory activities. The activity on AChE was para- > meta-. Compared to **9a**, **9f** and **9g** also showed reduced inhibitory activity.

**Table 1.** Structures, eeAChE and eqBChE inhibitory activities of target compounds.


Compound	R	X	Y	AChE^a^ (IC50^c^, μM or IR^d^, %)	BChE^b^ (IC50, μM or IR, %)	SI^e^
**9a**	H	–	–	3.14 ± 1.12	43.89 %	–
**9b**	2-CH_3_	–	–	8.08 ± 2.66	41.36 %	–
**9c**	3-CH_3_	–	–	3.83 ± 2.04	44.03 %	–
**9d**	4-CH_3_	–	–	25.71 ± 12.31	37.80 %	–
**9e**	3,4-CH_3_	–	–	9.97 ± 2.52	30.80 %	–
**9f**	3-OCH_3_	–	–	33.47 %	25.02 %	–
**9g**	4-OCH_3_	–	–	5.17 ± 2.25	41.46 %	–
**9h**	2-F	–	–	16.19 ± 4.34	30.01 %	–
**9i**	3-F	–	–	2.93 ± 1.96	95.07 ± 59.26	0.24
**9j**	4-F	–	–	4.70 ± 2.01	3.71 ± 1.88	1.27
**9k**	2-Cl	–	–	3.92 ± 1.20	46.48 %	–
**9l**	3-Cl	–	–	6.00 ± 3.31	36.93 %	–
**9m**	4-Cl	–	–	0.21 ± 0.03	49.93 %	–
**9n**	2-Br	–	–	8.87 ± 4.56	43.48 %	–
**9o**	3-Br	–	–	3.69 ± 1.83	42.83 %	–
**9p**	4-Br	–	–	11.06 ± 6.42	38.48 %	–
**11a**	4-OCH_3_	CH	CH	38.08 ± 12.83	10.75 ± 5.55	3.54
**11b**	4-OCH_3_	N	CH	48.57 %	44.57 %	–
**11c**	2-Cl	N	CH	5.44 ± 2.22	21.29 ± 5.00	0.26
**11d**	4-OCH_3_	CH	N	18.81 %	16.51 %	–
**11e**	2-Cl	CH	N	7.48 ± 3.10	42.12 %	–
**11f**	H	N	N	0.46 ± 0.40	43.07 %	–
**15a**	H	–	–	0.38 ± 0.08	0.45 ± 0.02	0.84
**15b**	2-CH_3_	–	–	0.39 ± 0.11	0.66 ± 0.16	0.59
		–	–	1.49 ± 0.43^f^	1.33 ± 0.55^g^	1.12
**15c**	3-CH_3_	–	–	0.54 ± 0.14	0.47 ± 0.17	1.14
**15d**	4-CH_3_	–	–	24.77 ± 3.48	100.2 ± 22.09	0.24
**15e**	3-OCH_3_	–	–	9.35 ± 2.32	20.34 ± 3.08	0.45
**15f**	3-F	–	–	1.04 ± 0.48	1.37 ± 0.74	0.75
**15g**	3-Cl	–	–	0.15 ± 0.02	1.01 ± 0.46	0.15
**15h**	4-Cl	–	–	1.95 ± 0.28	2.23 ± 0.81	0.87
**15i**	2-Br	–	–	0.42 ± 0.06	0.22 ± 0.04	1.99
**15j**	3-Br	–	–	0.39 ± 0.15	0.16 ± 0.04	2.44
		–	–	1.25 ± 0.48^f^	0.66 ± 0.22^g^	1.89
**Tacrine**		–	–	0.02 ± 0.01	0.008 ± 0.004	2.50
**Donepezil**		–	–	0.009 ± 0.001	1.57 ± 0.35	0.006

^a^ AChE (EC 3.1.1.7) from electric eel.

^b^ BChE (EC 3.1.1.8) from horse serum.

^c^ Concentration required for 50% inhibition of ChEs, data were shown in mean ± SEM of triplicate independent experiments.

^d^ Inhibitory rate of the compounds under 100 μM on ChEs.

^e^ Selectivity index (SI)=AChE IC_50_/BChE IC_50_.

^f^ AChE (EC 3.1.1.7) from human.

^g^ BChE (EC 3.1.1.8) from human.

Then, we evaluated the impact of halogen atoms on the ChEs activity. When substituted by F (**9h–9j**), Cl (**9k**–**9m**), Br (**9n**–**9p**) increased AChE inhibitory activity compared to compound **9a** (anti-AChE IC_50_ = 3.14 ± 1.12 µM). Especially, **9m** (anti-AChE IC_50_ = 0.21 ± 0.03 µM) increased 15-fold inhibitory activity towards AChE. When substituted by different halogen atoms, the activity on AChE was 4-Cl (**9m**) > 3-F (**9i**) > 3-Br (**9o**). And this series of structure were more selective towards AChE than BChE. However, they showed little inhibitory activity on BChE at 100 µM, except **9j** (anti-BChE IC_50_ = 3.71 ± 1.88 µM). After the sulphonamide group was replaced by amide group, the AChE inhibitory activity can be maintained at the micromolar level, but the BChE inhibitory activity was significantly decreased, indicating that sulphonamide group was very important for maintaining the BChE inhibitory activity, which may be related to the bond angle of the sulphonamide group to the molecule.

Next, we explored the effect of the benzimidazole on ChEs inhibitory activities. we synthesised compounds **11a–11f** with 1,3-dimethyl-1,3-dihydro-2*H*-benzo[*d*]imidazol-2-one replaced by indole, 1*H*-benzo[*d*]imidazole, 1*H*-indazole, or 1*H*-benzo[*d*][1,2,3]triazole. Simultaneously, we introduced methoxy and halogen atoms groups to the benzyloxyl moiety. We found that such structural modification resulted in a remarkably reduced activity on ChEs. We speculated that methyl groups occupy the active site pocket and interact with amino acid residues to increase inhibitory activity.

Finally, we synthesised compounds **15a–15j** with various kinds of groups introduced to the benzyl moiety, to explore the impact of the benzyl moiety structural modification on ChEs inhibitory activities. Compared to **9a**, **15a** showed an eightfold increase in inhibitory activity towards ChEs, indicating sulphonamide group can enhanced inhibitory activity. Firstly, we introduced methyl group to the benzyl moiety. Methyl substitution at ortho- or meta- position of benzyl (**15b** and **15c**, respectively) showed comparable activity to **15a** while the para-substituted compound **15d** led to reduced inhibitory activity. Next, we evaluated the impact of the methoxy group substituents on ChEs inhibitory activities. Compared to **15a**, **15e** showed reduced inhibitory activity. Then, compounds with different halogen substituents were also synthesised. When substituted by F (**15f**), it showed reduced inhibitory activity. When substituted by Cl (**15g–15h**), the activity on AChE was meta- > para-. When substituted by Br (**15i**–**15j**), the activity on AChE was meta- > para-. And **15j** with meta substitution were more selective having an enhanced inhibitory activity on BChE than AChE. When substituted by different halogen atoms, the activity on AChE was –Br > –Cl > –F. We speculated that Br and Cl can form halogen bonds with amino acid, but F cannot form halogen bond. Therefore, the compounds with Br and Cl substituents were more potent than F.

To further validated the inhibitory activities of synthesised compounds on human ChEs, the representative compounds, **15b** and **15j** were selected for determination (Table 1). **15b** exhibited huAChE IC_50_ = 1.49 ± 0.43 µM, huBChE IC_50_ = 1.33 ± 0.55 µM; **15j** exhibited huAChE IC_50_ = 1.25 ± 0.48 µM, huBChE IC_50_ = 0.66 ± 0.22 µM. The results showed that the synthesised compounds efficiently inhibited the activities of human ChEs, further confirrmed their activities as ChEs inhibitors.

#### 2.2.2. Kinetic studies of AChE and BChE inhibition

**Figure 2.**
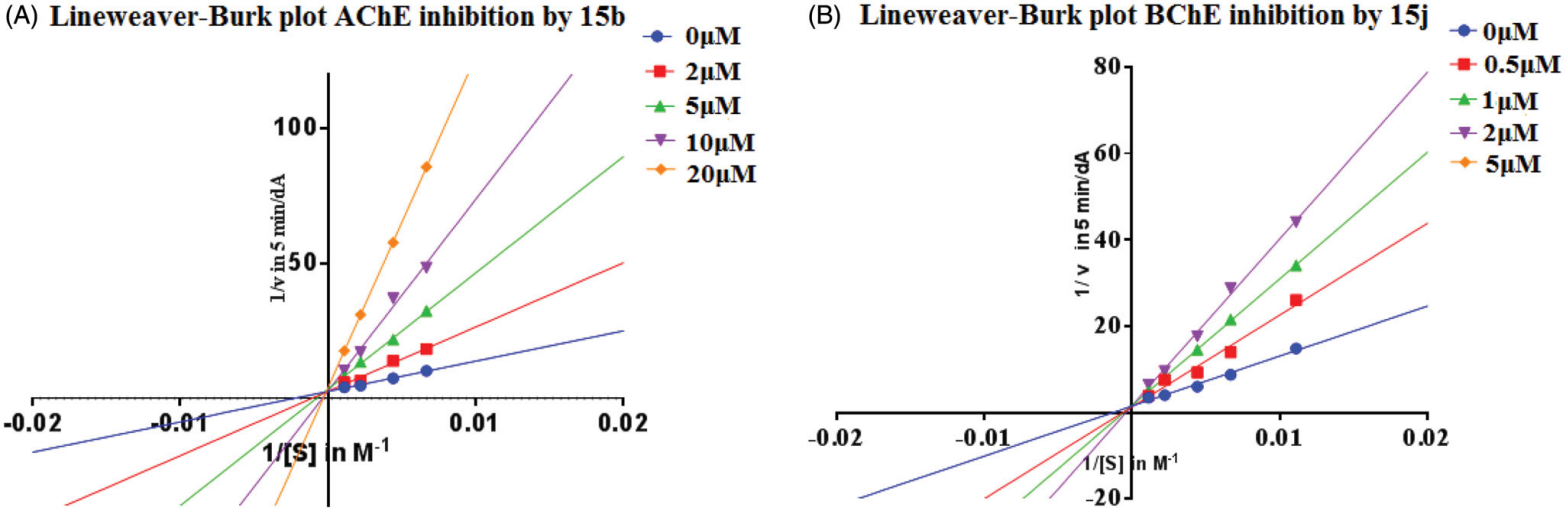
(A) Lineweaver–Burk plot for the inhibition of eeAChE by compound **15b** at different concentrations of substrate. (B) Lineweaver–Burk plot for the inhibition of eqBChE by compound **15j** at different concentrations of substrate.

In order to determine the kinetic type of AChE and BChE inhibition, compound **15b** and compound **15j** were selected for the kinetic study. In each case, the kinetic type of enzyme inhibition was obtained through the modified Ellman’s method and Lineweaver–Burk secondary plots^21^. As shown in Figure 2, the results showed that the plots of 1/V versus 1/[S] gave straight lines with different slopes depending on concentrations of the inhibitor, and the lines intersected on the vertical axis. The Lineweaver–Burk plot showed that Vmax is the same regardless of concentration of the inhibitor, and *K*_m_ increases with increasing concentration of the inhibitor. This behaviour indicates that **15b** and **15j** inhibit the ChEs in a competitive manner.

#### 2.2.3. Docking studies

**Figure 3.**
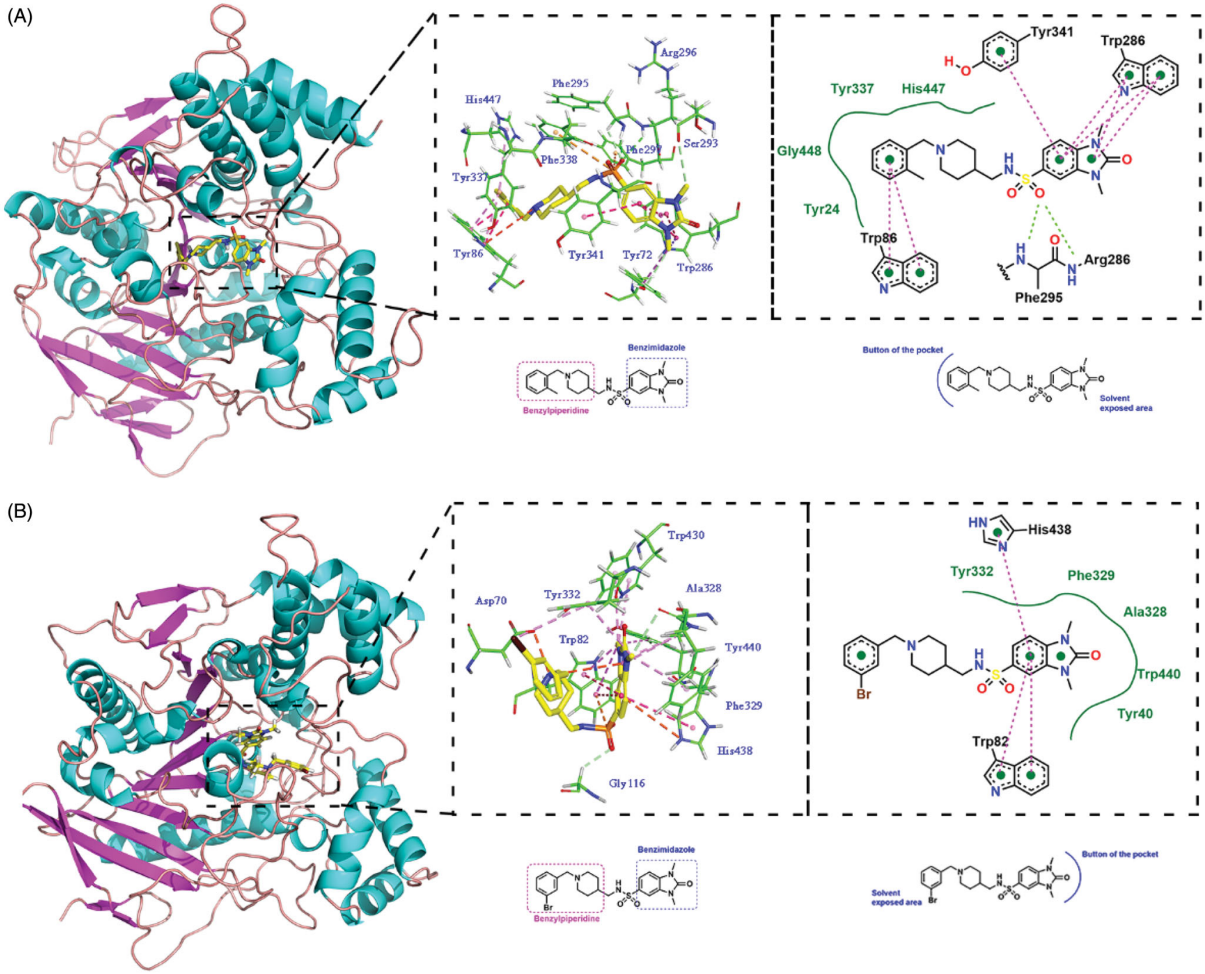
(A) Binding mode prediction of **15b** with huAChE (PDB id: 4EY7). (B) Binding mode prediction of **15j** with huBChE (PDB id: 4TPK). Colour coding: green, hydrogen bond; red, π–π stacking; orange, π–sulphur; purple, π–alkyl.

To further investigate the binding modes of **15b** and **15j** with ChEs, molecular docking was performed using the Discovery Studio software 2016, BIOVIA, San Diego, CA. The predicted binding mode for the compound **15b** was shown in Figure 3(A). **15b** simultaneously occupied both the CAS and PAS of AChE. The benzyl ring of *N*-benzylpiperidine moiety interacted with Trp86 in CAS via aromatic π–π interaction. The benzimidazole ring interacted with Trp286 and Tyr341 in PAS via π–π stacking interactions. The sulphonyl group formed two hydrogen bonds with Phe295 and Arg296. Besides, the sulphur formed two π–sulphur interactions with Phe297 and Phe338. Meanwhile, the methyl groups moiety of **15b** formed some π–alkyl interactions with Tyr72, Ser293, Tyr337, and His447. All these facts provide an explanation for micromolar inhibitory activity of compound **15b** against AChE.

Molecular docking of **15j** in the active site of BChE has been shown in Figure 3(B). **15j** exhibited a U-shaped conformation. The benzimidazole ring interacted with Trp82 and His438 via π–π stacking interactions. The sulphur formed a π–sulphur interaction with Trp82. The charged nitrogen of piperidine ring formed a π–cation interaction with Asp70. And some π–alkyl interactions were formed between Tyr332, Tyr440, Phe329, Trp430, and the methyl, bromine groups in **15j**, which increased inhibitory activities by strengthening the binding affinity.

#### 2.2.4. Cell toxicity studies

**Figure 4.**
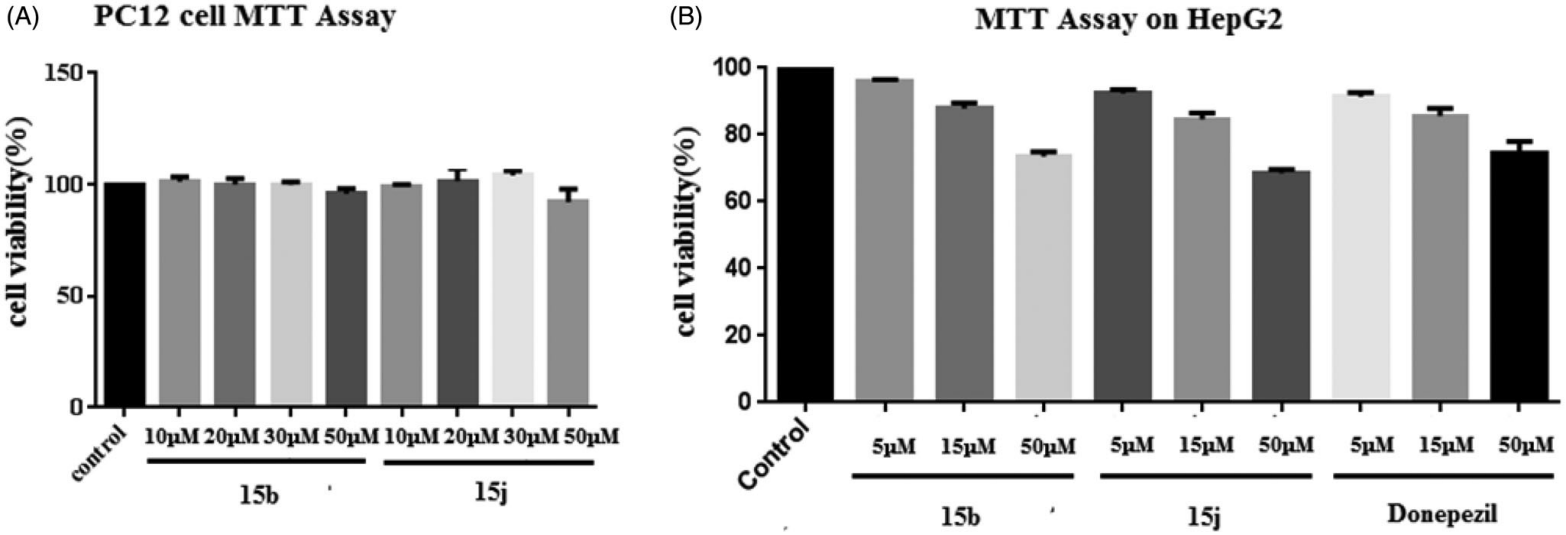
(A) *In vitro* cell toxicity of **15b** and **15j** on PC-12 cell line. (B) *In vitro* hepatotoxicity of **15b** and **15j** on HepG2 cell line. Data were expressed as mean ± SD (*n* = 3).

We focussed on the cell toxicity of the synthesised compounds. Compounds **15b** and **15j** were selected as representative compounds for the evaluation of their potential cytotoxic effects. **15b** and **15j** exhibited no cytotoxicity to PC-12 cells at different concentrations (10, 20, 30, and 50 µM), indicating its low cell toxicity. Results are shown in Figure 4(A).

Next, MTT assay was performed using the human hepatoma cell line HepG2 to evaluate preliminary the hepatotoxicity of **15b** and **15j** *in vitro*, which were incubated with **15b** and **15j** of 5, 15, 50 µM respectively for 24 h. Compared to donepezil, the two compounds showed low toxicity on cell viability (Figure 4(B)).

#### 2.2.5. Inhibition of self-induced Aβ1–42 aggregation

Compounds **15b** and **15j** were evaluated for their inhibitory capacity on self-induced Aβ_1–42_ aggregation based on a thioflavin T-based fluorometric assay. All the tested compounds showed no inhibitory activities under 50 µM (Supplementary Figure S1(A)).

#### 2.2.6. DPPH radical scavenging activity (DPPH)

Compounds **15b** and **15j** were selected to be evaluated for their 1,1-diphenyl-2-picrylhydrazyl radical scavenging activity (DPPH) compared with trolox as reference compound^12^. **15b** showed a better inhibitory rate than (12.2%) **15j** (6.3%) at 1 mM. Therefore, they showed weak antioxidant activities (Supplementary Figure S1(B)).

#### 2.2.7. Neuroprotective effect against H_2_O_2_-induced cell death in PC12 neurons

We evaluated the cytoprotective effects of **15b** and **15j** on H_2_O_2_-induced cell damage. Treatment with 800 µM H_2_O_2_ for 24 h caused over 55% death rate of PC12 cells compared with the control group (Supplementary Figure S2(A)). Then we pre-treated PC12 cells with **15b** and **15j** for 24 h, the mortality rate of PC12 cells caused by H_2_O_2_ was reduced. Such protective effect exhibited dose-dependent manner for both the two compounds. **15b** showed a better cytoprotective effect than **15j**. These results indicated that **15b** and **15j** had a potential in antagonising the oxidative stress.

#### 2.2.8. Behavioural studies

**Figure 5.**
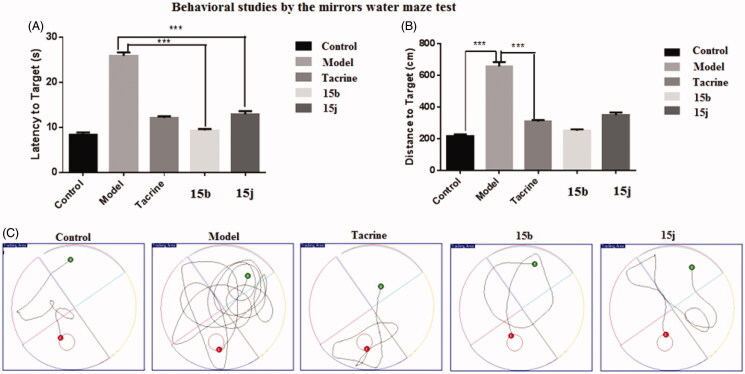
Effects of oral administration of tacrine (15 mg kg^−1^), **15b** (15 mg kg^−1^), and **15j** (15 mg kg^−1^) on scopolamine-induced cognitive impairment in ICR mice determined by the Morris water maze test. (A) The latency to target; (B) the distance to target; and (C) the trajectories of mice. Data are presented as the mean ± SEM (*n* = 6; ***p < 0.001 vs. scopolamine group).

Compound **15b** and **15j** were selected for *in vivo* behavioural study^22^. The ameliorating potential of **15b** and **15j** against scopolamine-induced cognition impairment in ICR mice were investigated in Morris water maze test. Tacrine (20 mmol kg^−1^ body weight) was used as positive control. **15b**, **15j** (15 mg kg^−1^) and tacrine were orally administered to the ICR mice 30 min before intraperitoneal (ip) administration of scopolamine (1 mg kg^−1^) or saline solution for 10 consecutive days to adapt the apparatus. The test included 5 days of learning and memory training and a probe trial on the sixth day. The mean escape latency values of all the groups on the sixth day were shown in Table 2 and Figure 5(A). Compared to the control group, scopolamine led to a remarkable delay of the latency to target (8.34 ± 0.53 s vs. 25.83 ± 0.75 s), indicating that the cognitive impairment mouse model was successfully built. Treatment of tacrine ameliorated the impairment and the latency to target reduced to 12.06 ± 0.37 s. Compared to tacrine, **15b** reduced the latency to target (9.29 ± 0.31 s), but **15j** exhibited a comparable activity (12.85 ± 0.72 s), indicating that they considerably ameliorated the cognitive impairment of the treated mice.

**Table 2.** Effects of oral administration of **15b** and **15j** (15 mg kg^−1^) on scopolamine-induced memory impairment in ICR mice evaluated by the Morris water maze test. Tacrine (15 mg kg^−1^) was used as positive control. Data are presented as the mean ± SEM (*n* = 6).

Group	Latency to target (s)	Distance to target (cm)
Control	8.34 ± 0.53	216.23 ± 13.62
Model	25.83 ± 0.75	657.70 ± 27.28
Tacrine	12.06 ± 0.37	310.01 ± 9.54
**15b**	9.29 ± 0.31	252.32 ± 8.42
**15j**	12.85 ± 0.72	350.27 ± 16.59

The distance to target (Table 2; Figure 5(C)) and the trajectories of the mice in each group were also analysed. Compared to the control group, the administration of scopolamine remarkably led to the extended distance to target (657.70 ± 27.28 cm vs. 216.23 ± 13.62 cm). When treated with tacrine, **15b** and **15j** the distance to target were significantly shortened. These results were supported by trajectory analysis. As shown in Figure 5(C), the trajectory of the mice in scopolamine model group was very long and disordered, while tacrine, **15b** and **15j** groups showed shortened distances. Mice treated with **15b** and **15j** almost recovered to the normal cognition, with a similar orientation and distance to that of the normal mice. Taken together, these results supported that **15b** and **15j** remarkably ameliorated the cognition impairment caused by scopolamine.

#### 2.2.9. Hepatotoxicity studies

**Figure 6.**
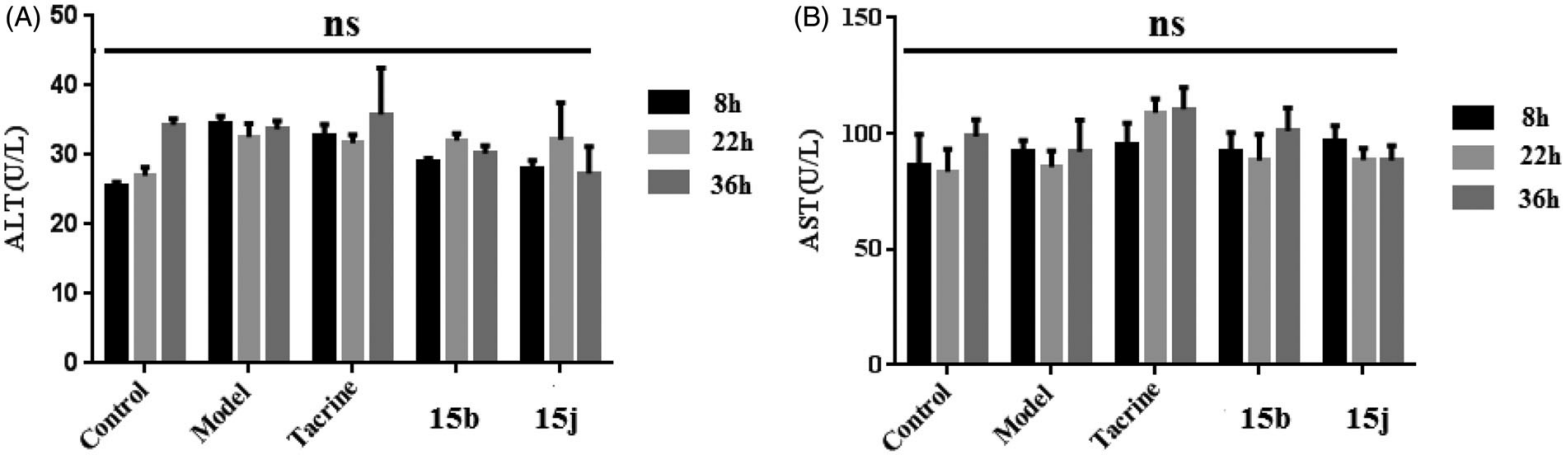
The ALT and AST levels. (A) The ALT levels of five subgroups. (B) The AST levels of five subgroups. Data are presented as the mean ± SEM (*n* = 6; ns *p* > 0.05).

We next investigated the possible drug-induced hepatotoxicity by comparing their toxic profile to tacrine in the *in vivo* assays. The alanine aminotransferase (ALT) and aspartate aminotransferase (AST) levels were measured, as shown in Table 3 and Figure 6. Heparinised serum was obtained after the treatment of **15b** and **15j** at 8, 22, and 36 h, respectively. The serum levels of ALT and AST are directly proportional to the severity of the liver damage. Compared to the control group, after the treatment of **15b** and **15j**, the levels of ALT and AST were slightly induced at 22 h, but in general, no remarkable damage was observed. The level of ALT and AST slightly reduced at 36 h, compared to those of tacrine group, which suggested that **15b** and **15j** had preliminary safety.

**Table 3.** ALT and AST activity after the administration of **15b** and **15j**. Tacrine (30 mg kg^−1^) was used as the reference compound. Values are expressed as the mean ± SEM of six independent experiments.

	ALT(U/L)		
Group	8 h	22 h	36 h
Control	25.4 ± 0.7	27.0 ± 1.2	34.3 ± 0.9
Model	34.4 ± 1.2	32.6 ± 1.9	33.7 ± 1.2
Tacrine	32.7 ± 1.7	31.7 ± 1.2	35.8 ± 6.7
**15b**	29.1 ± 0.4	32.1 ± 1.0	30.2 ± 1.1
**15j**	28.1 ± 1.1	32.2 ± 5.3	27.3 ± 3.9
	AST(U/L)		
Group	8 h	22 h	36 h
Control	86.4 ± 13.3	83.4 ± 9.8	98.7 ± 7.4
Model	91.9 ± 5.0	85.6 ± 6.8	92.1 ± 13.7
Tacrine	95.1 ± 9.5	108.9 ± 6.1	110.4 ± 9.4
**15b**	92.1 ± 8.3	88.4 ± 11.2	101.0 ± 10.1
**15j**	96.5 ± 6.8	88.6 ± 4.9	88.4 ± 6.3

**Figure 7.**
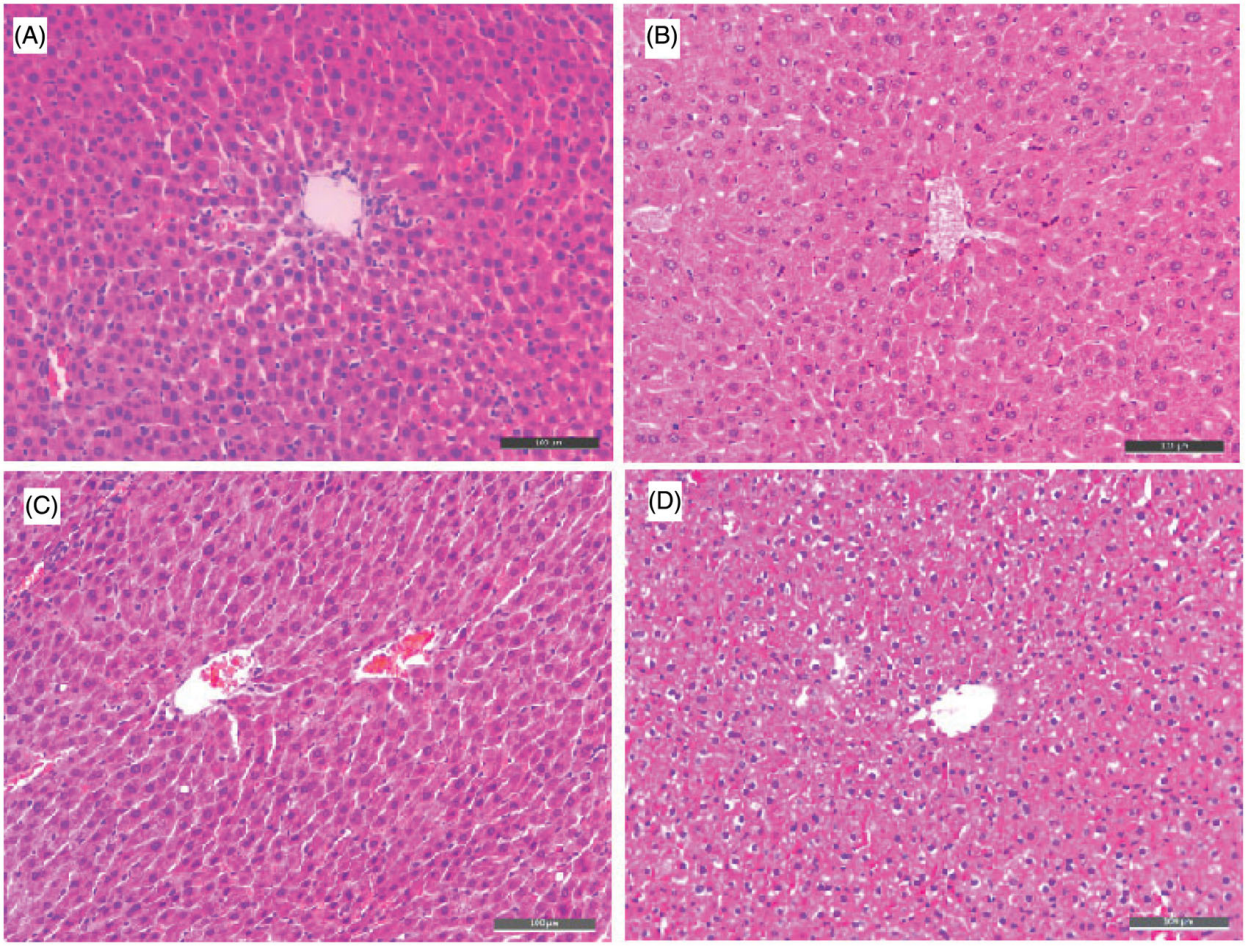
Histopathological study of livers of male mice after treatment with the solvent only (control, A), or 36 h after administration of tacrine (B), **15b** (C), or **15j** (D). HE staining, original magnification ×200.

To further analyse, the hepatotoxicity of **15b** and **15j**, morphologic studies by immunohistochemical staining were applied. Treatment of tacrine (Figure 7(B)), **15b** (Figure 7(C)) or **15j** (Figure 7(D)) did not result in remarkable morphologic changes in liver compared to the control group (Figure 7(A)). In summary, the results indicated that **15b** and **15j** had a low hepatotoxicity profile.

## 3. Conclusion

In summary, in the present studies, a series of benzylpiperidine-linked 1,3-dimethylbenzimidazolinone derivatives were designed and synthesised for the treatment of AD. *In vitro* assays proved that most of the compounds effectively inhibited ChEs in the micromolar range. **15b** and **15j** showed the most potent activity (**15b**, anti-AChE IC_50_ = 0.39 ± 0.11 µM, anti-BChE IC_50_ = 0.66 ± 0.16 µM; **15j**, anti-AChE IC_50_ = 0.39 ± 0.15 µM, anti-BChE IC_50_ = 0.16 ± 0.04 µM). Kinetic studies indicated the competitive inhibition manner of **15b** and **15j** on AChE and BChE. Cell toxicity assay using HepG2 cells showed compounds **15b** and **15j** had a lower hepatotoxicity profile with respect to donepezil. In addition, no significant increases in ALT and AST levels were observed after the administration of **15b** and **15j** in mice. Furthermore, administration of **15b** and **15j** considerably ameliorated the cognition impairment in the scopolamine treated ICR mice in Morris water maze test. **15b** exhibited cytoprotective effects against H_2_O_2_ induced cell damage. **15b** and **15j** also showed antioxidant activity according to the DPPH assay. Finally, **15b** and **15j** can be a promising lead compound for further development of newer anti-AD drugs.

## 4. Experimental section

### 4.1. Chemistry

All the chemicals were purchased from commercial companies and used without purification. All reactions were monitored by analytical thin layer chromatography (TLC) on silica gel 60 F254 precoated plates (purchased from Qingdao Haiyang Inc., Qingdao, China). Visualisation was achieved using UV light (254 nm and 365 nm), Flash column chromatography was performed with silica gel (200–300 mesh) purchased from Qingdao Haiyang Chemical Co. Ltd. Melting points were determined using a Mel - TEMP II melting point apparatus. ^1^H NMR spectra are recorded on Bruker AV-500 or AV-300 MHz instruments in CDCl_3_ or DMSO-d_6_. Chemical shifts are reported in parts per million (d) downfield from the signal of tetramethylsilane (TMS) as the internal standard. Coupling constants are reported in Hz. All chemical shifts are reported in parts per million (ppm), relative to the internal standard. The following abbreviations are used to indicate the multiplicities of the respective signals: s – singlet; bs – broad singlet; d – doublet; dd – doublet of doublets; t – triplet; and m – multiplet. High-resolution mass spectrometry (HRMS) was performed on a Mariner Mass Spectrum (ESI) or an LC/MSD TOF HR-MS Spectrum.

#### 4.1.1. General procedure for the synthesis of intermediates 4a–4p

To a solution of 4-piperidinecarboxamide (**1**) (2.0 g, 15.6 mmol) in methanol, potassium carbonate (4.3 g, 31.2 mmol) and substituted benzyl bromide (**2a–2p,** 1.5 equiv.) were added, and the reaction mixture was refluxed for 6–8 h. Solvent was distilled off, and addition of crushed ice to the residue led to the formation of white Coloured precipitates of 1-(substituted benzyl)-piperidine-4-carboxamides (**3a**–**3p**)^5^. Then, to a suspension of **3a**–**3p** (1.0 g, 4.5 mmol) in dry THF (20 ml) was added slowly to a solution of LiAlH_4_ (0.8 g, 22.9 mmol, 5 equiv.) in dry THF (30 ml). The mixture was stirred under reflux and argon atmosphere for 4 h. After cooling, water was added at 0 °C, the precipitate was filtered and washed with Et_2_O. The filtrate was extracted with Et_2_O (×2), dried over MgSO_4_ and the solvent was evaporated under vacuum to afford compounds **4a**–**4p** as a white solid^23^.

#### 4.1.2. General procedure for the synthesis of intermediate 6

To a solution of methyl 3,4-diaminobenzoate (**5**) (1.0 g, 0.6 mmol, 1.0 equiv.) in chloroform (12 ml), di(1*H*-imidazol-1-yl)methanone (1.4 g, 9.0 mmol, 1.5 equiv.) was added. The mixture was heated to 60 °C for 12 h under a nitrogen atmosphere. After cooling the reaction to room temperature, the white precipitate was collected, washed with chloroform (8 ml × 2), and dried *in vacuo* to give intermediate **6** (0.8 g, 68%) as a white solid^24^. 1H NMR (300 MHz, DMSO-d_6_): *δ* 11.03 (s, 1H), 10.87 (s, 1H), 7.63 (dd, *J_1_* = 8.2, *J_2_* = 1.6 Hz, 1H), 7.47 (s, 1H), 7.02 (d, *J* = 8.2 Hz, 1H), 3.82 (s, 3H).

#### 4.1.3. General procedure for the synthesis of intermediate 7

To a stirred solution of intermediate **6** (1.0 g, 5.2 mmol, 1.0 equiv.) in DMF (10 ml) at 0 °C, NaH (60% dispersion in mineral oil, 1.8 g, 13.0 mmol, 2.5 equiv.) was added, and the mixture was stirred for 15 min. Methyl iodide (1.8 g, 13.0 mmol, 2.5 equiv.) was added dropwise, and the mixture was stirred at room temperature for an additional 12 h. Water (30 ml) was added and extracted with EtOAc (30 ml × 3). The combined organic layers were dried over anhydrous Na_2_SO_4_, filtered, and concentrated *in vacuo*. The crude residue was purified by silica gel chromatography (petroleum ether/EtOAc  =  8:1) to give intermediate **7** (0.7 g, 60%) as a white solid^25^. ^1^H NMR (300 MHz, DMSO-d_6_): *δ* 7.74 (dd, *J_1_* = 8.2, *J_2_* = 1.6 Hz, 1H), 7.66 (s, 1H), 7.23 (d, *J* = 8.2 Hz, 1H), 3.85 (s, 3H), 3.36 (d, *J* = 3.6 Hz, 6H).

#### 4.1.4. General procedure for the synthesis of intermediate 8

To a solution of the intermediate **7** (1.0 g, 4.5 mmol, 1.0 equiv.) in THF (10 ml)/H_2_O (10 ml), LiOH (0.5 g, 22.7 mmol, 5.0 equiv.) was added, and the mixture was stirred at 50 °C for 2 h, followed by evaporation of THF under reduced pressure. To the residue solution, 10 ml H_2_O was added, and then 6 N hydrochloric acid was utilised to adjust the pH to 2.0–3.0. The resulting precipitate was collected by filtration, washed with water and dried to afford the intermediate **8** as white solid (0.85 g, 94%)^26^. 1H NMR (300 MHz, DMSO-d_6_): *δ* 12.69 (s, 1H), 7.73 (dd, *J_1_* = 8.2, *J_2_* = 1.5 Hz, 1H), 7.65 (d, *J* = 1.3 Hz, 1H), 7.20 (d, *J* = 8.2 Hz, 1H), 3.36 (d, *J* = 3.6 Hz, 6H).

#### 4.1.5. General procedure for the synthesis of target compounds 9a–9p

Compound **8** (0.2 g, 0.9 mmol, 1.0 equiv.) and PyBOP (0.6 g, 1.2 mmol, 1.3 equiv.) and DIPEA (0.2 g, 1.9 mmol, 2.0 equiv.) were added to 10 ml of DMF and stirred at room temperature for 20 min. Then, intermediates **4a–4p** (1.2 equiv.) was added and stirred at room temperature for 4 h. After completion of the reaction, the reaction mixture was quenched with water. The aqueous phase was extracted with CH_2_Cl_2_ (3 × 15 ml). The CH_2_Cl_2_ layer was then washed with brine and dried over anhydrous Na_2_SO_4_. After concentration, the crude product was purified by silica gel column chromatograph (CH_2_Cl_2_/methanol  =  80:1) to give target compounds **9a–9p**.

##### 4.1.5.1. N-((1-benzylpiperidin-4-yl)methyl)-1,3-dimethyl-2-oxo-2,3-dihydro-1H-benzo[d]imidazole-5-carboxamide (9a)

White solid, yield: 66%, m.p. 121–123 °C. ^1^H NMR (300 MHz, CDCl_3_): *δ* 7.52 (d, *J* = 1.4 Hz, 1H), 7.47 (dd, *J_1_* = 8.1, *J_2_* = 1.6 Hz, 1H), 7.33 (d, *J* = 4.5 Hz, 4H), 6.97 (d, *J* = 8.1 Hz, 1H), 6.26 (s, 1H), 3.52 (s, 2H), 3.46 (d, *J* = 2.4 Hz, 6H), 3.40 (t, *J* = 6.1 Hz, 2H), 2.94 (d, *J* = 11.7 Hz, 2H), 2.00 (dd, *J_1_* = 11.7, *J_2_* = 9.6 Hz, 2H), 1.75 (s, 4H), 1.42 (td, *J* = 12.1, 3.5 Hz, 2H). ^13 ^C NMR (126 MHz, DMSO-d_6_): δ 166.68, 154.57, 132.41, 129.85, 128.88, 127.87, 121.16), 107.34, 107.08, 52.80, 44.84, 27.58, 27.48. HRMS (ESI): calcd. for C_23_H_28_N_4_O_2_ [M + H]^+^ 393.2290, found 393.2283.

##### 4.1.5.2. 1,3-dimethyl-N-((1–(2-methylbenzyl)piperidin-4-yl)methyl)-2-oxo-2,3-dihydro-1H-benzo[d]imidazole-5-carboxamide (9b)

White solid; yield: 58%, m.p. 131–133 °C. ^1^H NMR (300 MHz, DMSO-d_6_): *δ* 8.92 (s, 1H), 8.49 (d, *J* = 6.0 Hz, 1H), 7.66 (d, *J* = 8.0 Hz, 2H), 7.48 (d, *J* = 7.2 Hz, 1H), 7.33 (dd, *J_1_* = 11.6, *J_2_* = 6.6 Hz, 2H), 7.21 (d, *J* = 8.1 Hz, 1H), 4.30 (d, *J* = 5.2 Hz, 2H), 3.36 (d, *J* = 1.8 Hz, 6H), 3.21 (s, 2H), 3.09 (s, 2H), 2.40 (s, 3H), 1.89 (d, *J* = 12.8 Hz, 3H), 1.44 (d, *J* = 12.9 Hz, 2H), 1.23 (d, *J* = 1.7 Hz, 2H). ^13 ^C NMR (126 MHz, DMSO-d_6_): δ 166.78, 154.56, 138.98, 132.37, 131.36, 130.08, 129.86, 128.84, 127.74, 126.67, 121.18, 107.19, 57.25, 52.30, 46.32, 44.35, 34.26, 27.51, 26.37, 19.78. HRMS (ESI): calcd. for C_24_H_30_N_4_O_2_ [M + H]^+^ 407.2447, found 407.2441.

##### 4.1.5.3. 1,3-dimethyl-N-((1–(3-methylbenzyl)piperidin-4-yl)methyl)-2-oxo-2,3-dihydro-1H-benzo[d]imidazole-5-carboxamide (9c)

White solid, yield: 78%, m.p. 109–110 °C. ^1^H NMR (300 MHz, DMSO-d_6_): *δ* 9.11 (s, 1H), 8.45 (t, *J* = 5.7 Hz, 1H), 7.65 (d, *J* = 7.6 Hz, 2H), 7.36 (d, *J* = 7.3 Hz, 1H), 7.29 (d, *J* = 5.4 Hz, 2H), 7.22 (d, *J* = 8.8 Hz, 1H), 4.24 (d, *J* = 5.0 Hz, 2H), 3.37 (d, *J* = 1.8 Hz, 6H), 3.21 (d, *J* = 6.1 Hz, 2H), 2.92 (d, *J* = 12.1 Hz, 2H), 2.35 (s, 3H), 1.88 (d, *J* = 14.1 Hz, 3H), 1.74 (s, 2H), 1.40 (d, *J* = 11.8 Hz, 2H). ^13 ^C NMR (126 MHz, DMSO-d_6_): *δ* 166.80, 154.56, 138.60, 132.48, 132.19, 130.64, 129.86, 129.21, 128.77, 127.73, 121.17, 107.20, 59.83, 52.04, 46.34, 44.31, 34.26, 27.52, 21.36. HRMS (ESI): calcd. for C_24_H_30_N_4_O_2_ [M + H]^+^ 407.2447, found 407.2440.

##### 4.1.5.4. 1,3-dimethyl-N-((1–(4-methylbenzyl)piperidin-4-yl)methyl)-2-oxo-2,3-dihydro-1H-benzo[d]imidazole-5-carboxamide (9d)

White solid, yield: 85%, m.p. 151–153 °C. ^1^H NMR (300 MHz, CDCl_3_): *δ* 7.52 (d, *J* = 1.4 Hz, 1H), 7.48 (dd, *J_1_* = 8.1, *J_2_* = 1.5 Hz, 1H), 7.21 (d, *J* = 8.0 Hz, 2H), 7.13 (d, *J* = 8.1 Hz, 2H), 6.95 (d, *J* = 8.1 Hz, 1H), 6.38 (s, 1H), 3.54 (s, 2H), 3.45 (s, 6H), 3.38 (t, *J* = 6.1 Hz, 2H), 2.98 (d, *J* = 11.6 Hz, 2H), 2.35 (s, 3H), 2.02 (dd, *J_1_* = 19.6, *J_2_* = 7.4 Hz, 3H), 1.76 (d, *J* = 12.7 Hz, 2H), 1.45 (td, *J* = 12.2, 3.3 Hz, 2H). ^13 ^C NMR (126 MHz, DMSO-d_6_): *δ* 166.59, 154.55, 136.31, 135.64, 132.33, 129.81, 129.23, 129.13, 127.95, 121.17, 107.19, 62.51, 53.31, 45.31, 36.21, 30.20, 27.50, 21.15. HRMS (ESI): calcd. for C_24_H_30_N_4_O_2_ [M + H]^+^ 407.2447, found 407.2454.

##### 4.1.5.5. N-((1–(3,4-dimethylbenzyl)piperidin-4-yl)methyl)-1,3-dimethyl-2-oxo-2,3-dihydro-1H-benzo[d]imidazole-5-carboxamide (9e)

White solid, yield: 64%, m.p. 164–165 °C. ^1^H NMR (300 MHz, DMSO-d_6_): *δ* 9.07 (s, 1H), 8.45 (d, *J* = 5.9 Hz, 1H), 7.65 (d, *J* = 7.4 Hz, 1H), 7.28 – 7.13 (m, 4H), 4.19 (d, *J* = 5.0 Hz, 2H), 3.36 (d, *J* = 1.8 Hz, 6H), 3.23 – 3.15 (m, 2H), 2.90 (d, *J* = 12.4 Hz, 2H), 2.25 (s, 6H), 1.88 (d, *J* = 13.8 Hz, 3H), 1.40 (d, *J* = 12.3 Hz, 2H), 1.21 (d, *J* = 18.4 Hz, 3H). ^13 ^C NMR (126 MHz, DMSO-d_6_): *δ* 166.79, 154.56, 138.34, 137.21, 132.63, 132.48, 130.31, 129.87, 129.14, 127.73, 127.42, 121.16, 107.36, 107.06, 59.64, 51.92, 44.32, 34.27, 27.59, 27.48, 27.33, 19.83, 19.62. HRMS (ESI): calcd. for C_25_H_32_N_4_O_2_ [M + H]^+^ 421.2603, found 421.2607.

##### 4.1.5.6. N-((1–(3-methoxybenzyl)piperidin-4-yl)methyl)-1,3-dimethyl-2-oxo-2,3-dihydro-1H-benzo[d]imidazole-5-carboxamide (9f)

White solid, yield: 56%, m.p. 178–179 °C. ^1^H NMR (300 MHz, DMSO-d_6_): *δ* 9.12 (s, 1H), 8.45 (t, *J* = 6.0 Hz, 1H), 7.65 (d, *J* = 7.6 Hz, 2H), 7.40 (t, *J* = 7.8 Hz, 1H), 7.22 (d, *J* = 8.7 Hz, 1H), 7.09 – 7.02 (m, 2H), 4.25 (d, *J* = 4.9 Hz, 2H), 3.80 (s, 3H), 3.37 (d, *J* = 1.9 Hz, 6H), 3.21 (t, *J* = 6.0 Hz, 2H), 2.93 (d, *J* = 12.4 Hz, 2H), 1.89 (d, *J* = 13.8 Hz, 2H), 1.39 (t, *J* = 12.4 Hz, 2H), 1.26 (d, *J* = 10.7 Hz, 3H). ^13 ^C NMR (126 MHz, DMSO-d_6_): *δ* 166.80, 159.85, 154.57, 132.49, 130.48, 129.88, 127.73, 123.66, 121.17, 117.20, 115.38, 107.36, 107.06, 55.67, 52.11, 46.34, 34.28, 32.00, 29.89, 27.54. HRMS (ESI): calcd. for C_24_H_30_N_4_O_2_ [M + H]^+^ 423.2396, found 423.2394.

##### 4.1.5.7. N-((1–(4-methoxybenzyl)piperidin-4-yl)methyl)-1,3-dimethyl-2-oxo-2,3-dihydro-1H-benzo[d]imidazole-5-carboxamide (9g)

White solid, yield: 67%, m.p. 111–113 °C. ^1^H NMR (300 MHz, DMSO-d_6_): *δ* 9.03 (s, 1H), 8.45 (d, *J* = 5.7 Hz, 1H), 7.64 (s, 1H), 7.41 (d, *J* = 8.6 Hz, 2H), 7.21 (d, *J* = 8.8 Hz, 1H), 7.03 (d, *J* = 8.6 Hz, 2H), 4.21 (d, *J* = 4.6 Hz, 2H), 3.79 (s, 3H), 3.36 (d, *J* = 1.8 Hz, 6H), 3.20 (t, *J* = 5.9 Hz, 2H), 2.89 (d, *J* = 11.6 Hz, 2H), 1.85 (t, *J* = 20.0 Hz, 4H), 1.38 (d, *J* = 10.9 Hz, 2H), 1.20 (dd, *J_1_* = 15.9, *J_2_* = 8.6 Hz, 1H). ^13 ^C NMR (126 MHz, DMSO-d_6_): *δ* 166.79, 160.57, 154.56, 133.22, 132.48, 129.87, 127.73, 121.16, 114.65, 107.35, 107.06, 59.29, 55.69, 51.71, 44.31, 34.30, 27.53, 27.36. HRMS (ESI): calcd. for C_24_H_30_N_4_O_2_ [M + H]^+^ 423.2396, found 423.2395.

##### 4.1.5.8. N-((1–(2-fluorobenzyl)piperidin-4-yl)methyl)-1,3-dimethyl-2-oxo-2,3-dihydro-1H-benzo[d]imidazole-5-carboxamide (9h)

White solid, yield: 78%, m.p. 122–123 °C. ^1^H NMR (300 MHz, DMSO-d_6_): *δ* 8.36 (s, 1H), 7.83 – 7.65 (m, 2H), 7.38 (d, *J* = 25.7 Hz, 2H), 7.24 (t, *J* = 23.5 Hz, 3H), 3.52 (s, 2H), 3.36 (d, *J* = 3.1 Hz, 6H), 3.16 (d, *J* = 5.6 Hz, 2H), 2.86 (s, 2H), 1.96 (s, 2H), 1.67 (s, 3H), 1.24 (d, *J* = 4.4 Hz, 2H). ^13 ^C NMR (126 MHz, DMSO-d_6_): *δ* 166.64, 162.26, 160.32, 154.56, 132.37, 129.84, 127.92, 124.73, 121.16, 115.78, 115.60, 107.31, 107.08, 53.01, 45.05, 35.70, 29.77, 27.56, 27.47. HRMS (ESI): calcd. for C_23_H_27_FN_4_O_2_ [M + H]^+^ 411.2196, found 411.2191.

##### 4.1.5.9. N-((1–(3-fluorobenzyl)piperidin-4-yl)methyl)-1,3-dimethyl-2-oxo-2,3-dihydro-1H-benzo[d]imidazole-5-carboxamide (9i)

White solid, yield: 78%, m.p. 128–130 °C. ^1^H NMR (300 MHz, DMSO-d_6_): *δ* 8.36 (t, *J* = 5.6 Hz, 1H), 7.66 (d, *J* = 4.9 Hz, 2H), 7.35 (dd, *J_1_* = 14.3, *J_2_* = 7.8 Hz, 1H), 7.20 (d, *J* = 8.6 Hz, 1H), 7.10 (dd, *J_1_* = 17.3, *J_2_* = 8.1 Hz, 3H), 3.46 (s, 2H), 3.36 (d, *J* = 3.4 Hz, 6H), 3.17 (t, *J* = 6.0 Hz, 2H), 2.79 (d, *J* = 11.2 Hz, 2H), 1.91 (t, *J* = 10.9 Hz, 2H), 1.63 (t, *J* = 16.9 Hz, 3H), 1.21 (d, *J* = 11.6 Hz, 2H). ^13 ^C NMR (126 MHz, DMSO-d_6_): *δ* 166.58, 163.65, 161.72, 154.55, 142.47, 132.34, 130.45, 129.82, 127.96, 124.99, 121.15, 115.51, 114.04, 107.30, 107.09, 62.13, 53.43, 45.33, 36.23, 30.36, 27.55, 27.46. HRMS (ESI): calcd. for C_23_H_27_FN_4_O_2_ [M + H]^+^ 411.2196, found 411.2190.

##### 4.1.5.10. N-((1–(4-fluorobenzyl)piperidin-4-yl)methyl)-1,3-dimethyl-2-oxo-2,3-dihydro-1H-benzo[d]imidazole-5-carboxamide (9j)

White solid, yield: 82%, m.p. 165–167 °C. ^1^H NMR (300 MHz, CDCl_3_): *δ* 7.52 (s, 1H), 7.47 (dd, *J_1_* = 8.2, *J_2_* = 1.5 Hz, 1H), 7.26 (s, 1H), 6.99 (dd, *J* = 16.1, 8.3 Hz,: 4H), 6.27 (s, 1H), 3.47 (s, 2H), 3.46 (d, *J* = 1.9 Hz, 6H), 3.39 (t, *J* = 6.2 Hz, 2H), 2.88 (s, 2H), 1.97 (t, *J* = 10.6 Hz, 2H), 1.85 – 1.71 (m, 3H), 1.46 – 1.32 (m, 2H). ^13 ^C NMR (126 MHz, DMSO-d_6_): *δ* 166.57, 162.57, 160.64, 154.56, 135.32, 132.35, 130.94, 130.88, 129.83, 127.97, 121.16, 115.33, 115.16, 107.31, 107.10, 61.96, 53.36, 45.34, 36.28, 30.35, 27.57, 27.47. HRMS (ESI): calcd. for C_23_H_27_FN_4_O_2_ [M + H]^+^ 411.2196, found 411.2190.

##### 4.1.5.11. N-((1–(2-chlorobenzyl)piperidin-4-yl)methyl)-1,3-dimethyl-2-oxo-2,3-dihydro-1H-benzo[d]imidazole-5-carboxamide (9k)

White solid, yield: 90%, m.p. 156–158 °C. ^1^H NMR (300 MHz, DMSO-d_6_): *δ* 9.17 (s, 1H), 8.46 (s, 1H), 7.68 (d, *J* = 2.5 Hz, 1H), 7.64 (s, 1H), 7.60 (s, 1H), 7.55–7.48 (m, 2H), 7.22 (d, *J* = 8.6 Hz, 1H), 4.43 (d, *J* = 5.0 Hz, 2H), 3.44 (d, *J* = 11.8 Hz, 2H), 3.37 (d, *J* = 2.0 Hz, 6H), 3.21 (s, 2H), 1.89 (d, *J* = 12.6 Hz, 3H), 1.45 (d, *J* = 11.5 Hz, 2H), 1.21 (d, *J* = 17.9 Hz, 2H). ^13 ^C NMR (126 MHz, DMSO-d_6_): *δ* 166.78, 154.57, 135.30, 134.22, 132.49, 132.18, 130.43, 129.88, 128.21, 127.72, 121.18, 107.37, 107.06, 56.86, 52.54, 46.38, 33.99, 27.61, 27.50, 26.40, 26.34. HRMS (ESI): calcd. for C_23_H_27_ClN_4_O_2_ [M + H]^+^ 427.1901, found 427.1892.

##### 4.1.5.12. N-((1–(3-chlorobenzyl)piperidin-4-yl)methyl)-1,3-dimethyl-2-oxo-2,3-dihydro-1H-benzo[d]imidazole-5-carboxamide (9l)

White solid, yield: 76%, m.p. 134–136 °C. ^1^H NMR (300 MHz, DMSO-d_6_): δ 8.36 (t, *J* = 5.7 Hz, 1H), 7.65 (dd, *J_1_* = 4.2, *J_2_* = 2.7 Hz, 2H), 7.36 – 7.32 (m, 2H), 7.31 (s, 1H), 7.27 (s, 1H), 7.20 (d, *J* = 8.6 Hz, 1H), 3.45 (s, 2H), 3.37 (s, 3H), 3.35 (s, 3H), 3.17 (t, *J* = 6.2 Hz, 2H), 2.78 (d, *J* = 11.3 Hz, 2H), 1.91 (t, *J* = 10.6 Hz, 2H), 1.63 (t, *J* = 17.1 Hz, 3H), 1.27 – 1.16 (m, 2H). ^13 ^C NMR (126 MHz, DMSO-d_6_): *δ* 166.57, 154.56, 141.99, 133.33, 132.34, 130.46, 129.82, 128.69, 127.94, 127.72, 127.20, 121.16, 107.34, 107.12, 62.02, 53.41, 45.32, 36.21, 30.35, 27.58, 27.48. HRMS (ESI): calcd. for C_23_H_27_ClN_4_O_2_ [M + H]^+^ 427.1901, found 427.1899.

##### 4.1.5.13. N-((1–(4-chlorobenzyl)piperidin-4-yl)methyl)-1,3-dimethyl-2-oxo-2,3-dihydro-1H-benzo[d]imidazole-5-carboxamide (9m)

White solid, yield: 75%, m.p. 127–129 °C. ^1^H NMR (300 MHz, DMSO-d_6_): δ 8.36 (t, *J* = 5.6 Hz, 1H), 7.65 (dd, *J_1_* = 4.3, *J_2_* = 2.6 Hz, 1H), 7.37 (d, *J* = 8.4 Hz, 2H), 7.31 (d, *J* = 8.6 Hz, 2H), 7.20 (d, *J* = 8.6 Hz, 1H), 3.42 (s, 2H), 3.37 (s, 3H), 3.35 (s, 3H), 3.17 (t, *J* = 6.2 Hz, 2H), 2.78 (d, *J* = 11.2 Hz, 2H), 1.90 (t, *J* = 10.5 Hz, 2H), 1.65 (d, *J* = 12.7 Hz, 3H), 1.22 (dd, *J_1_* = 14.9, *J_2_* = 8.5 Hz, 3H). ^13 ^C NMR (126 MHz, DMSO-d_6_): *δ* 166.57, 154.56, 138.30, 132.35, 131.69, 130.87, 129.83, 128.52, 127.96, 121.16, 107.21, 61.94, 53.40, 45.33, 36.25, 30.36, 27.53. HRMS (ESI): calcd. for C_23_H_27_ClN_4_O_2_ [M + H]^+^ 427.1901, found 427.1894.

##### 4.1.5.14. N-((1–(2-bromobenzyl)piperidin-4-yl)methyl)-1,3-dimethyl-2-oxo-2,3-dihydro-1H-benzo[d]imidazole-5-carboxamide (9n)

White solid, yield: 75%, m.p. 137–139 °C. ^1^H NMR (300 MHz, DMSO-d_6_): δ 8.37 (t, *J* = 5.5 Hz, 1H), 7.66 (dd, *J_1_* = 4.2, *J_2_* = 2.7 Hz, 2H), 7.58 (d, *J* = 8.0 Hz, 1H), 7.50 – 7.45 (m, 1H), 7.37 (t, *J* = 7.4 Hz, 1H), 7.19 (dd, *J_1_* = 8.1, *J_2_* = 4.8 Hz, 2H), 3.51 (s, 2H), 3.37 (s, 3H), 3.36 (s, 3H), 3.18 (t, *J* = 6.2 Hz, 2H), 2.83 (d, *J* = 11.0 Hz, 2H), 2.02 (t, *J* = 10.7 Hz, 2H), 1.65 (t, *J* = 14.9 Hz, 3H), 1.22 (d, *J* = 9.5 Hz, 2H). ^13 ^C NMR (126 MHz, DMSO-d_6_): *δ* 166.58, 154.57, 138.16, 132.89, 132.35, 131.11, 129.83, 129.18, 128.00, 127.94, 124.28, 121.17, 107.36, 107.13, 61.95, 53.60, 45.31, 36.20, 30.39, 27.59, 27.49. HRMS (ESI): calcd. for C_23_H_27_BrN_4_O_2_ [M + H]^+^ 471.1395, found 471.1384.

##### 4.1.5.15. N-((1–(3-bromobenzyl)piperidin-4-yl)methyl)-1,3-dimethyl-2-oxo-2,3-dihydro-1H-benzo[d]imidazole-5-carboxamide (9o)

White solid, yield: 78%, m.p. 143–145 °C. ^1^H NMR (300 MHz, DMSO-d_6_): δ 8.36 (t, *J* = 5.6 Hz, 1H), 7.65 (dd, *J_1_* = 4.2, *J_2_* = 2.7 Hz, 2H), 7.48 (s, 1H), 7.42 (s, 1H), 7.29 (d, *J* = 6.1 Hz, 2H), 7.20 (d, *J* = 8.6 Hz, 1H), 3.44 (s, 2H), 3.37 (s, 3H), 3.35 (s, 3H), 3.17 (t, *J* = 6.1 Hz, 2H), 2.78 (d, *J* = 11.2 Hz, 2H), 1.91 (t, *J* = 10.7 Hz, 2H), 1.63 (t, *J* = 17.2 Hz, 3H), 1.23 (dd, *J_1_* = 14.0, *J_2_* = 9.1 Hz, 2H). ^13 ^C NMR (126 MHz, DMSO-d_6_): *δ* 166.57, 154.57, 142.28, 132.35, 131.59, 130.77, 130.10, 129.84, 128.11, 127.97, 122.01, 121.16, 107.33, 107.11, 61.98, 53.40, 45.32, 36.21, 30.35, 27.58, 27.48. HRMS (ESI): calcd. for C_23_H_27_BrN_4_O_2_ [M + H]^+^ 471.1395, found 471.1386.

##### 4.1.5.16. N-((1–(4-bromobenzyl)piperidin-4-yl)methyl)-1,3-dimethyl-2-oxo-2,3-dihydro-1H-benzo[d]imidazole-5-carboxamide (9p)

White solid, yield: 69%, m.p. 165–167 °C. ^1^H NMR (300 MHz, CDCl_3_): *δ* 7.50 (d, *J* = 9.3 Hz, 1H), 7.46–7.40 (m, 2H), 7.32 (d, *J* = 4.4 Hz, 2H), 7.20 (d, *J* = 8.3 Hz, 1H), 6.96 (d, *J* = 8.1 Hz, 1H), 6.30 (s, 1H), 3.52 (s, 2H), 3.45 (d, *J* = 1.6 Hz, 6H), 3.39 (t, *J* = 6.2 Hz, 2H), 2.98–2.85 (m, 2H), 2.04– 1.87 (m, 3H), 1.75 (d, *J* = 13.0 Hz, 2H), 1.38 (dd, *J_1_* = 13.6, *J_2_* = 9.4 Hz, 2H). ^13 ^C NMR (126 MHz, DMSO-d_6_): *δ* 166.57, 154.56, 138.71, 132.34, 131.44, 131.26, 129.83, 129.15, 128.55, 127.97, 127.22, 121.16, 120.19, 107.31, 107.10, 61.99, 53.46, 53.40, 45.33, 36.28, 36.24, 30.35, 27.57, 27.47. HRMS (ESI): calcd. for C_23_H_27_BrN_4_O_2_ [M + H]^+^ 471.1395, found 471.1384.

#### 4.1.6. General procedure for the synthesis of target compounds 11a–11f

Intermediates **10a–10f** (0.3 g, 1.0 equiv.) and PyBOP (1.3 equiv.) and DIPEA(2.0 equiv.) were added to 6 ml of DMF and stirred at room temperature for 20 min. Then, intermediates **4a, 4g, 4m** (1.0 equiv.) were added and stirred at room temperature for 4 h. After completion of the reaction, the reaction mixture was quenched with water. The aqueous phase was extracted with CH_2_Cl_2_ (3 × 15 ml). The CH_2_Cl_2_ layer was then washed with brine and dried over anhydrous Na_2_SO_4_. After concentration, the crude product was purified by silica gel column chromatograph (CH_2_Cl_2_/methanol  =  80:1) to give target compounds **11a–11f**.

##### 4.1.6.1. N-((1–(4-methoxybenzyl)piperidin-4-yl)methyl)-1H-indole-5-carboxamide (11a)

White solid, yield: 67%, m.p. 89–90 °C. ^1^H NMR (500 MHz, DMSO-d_6_): *δ* 8.34 (t, *J* = 5.4 Hz, 1H), 8.12 (s, 1H), 7.62 (d, *J* = 8.5 Hz, 1H), 7.41 (s, 2H), 7.34 (d, *J* = 7.5 Hz, 3H), 7.28 (d, *J* = 6.7 Hz, 1H), 6.51 (s, 1H), 3.63 (s, 3H), 3.53–3.46 (m, 2H), 3.17 (s, 2H), 2.91 (d, *J* = 10.0 Hz, 2H), 2.14 (s, 2H), 1.70 (d, *J* = 13.2 Hz, 2H), 1.64 (s, 1H), 1.29 (d, *J* = 11.7 Hz, 3H). ^13 ^C NMR (126 MHz, DMSO-d_6_): *δ* 167.87, 137.77, 129.84, 128.71, 127.89, 127.41, 127.02, 126.11, 120.99, 120.25, 114.19, 111.25, 102.47, 61.96, 55.54, 52.90, 44.95, 35.69, 29.44. HRMS (ESI): calcd. for C_23_H_27_N_3_O_2_ [M + H]^+^ 378.2181, found 378.2179.

##### 4.1.6.2. N-((1–(4-methoxybenzyl)piperidin-4-yl)methyl)-1H-indazole-5-carboxamide (11b)

White solid, yield: 65%, m.p. 78–79 °C. ^1^H NMR (500 MHz, DMSO-d_6_): *δ* 13.33 (s, 1H), 8.53 (s, 1H), 8.34 (s, 1H), 8.19 (s, 1H), 7.85 (d, *J* = 8.7 Hz, 1H), 7.57 (d, *J* = 8.7 Hz, 1H), 7.34 (d, *J* = 7.9 Hz, 2H), 6.92 (d, *J* = 8.3 Hz, 2H), 3.75 (s, 5H), 3.19 (s, 2H), 3.00 (d, *J* = 9.1 Hz, 2H), 2.31 (s, 2H), 1.74 (d, *J* = 13.0 Hz, 2H), 1.69 (s, 1H), 1.37 (d, *J* = 11.1 Hz, 2H). ^13 ^C NMR (126 MHz, DMSO-d_6_): *δ* 167.10, 159.41, 135.14, 131.68, 127.55, 125.75, 122.76, 120.93, 114.19, 55.54, 52.24, 44.76, 27.05. HRMS (ESI): calcd. for C_22_H_26_N_4_O_2_ [M + H]^+^ 379.2134, found 379.2124.

##### 4.1.6.3. N-((1–(2-chlorobenzyl)piperidin-4-yl)methyl)-1H-indazole-5-carboxamide (11c)

White solid, yield: 78%, m.p. 89–90 °C. ^1^H NMR (500 MHz, DMSO-d_6_)：*δ* 13.27 (s, 1H), 8.34 (s, 1H), 8.20 (s, 1H), 7.86 (d, *J* = 8.6 Hz, 1H), 7.57 (d, *J* = 8.7 Hz, 1H), 7.48 (d, *J* = 7.2 Hz, 1H), 7.41 (d, *J* = 7.7 Hz, 1H), 7.32 (s, 1H), 7.27 (d, *J* = 7.4 Hz, 1H), 7.03 (s, 1H), 3.53 (s, 2H), 3.19 (d, *J* = 5.7 Hz, 2H), 2.83 (d, *J* = 10.8 Hz, 2H), 2.00 (t, *J* = 11.0 Hz, 2H), 1.68 (d, *J* = 12.1 Hz, 2H), 1.60 (s, 1H), 1.22 (d, *J* = 9.8 Hz, 2H). ^13 ^C NMR (126 MHz, DMSO-d_6_): *δ* 167.06, 136.55, 133.63, 131.07, 129.62, 128.85, 127.68, 127.41, 125.80, 122.80, 120.89, 110.11, 59.40, 53.61, 45.32, 36.16, 30.38. HRMS (ESI): calcd. for C_21_H_23_ClN_4_O [M + H]^+^ 383.1638, found 383.1629.

##### 4.1.6.4. N-((1–(4-methoxybenzyl)piperidin-4-yl)methyl)-1H-benzo[d]imidazole-5-carboxamide (11d)

White solid, yield: 76%, m.p. 92–93 °C. ^1^H NMR (500 MHz, DMSO-d_6_): *δ* 8.84 (s, 1H), 8.48 (s, 1H), 7.93 (d, *J* = 5.5 Hz, 2H), 7.36 (d, *J* = 8.0 Hz, 2H), 6.91 (d, *J* = 8.1 Hz, 2H), 3.80 (s, 3H), 3.73 (s, 2H), 3.20 (s, 2H), 3.03 (d, J = 10.6 Hz, 2H), 2.40 (s, 2H), 1.74 (s, 3H), 1.41 (d, J = 11.2 Hz, 2H). ^13 ^C NMR (126 MHz, DMSO-d_6_)：*δ* 166.67, 159.53, 140.23, 139.32, 132.00, 131.78, 129.35, 127.39, 126.11, 125.43, 115.74, 114.34, 114.20, 60.23, 55.54, 51.93, 44.74, 34.93, 28.29. HRMS (ESI): calcd. for C_22_H_26_N_4_O_2_ [M + H]^+^ 379.2134, found 379.2135.

##### 4.1.6.5. N-((1–(2-chlorobenzyl)piperidin-4-yl)methyl)-1H-benzo[d]imidazole-5-carboxamide(11e)

White solid, yield: 45%, m.p 95–97 °C. ^1^H NMR (500 MHz, DMSO-d_6_): *δ* 8.51 (t, *J* = 5.4 Hz, 1H), 8.35 (s, 1H), 8.20 (s, 1H), 7.79 (d, *J* = 8.3 Hz, 1H), 7.64 (d, *J* = 8.0 Hz, 1H), 7.46 (d, *J* = 7.3 Hz, 1H), 7.38 (d, *J* = 7.8 Hz, 1H), 7.28 (d, *J* = 7.4 Hz, 1H), 7.24 (d, *J* = 7.5 Hz, 1H), 3.50 (s, 2H), 3.20 (d, *J* = 5.9 Hz, 2H), 2.80 (d, *J* = 11.2 Hz, 2H), 1.97 (d, *J* = 10.7 Hz, 2H), 1.67 (d, *J* = 13.0 Hz, 2H), 1.61–1.54 (m, 1H), 1.22 (d, *J* = 9.6 Hz, 2H). ^13 ^C NMR (126 MHz, DMSO-d_6_): *δ* 167.33, 144.20, 136.52, 133.65, 131.04, 129.58, 129.17, 128.78, 128.51, 127.35, 121.95, 59.40, 53.59, 45.35, 36.17, 30.38. HRMS (ESI): calcd. for C_21_H_23_ClN_4_O [M + H]^+^ 383.1638, found 383.1626.

##### 4.1.6.6. N-((1-benzylpiperidin-4-yl)methyl)-1H-benzo[d][1,2,3]triazole-5-carboxamide(11f)

White solid, yield: 89%, m.p. 101–103 °C. ^1^H NMR (500 MHz, DMSO-d_6_): δ 8.63 (t, *J* = 5.5 Hz, 1H), 8.44 (s, 1H), 7.87 (d, *J* = 2.7 Hz, 2H), 7.47 (d, *J* = 7.1 Hz, 1H), 7.39 (d, *J* = 7.8 Hz, 1H), 7.30 (d, *J* = 6.9 Hz, 1H), 7.28 – 7.23 (m, 1H), 7.03 (s, 1H), 3.52 (s, 2H), 3.21 (t, *J* = 6.1 Hz, 2H), 3.18 (s, 1H), 2.82 (d, *J* = 11.3 Hz, 2H), 2.00 (t, *J* = 10.4 Hz, 2H), 1.68 (d, *J* = 12.0 Hz, 2H), 1.61 (s, 1H), 1.27 – 1.14 (m, 2H). ^13 ^C NMR (126 MHz, DMSO-d_6_): *δ* 166.83, 141.15, 140.74, 136.52, 133.63, 131.07, 129.60, 128.83, 127.39, 124.43, 115.72, 114.53, 59.39, 53.58, 45.41, 36.12, 30.36. HRMS (ESI): calcd. for C_20_H_23_N_5_O [M + H]^+^ 350.1981, found 350.1980.

#### 4.1.7. General procedure for the synthesis of intermediate 13

To a stirred solution of 1,3-dihydro-2*H*-benzo[*d*]imidazol-2-one (**12**) (2.0 g, 14.9 mmol, 1.0 equiv.) in DMF (20 ml) at 0 °C, NaH (60% dispersion in mineral oil, 1.5 g, 37.3 mmol, 2.5 equiv.) was added, and the mixture was stirred for 15 min. Methyl iodide (5.3 g, 37.3 mmol, 2.5 equiv.) was added dropwise, and the mixture was stirred at room temperature for an additional 12 h. Water (30 ml) was added and extracted with EtOAc (30 ml × 3). The combined organic layers were dried over anhydrous Na_2_SO_4_, filtered, and concentrated *in vacuo*. The crude residue was purified by silica gel chromatography (petroleum ether/EtOAc  =  8:1) to give intermediate **13** (2.0 g, 83%) as a white solid^24^.

#### 4.1.8. General procedure for the synthesis of intermediate 14

Chlorosulfonic acid (3.0 ml) was added slowly to intermediate **13** (1.0 g, 6.2 mmol) at room temperature, and the reaction mixture was stirred for 18 h. The resulting mixture was added to the ice with NaCl(s) carefully, filtered, and washed with ether to collect the desired product, which was used directly to the next step (1.4 g, 87%)^26^.

#### 4.1.9. General procedure for the synthesis of target compounds 15a–15j

A solution of appropriate intermediate **14** (0.3 g 1.2 mmol, 1.0 equiv.) in dichloromethane (15 ml) was slowly added to a cooled (0 °C) solution of intermediates **4a–4d, 4f, 4i, 4l–4o** (1.0 equiv.) in dichloromethane (10 ml) and triethylamine (2.0 equiv.). The resulting mixture was stirred at room temperature overnight. After concentration, the crude product was purified by silica gel column chromatograph (CH_2_Cl_2_/methanol  =  50:1) to give target compounds **15a–15j**^27^.

##### 4.1.9.1. N-((1-benzylpiperidin-4-yl)methyl)-1,3-dimethyl-2-oxo-2,3-dihydro-1H-benzo[d]imidazole-5-sulphonamide (15a)

White solid, yield: 64%, m.p. 113–114 °C. ^1^H NMR (500 MHz, CDCl_3_): *δ* 7.65 (dd, *J_1_ = * 8.2, *J_2_ = * 1.7 Hz, 1H), 7.49 (d, *J* = 1.5 Hz, 1H), 7.34 – 7.29 (m, 5H), 7.07 (d, *J* = 8.2 Hz, 1H), 4.43 (t, *J* = 6.2 Hz, 1H), 3.49 (d, *J* = 2.3 Hz, 6H), 2.88 (d, *J* = 11.7 Hz, 2H), 2.83 (t, *J* = 6.7 Hz, 2H), 1.92 (t, *J* = 11.0 Hz, 2H), 1.64 (s, 6H), 1.47 (m, 1H). ^13 ^C NMR (126 MHz, DMSO-d_6_): *δ* 154.54, 133.39, 133.02, 130.05, 129.19, 128.56, 127.26, 120.54, 107.96, 106.37, 62.79, 53.23, 48.56, 36.08, 29.94, 27.70, 27.64. HRMS (ESI): calcd. for C_22_H_28_N_4_O_3_S [M + H]^+^ 429.1960, found 429.1965.

###### 4.1.9.2. 1,3-dimethyl-N-((1–(2-methylbenzyl)piperidin-4-yl)methyl)-2-oxo-2,3-dihydro-1H-benzo[d]imidazole-5-sulphonamide (15b)

White solid, yield: 73%, m.p. 68–70 °C. ^1^H NMR (500 MHz, CDCl_3_): δ 7.67 (s, 1H), 7.51 (s, 1H), 7.17 (d, *J* = 11.8 Hz, 3H), 7.08 (d, *J* = 6.8 Hz, 1H), 4.59 (s, 1H), 3.49 (s, 6H), 3.13 (d, *J* = 7.3 Hz, 2H), 2.92 (s, 2H), 2.84 (t, *J* = 6.5 Hz, 2H), 2.35 (s, 3H), 2.01 (s, 2H), 1.67 (s, 2H), 1.43 (t, *J* = 7.3 Hz, 2H), 1.27 (s, 2H). ^13 ^C NMR (126 MHz, DMSO-d_6_): *δ* 154.52, 137.65, 133.42, 133.01, 130.56, 130.05, 127.69, 125.88, 120.56, 107.90, 106.36, 60.25, 53.13, 48.38, 35.79, 29.50, 27.66, 27.61, 19.36. HRMS (ESI): calcd. for C_23_H_30_N_4_O_3_S [M + H]^+^ 443.2117, found 443.2111.

###### 4.1.9.3. 1,3-dimethyl-N-((1–(3-methylbenzyl)piperidin-4-yl)methyl)-2-oxo-2,3-dihydro-1H-benzo[d]imidazole-5-sulphonamide (15c)

White solid, yield: 58%, m.p. 79–80 °C. ^1^H NMR (500 MHz, CDCl_3_): *δ* 7.65 (dd, *J_1_ = * 8.2, *J_2_ = * 1.7 Hz, 1H), 7.49 (d, *J* = 1.6 Hz, 1H), 7.21 (dd, *J_1_* = 9.8, *J_2_* = 5.2 Hz, 1H), 7.11 (s, 1H), 7.07 (t, *J* = 7.7 Hz, 3H), 4.45 (t, *J* = 6.4 Hz, 1H), 3.49 (d, *J* = 2.5 Hz, 6H), 3.44 (s, 2H), 2.87 (d, *J* = 11.6 Hz, 2H), 2.83 (t, *J* = 6.7 Hz, 2H), 2.35 (s, 3H), 1.90 (dd, *J_1_* = 11.7, *J_2_* = 9.8 Hz, 2H), 1.65 (s, 4H), 1.52–1.42 (m, 1H). ^13 ^C NMR (126 MHz, DMSO-d_6_): *δ* 154.55, 137.55, 133.45, 133.03, 130.07, 129.78, 128.42, 127.87, 126.27, 120.54, 107.93, 106.36, 62.86, 53.29, 48.58, 36.11, 29.97, 27.69, 27.63, 21.46. HRMS (ESI): calcd. for C_23_H_30_N_4_O_3_S [M + H]^+^ 443.2117, found 443.2119.

###### 4.1.9.4. 1,3-dimethyl-N-((1–(4-methylbenzyl)piperidin-4-yl)methyl)-2-oxo-2,3-dihydro-1H-benzo[d]imidazole-5-sulphonamide (15d)

White solid, yield: 77%, m.p. 150–152 °C. ^1^H NMR (500 MHz, CDCl_3_) δ 7.65 (dd, *J_1_* = 8.2, *J_2_ = * 1.5 Hz, 1H), 7.49 (d, *J* = 1.2 Hz, 1H), 7.20 (d, *J* = 8.5 Hz, 1H), 7.17 (d, *J* = 8.2 Hz, 1H), 7.13 (s, 1H), 7.06 (d, *J* = 8.2 Hz, 1H), 6.85 (d, *J* = 8.5 Hz, 1H), 4.58 (d, *J* = 5.9 Hz, 1H), 3.81 (s, 2H), 3.48 (d, *J* = 4.4 Hz, 6H), 3.43 (d, *J* = 10.7 Hz, 2H), 2.85 (d, *J* = 11.5 Hz, 2H), 2.81 (d, *J* = 6.6 Hz, 2H), 1.88 (t, *J* = 11.4 Hz, 2H), 1.64 (d, *J* = 12.3 Hz, 2H), 1.50 – 1.40 (m, 1H), 1.27 (s, 3H). ^13 ^C NMR (126 MHz, DMSO-d_6_): *δ* 158.62, 154.55, 133.42, 133.03, 130.37, 130.06, 129.11, 120.54, 113.92, 107.95, 106.37, 62.21, 55.43, 53.11, 48.58, 36.15, 29.98, 27.70, 27.64, 21.15. HRMS (ESI): calcd. for C_23_H_30_N_4_O_3_S [M + H]^+^ 443.2117, found 443.2111.

##### 4.1.9.5. N-((1–(3-methoxybenzyl)piperidin-4-yl)methyl)-1,3-dimethyl-2-oxo-2,3-dihydro-1H-benzo[d]imidazole-5-sulphonamide (15e)

White solid, yield: 55%, m.p. 80–81 °C. ^1^H NMR (500 MHz, CDCl_3_): *δ* 7.66 (dd, *J_1_* = 8.2, *J_2_* = 1.7 Hz, 1H), 7.49 (d, *J* = 1.5 Hz, 1H), 7.23 (t, *J* = 7.9 Hz, 1H), 7.07 (d, *J* = 8.2 Hz, 1H), 6.90 – 6.85 (m, 2H), 6.83 – 6.78 (m, 1H), 4.47 (t, *J* = 6.4 Hz, 1H), 3.82 (s, 3H), 3.49 (d, *J* = 2.5 Hz, 6H), 3.46 (s, 2H), 2.87 (d, *J* = 11.6 Hz, 2H), 2.83 (t, *J* = 6.7 Hz, 2H), 1.91 (dd, *J_1_* = 11.6, *J_2_* = 9.9 Hz, 2H), 1.65 (d, *J* = 16.5 Hz, 4H), 1.50 – 1.43 (m, 1H). ^13 ^C NMR (126 MHz, DMSO-d_6_): *δ* 159.66, 154.55, 140.79, 133.45, 133.03, 130.07, 129.55, 121.31, 120.54, 114.55, 112.60, 107.93, 106.36, 62.73, 55.37, 53.27, 48.59, 36.10, 29.99, 27.69, 27.63. HRMS (ESI): calcd. for C_23_H_30_N_4_O_4_S [M + H]^+^ 459.2066, found 459.2068.

##### 4.1.9.6. N-((1–(3-fluorobenzyl)piperidin-4-yl)methyl)-1,3-dimethyl-2-oxo-2,3-dihydro-1H-benzo[d]imidazole-5-sulphonamide (15f)

White solid, yield: 74%, m.p. 75–76 °C. ^1^H NMR (500 MHz, CDCl_3_): *δ* 7.66 (dd, *J_1_* = 8.2, *J_2_* = 1.6 Hz, 1H), 7.49 (d, *J* = 1.5 Hz, 1H), 7.25 (d, *J* = 7.9 Hz, 1H), 7.06 (dd, *J_1_* = 15.3, *J_2_* = 8.8 Hz, 3H), 6.97 – 6.90 (m, 1H), 4.41 (t, *J* = 6.4 Hz, 1H), 3.49 (d, *J* = 1.7 Hz, 6H), 3.47 (s, 2H), 2.86 (d, *J* = 6.8 Hz, 2H), 2.83 (d, *J* = 6.7 Hz, 2H), 1.93 (t, *J* = 10.7 Hz, 2H), 1.69 – 1.59 (m, 4H), 1.53 – 1.44 (m, 1H). ^13 ^C NMR (126 MHz, DMSO-d_6_): *δ* 163.63, 161.70, 154.53, 142.36, 133.44, 133.02, 130.44, 130.37, 130.06, 124.99, 120.54, 115.51, 115.34, 114.05, 113.88, 107.91, 106.34, 62.02, 53.21, 48.56, 36.04, 29.97, 27.67, 27.61. HRMS (ESI): calcd. for C_22_H_27_FN_4_O_3_S [M + H]^+^ 447.1866, found 447.1869.

##### 4.1.9.7. N-((1–(3-chlorobenzyl)piperidin-4-yl)methyl)-1,3-dimethyl-2-oxo-2,3-dihydro-1H-benzo[d]imidazole-5-sulphonamide (15g)

White solid, yield: 70%, m.p. 73–75 °C. ^1^H NMR (500 MHz, CDCl_3_): *δ* 7.66 (dd, *J_1_* = 8.2, *J_2_* = 1.6 Hz, 1H), 7.49 (d, *J* = 1.4 Hz, 1H), 7.31 (s, 1H), 7.25 – 7.21 (m, 2H), 7.19 – 7.16 (m, 1H), 7.07 (d, *J* = 8.2 Hz, 1H), 4.45 (t, *J* = 6.5 Hz, 1H), 3.49 (d, *J* = 1.8 Hz, 6H), 3.44 (s, 2H), 2.85 (d, *J* = 3.3 Hz, 2H), 2.83 (d, *J* = 6.5 Hz, 2H), 1.92 (t, *J* = 10.7 Hz, 2H), 1.63 (s, 4H), 1.52 – 1.43 (m, 1H). ^13 ^C NMR (126 MHz, DMSO-d_6_): *δ* 154.55, 141.91, 133.46, 133.33, 133.03, 130.42, 130.07, 128.68, 127.69, 127.19, 120.54, 107.92, 106.35, 61.93, 53.20, 48.55, 36.03, 29.97, 27.69, 27.63. HRMS (ESI): calcd. for C_22_H_27_ClN_4_O_3_S [M + H]^+^ 463.1570, found 463.1568.

##### 4.1.9.8. N-((1–(4-chlorobenzyl)piperidin-4-yl)methyl)-1,3-dimethyl-2-oxo-2,3-dihydro-1H-benzo[d]imidazole-5-sulphonamide (15h)

White solid, yield: 71%, m.p. 195–197 °C. ^1^H NMR (500 MHz, CDCl_3_): *δ* 7.66 (d, *J* = 7.5 Hz, 1H), 7.51 (s, 1H), 7.39 – 7.30 (m, 4H), 7.07 (d, *J* = 8.2 Hz, 1H), 4.55 (s, 1H), 3.49 (s, 6H), 2.88 (s, 2H), 2.83 (t, *J* = 6.7 Hz, 2H), 1.96 (s, 2H), 1.76 – 1.63 (m, 4H), 1.48 (d, *J* = 32.9 Hz, 2H), 1.43 (t, *J* = 7.3 Hz, 1H). ^13 ^C NMR (126 MHz, DMSO-d_6_): *δ* 154.55, 133.44, 133.04, 130.98, 130.07, 128.58, 120.54, 107.94, 106.36, 61.20, 53.08, 48.48, 46.03, 35.94, 29.67, 27.70, 27.64. HRMS (ESI): calcd. for C_22_H_27_ClN_4_O_3_S [M + H]^+^ 463.1570, found 463.1565.

##### 4.1.9.9. N-((1–(2-bromobenzyl)piperidin-4-yl)methyl)-1,3-dimethyl-2-oxo-2,3-dihydro-1H-benzo[d]imidazole-5-sulphonamide (15i)

White solid, yield: 81%, m.p. 134–135 °C. ^1^H NMR (500 MHz, CDCl_3_): *δ* 7.66 (dd, *J_1_ = * 8.2, *J_2_ = * 1.7 Hz, 1H), 7.54 (dd, *J_1_* = 8.0, *J_2_ = * 0.9 Hz, 1H), 7.50 (d, *J* = 1.5 Hz, 1H), 7.46 (d, *J* = 7.3 Hz, 1H), 7.30 (s, 1H), 7.15 – 7.10 (m, 1H), 7.07 (d, *J* = 8.2 Hz, 1H), 4.55 (t, *J* = 6.3 Hz, 1H), 3.59 (s, 2H), 3.49 (d, *J* = 1.8 Hz, 6H), 2.91 (d, *J* = 11.1 Hz, 2H), 2.84 (t, *J* = 6.7 Hz, 2H), 2.07 (t, *J* = 11.3 Hz, 2H), 1.68 (d, J = 12.7 Hz, 2H), 1.56 – 1.49 (m, 1H), 1.27 (s, 2H). ^13 ^C NMR (126 MHz, DMSO-d_6_): *δ* 154.52, 138.08, 133.45, 133.02, 132.89, 131.15, 130.06, 129.17, 127.96, 124.32, 120.55, 107.91, 106.35, 61.83, 53.35, 48.52, 46.16, 35.99, 30.00, 27.68, 27.63. HRMS (ESI): calcd. for C_22_H_27_BrN_4_O_3_S [M + H]^+^ 507.1065, found 507.1017.

##### 4.1.9.10. N-((1–(3-bromobenzyl)piperidin-4-yl)methyl)-1,3-dimethyl-2-oxo-2,3-dihydro-1H-benzo[d]imidazole-5-sulphonamide (15j)

White solid, yield: 75%, m.p. 175–177 °C. ^1^H NMR (500 MHz, CDCl_3_): *δ* 7.67 (d, *J* = 8.0 Hz, 1H), 7.50 (d, *J* = 8.1 Hz, 2H), 7.39 (dd, *J_1_* = 20.8, *J_2_* = 7.7 Hz, 2H), 7.20 (t, *J* = 7.8 Hz, 1H), 7.07 (d, *J* = 8.2 Hz, 1H), 4.51 (s, 1H), 3.49 (s, 6H), 3.15 (d, *J* = 7.3 Hz, 2H), 2.89 (s, 2H), 2.84 (t, *J* = 6.7 Hz, 2H), 2.00 (s, 2H), 1.72 – 1.63 (m, 2H), 1.46 (t, *J* = 7.3 Hz, 2H), 1.27 (s, 1H). ^13 ^C NMR (126 MHz, DMSO-d_6_): *δ* 154.52, 133.40, 133.01, 130.81, 130.05, 128.74, 122.04, 120.55, 107.92, 106.37, 61.20, 52.91, 48.36, 46.03, 35.68, 29.67, 27.69, 27.64. HRMS (ESI): calcd. for C_22_H_27_BrN_4_O_3_S [M + H]^+^ 507.1065, found 507.1060.

### 4.2. AChE and BChE inhibition assay

The inhibitory activities of the target compounds towards AChE (from *Electrophorus electricus* (electric *ee*AChE, Sigma-Aldrich) and *horse serum* BChE (*eq*BChE, Sigma-Aldrich) or human AChE – huAChE; human BuChE – huBuChE (huAChE, huBuChE, Sigma-Aldrich) were measured by Ellman’s method^20^. In 96-well plates, a mixture of phosphate buffer (0.1 M, pH 8.0, 2 ml), 5,5′-dithiobis-2-nitrobenzoic acid (DTNB, 60 µL), acetylcholinestrase or butyrylcholinesterase (20 µL, 5 IU/mL) and different concentration of the compound solution (30 µL) was pre-incubated for 5 min followed by the addition of the substrate (acetylthiocholine iodide or butyrylthiocholine iodide, 20 µL). Changes in absorbance were measured at 412 nm by using microplate reader (Thermo, Varioskan Flash 3001, USA). The measurement of each concentration for each compound was detected in triplicate. GraphPad Prism 6.0 (GraphPad Software, Inc., La Jolla, CA) was used for data processing. The inhibition curve was fitted by plotting percentage enzyme activity (100% for the reference) versus the logarithm of the concentration of tested compound. The half-maximal inhibitory concentration (IC_50_) values were calculated according to the inhibition curve and the data were shown in the layout of mean ± SEM by GraphPad Prism 6.0.

### 4.3. Kinetic studies of AChE and BChE inhibition

Kinetic studies were performed in the same manner as the determination of ChEs inhibition, while the substrate (ATC/BTC) was used in concentrations of 90, 150, 226, 452, and 904 µM. The concentrations of test compounds were set to 0, 2, 5, 10, and 20 µM for **15b** and 0, 0.5, 1, 2, and 5 µM for **15j**. The enzymatic reaction was extended to 7 min for *ee*AChE and *eq*BuChE before the determination of the absorption. Vmax and *K*_m_ values of the Michaelis–Menten kinetics were calculated by nonlinear regression from substrate-velocity curves using GraphPad Prism 6.0. Linear regression was used for fitting the Lineweaver–Burk plots.

### 4.4. Molecular docking study

The crystal structures of the hAChE (PDB ID: 4EY7)^28^ and hBChE (PDB ID: 4TPK)^29^ were derived from the RCSB Protein Data Bank. Docking studies were carried out using the Discovery Studio 2016 for compound **15b** and compound **15j**. Two protein structures were preprocessed (i.e. protonated, removed water, added Miss sidechains, etc.) by “prepare protein” module in DS to give the structures suitable for docking. “Prepare ligands” module in DS was applied for the structural preparation of the test compounds. The native ligand in the crystal structure was used to define the binding site. The binding site was defined as a site sphere (in 10 Å radius) around the original ligands in the co-crystal structures. The docking programme CDOCKER encoded in DS 2016 was applied to identify the potential binding of compound **15b** to the hAChE and compound **15j** to the hBChE. Other CDOCKER parameters were set to default values. Compound **15b** created 10 poses to the hAChE and compound **15j** created 10 poses to the hBuChE. the poses were visually inspected, and the most suitable docking pose was selected on the basis of the score and interactions with key residues of the active site of hAChE and hBChE.

### 4.5. Cell studies *in vitro*

PC12 cell and HepG2 cell (human hepatocellular liver carcinoma cell line purchased from Institute of Materia of Chinese Academy of Medical Science), were grown in DMEM supplemented with 10% FBS at 37 °C in a humidified atmosphere containing 5% CO_2_. For the experiments, cells (6 × 10^3^ cells/well) were seeded in 96-well plate in complete medium. After 24 h, the medium was removed, and cells were exposed to the increasing concentrations of compounds **15b** and **15j** or donepezil (10, 20, 30, and 50 µM or 5, 15, and 50 µM) in DMEM for further 24 h. Cell survival was measured through MTT assay.

### 4.6. Behavioural studies

Behavioural studies were performed by using adult male ICR mice (8–10 weeks old, weight 20–25 g), which were purchased from the Yangzhou University Medical Centre (Yangzhou, China). Scopolamine hydrobromide was supplied by Aladdin Reagents (H1507073, Shanghai, China). Tacrine was synthesised in our lab with >95% purity as determined by HPLC.

The mice were separated into five groups as follows: (i) vehicle as blank control, (ii) scopolamine as model group, (iii) tacrine as positive control, (iv) compound **15b** as test group, and (v) compound **15j** as test group. Tacrine, **15b** and **15j** (20 mmol kg^−1^ body weight) were orally administered to mice in groups (iii), (iv), and (v), respectively, 30 min before the ip administration of scopolamine (1 mg kg^−1^) or saline for 10 consecutive days.

Cognitive function was evaluated by the Morris water maze analysis-management system (SMART 3.0, Panlab, Madrid, Spain), according to the method previously described^30^.The maze was placed in a lit room with visual cues at 25 °C. An escape platform (10 cm diameter) was located in the centre of one quadrant of the circular pool (120 cm diameter, 60 cm height) with a depth of 40 cm water. The behavioural study of each mouse included 5 days of learning and memory training and a probe trial on day 6. The animal starting positions faced to the pool wall, and were pseudorandomised for each trial. For the cognitive evaluation, each mouse was individually evaluated on both visible-platform (days 1–2) and hidden-platform (days 3–5) versions of the water maze. All mice received nonspatial pretraining during the first two training days, which prepared them for the subsequent spatial learning test. During the two days, mice were trained to find the platform that was labelled by a small flag (5 cm tall). The hidden platform version was used to determine the retention of memory to find the platform. During the hidden-platform training trials, the escape platform was placed 1 cm below the surface of the water. On each day, the animal was subjected to two trials, each of which lasted for 90 s. The time for the mouse to find the platform (a successful escape) was recorded. If a mouse failed to reach the platform within 90 s, the test was terminated and the animal was gently navigated to the platform by hand. Whether a mouse was successful or failed to reach the platform within 90 s, it was kept on the platform for 30 s. On the last day (day 6), the platform was removed from its location and the animals were given a probe trial in which they had 90 s to search for the platform. The time taken to reach the missing platform and the number of times the animals crossed the platform location were recorded.

Data for the time of escape latency, the trajectory travelled, and the number of platform location crossings were recorded by SMART 3.0, Panlab, Madrid, Spain and processed by GraphPad Prism 6.0.

## Supplementary Material

Supplemental Material
