# Supplementary material for: Design, synthesis, in vitro and in vivo evaluation of benzylpiperidine-linked 1,3-dimethylbenzimidazolinones as cholinesterase inhibitors against Alzheimer’s disease
Source: J Enzyme Inhib Med Chem. 2019 Dec 20;35(1):330–43. doi: 10.1080/14756366.2019.1699553 (PMC6968383; doi:10.1080/14756366.2019.1699553)
Supplement: Supplemental Material [file IENZ_A_1699553_SM1650.pdf]

Supporting Information

Design, synthesis, *in vitro* and *in vivo* evaluation of benzylpiperidine-Linked 1,3-dimethylbenzimidazolinones as cholinesterase inhibitors against Alzheimer's disease

Jun Mo [a, 1], Tingkai Chen [d, 1], Hongyu Yang [a], Yan Guo [e], Qi Li [a], Yuting Qiao [a] Hongzhi Lin [a], Feng Feng [c, d], Wenyuan Liu [a], Yao Chen [b, *], Zongliang Liu [e, *], Haopeng Sun [a, c, *]

[a] *School of Pharmacy, China Pharmaceutical University, Nanjing, 211198, People's Republic of China*
[b] *School of Pharmacy, Nanjing University of Chinese Medicine, Nanjing, 210023, People's Republic of China*
[c] *Jiangsu Food and Pharmaceutical Science College, Huaian, 223003, People's Republic of China*
[d] *Department of Natural Medicinal Chemistry, China Pharmaceutical University, Nanjing, 211198, People's Republic of China*
[e] *School of pharmacy, Yantai University, Yantai, 264005, China*

Corresponding Authors: clarissa0710@163.com (Y. Chen); lzl_0_0@126.com (Z. L. Liu); sunhaopeng@163.com (H. P. Sun)

[1] These two authors contribute equally to this manuscript.

Corresponding Authors: clarissa0710@163.com (Y. Chen); sunhaopeng@163.com (H. P. Sun)

1. **Experimental section**

1.1. Inhibition of self-induced $A\beta_{1-42}$ aggregation

Inhibitory effect of the compounds on self-induced $A\beta_{1-42}$ aggregation was determined through a thioflavin T (ThT)-(T3516, Sigma-Aldrich) binding assay [30]. Firstly, aliquots of 2 μL of $A\beta_{1-42}$ (AS-64129–05 Anaspec Inc.) containing 2 mg/mL HFIP (1,1,1,3,3,3-hexafluoro-2-propanol, 52517 (Sigma-Aldrich) were stocked in DMSO). Then, they were diluted with 0.215M sodium phosphate buffer (pH 8.0) to the final concentration of 500 μM. Test compounds were dissolved in DMSO and then prepared at a concentration of 50 μM by the buffer. Resveratrol was used as a positive control. The $A\beta_{1-42}$ and the test sample solutions were incubated in a 96-well plate for 24 h at the room temperature. After the incubation, the tested compounds were diluted to a final volume of 150 μL with 50mM glycine-NaOH buffer (pH 8.5) containing 5mM ThT. Fluorescence intensity was read (excitation wavelength 450 nm, emission wavelength 485 nm) on a microplate reader (Thermo, Varioskan Flash 3001, USA).

The calculation of the inhibitory rate of $A\beta_{1-42}$ self-induced aggregation was performed using the following equation: $(1-IF_i/IF_c) \times 100$ %. IF_i and IFc were the fluorescence intensities measured in the presence and absence of inhibitors, respectively, after subtracting the background fluorescence of the 5mM ThT solution. Each compound was measured in triplicate.

1.2. Antioxidant activity (DPPH assay)

2,2-Diphenyl-1-picrylhydrazyl, a stable free radical with purple color, pairs with antioxidants and forms yellow colored diphenylpicrylhydrazine. The assay measures free radical scavenging capacity of compound as a result of reduction of the DPPH by antioxidant. The assay was performed by previously mentioned protocol [31-32]. Briefly, 95 mL of DPPH radical solution (100 μM) was added in a 96-well plate containing 5mL of different concentrations of test compounds dissolved in DMSO, and incubated for 30 min at 37 °C in the dark. The absorbance of each well was measured at 517 nm using a microplate reader (Thermo, Varioskan Flash 3001, USA).

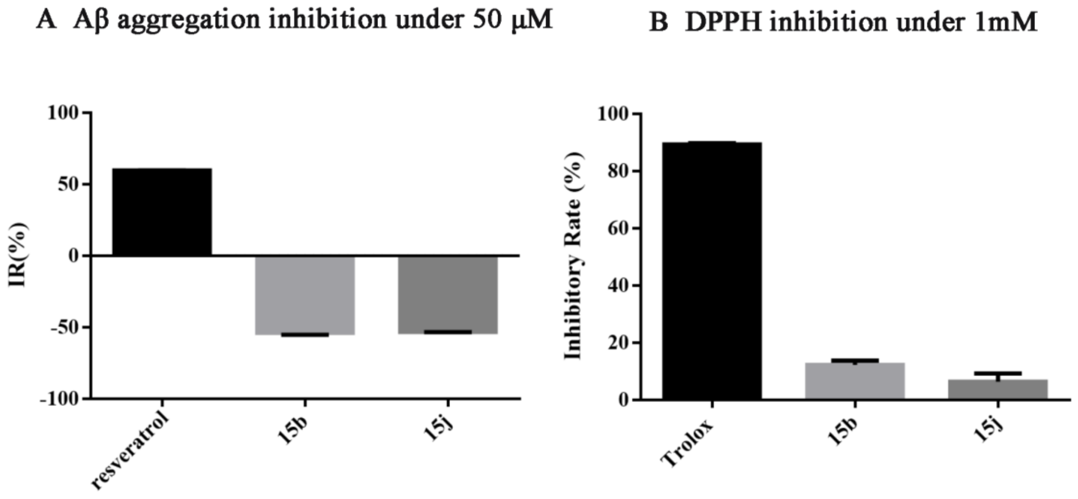

Figure S1. (A) The inhibitory effect of **15b** and **15j** on i $A\beta_{1-42}$ self-aggregation. (B) Anti-oxidant effect of compound **15b** and **15j** in DPPH assay.

1.3. Neuroprotection assay against H_2O_2-induced cell death in PC12 cells

Cytotoxicity was determined by using 3-(4,5-dimethylthiazol-2-yl)-2,5-diphenyltetrazoliumbromide (MTT) assay. The PC-12 cell line was purchased from Cell Bank of the Chinese Academy of Sciences (Shanghai, China). MTT was purchased from Sigma (M2128, St. Louis, MO). It was dissolved in phosphate buffered saline (PBS) to a stock concentration of 5 mg mL^{-1} and stored at -20 °C. PC-12 cells were plated in 96-well plates, raised to a population of 1×10^4 cells per well, and incubated overnight. After cells were treated with density gradient of test compounds or DMSO for 24 h at 37 °C or treated with 800 μM H_2O_2 for another 12 h, 20.0 μL of MTT solution was added into each well of the plate and incubated for 4 h. Then the solution was removed and 150.0 μL of DMSO was added into each well to dissolve the MTT formazan crystals. DMSO was used as a negative control. The absorbance values (OD value) were read at 570 nm by microplate reader (Thermo, Varioskan Flash 3001, USA). The inhibitory rate for each concentration of the test compound was calculated by the equation as follows:

$$IR = [1-(OD_{test}-OD_{blank})/(OD_{control}-OD_{blank})] \times 100\%$$

Here, OD_{test}, OD_{blank}, and $OD_{control}$ stand for the OD value from test compound, background, and DMSO, respectively. The IC_{50} values were calculated by GraphPad

Prism 6.0 and the data were shown in mean ± SEM.

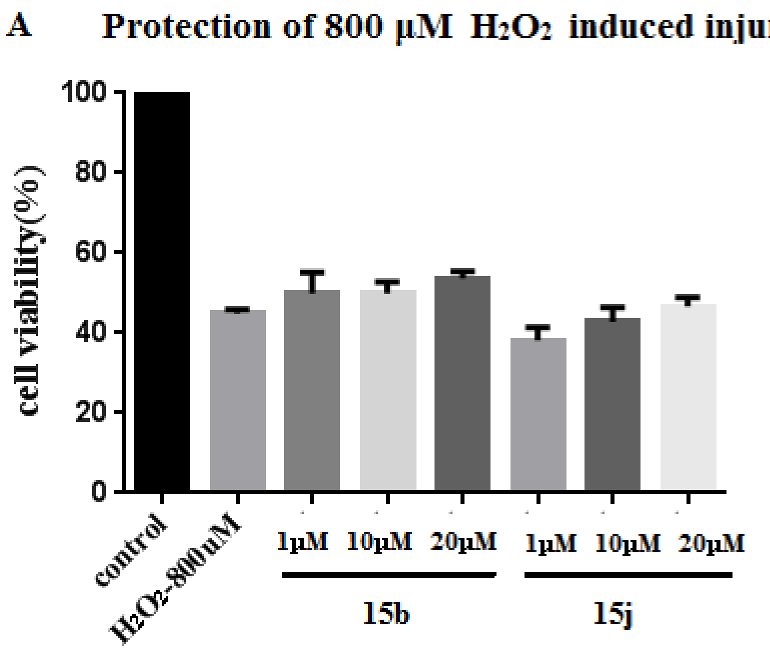

Figure S2. Protective effects of compounds **15b** and **15j** against H_2O_2-induced cell death in PC-12 cells.

2. The ^{1}H NMR , ^{13}C NMR and HRMS (ESI) spectrum of target compounds

9a. ^{1}H NMR

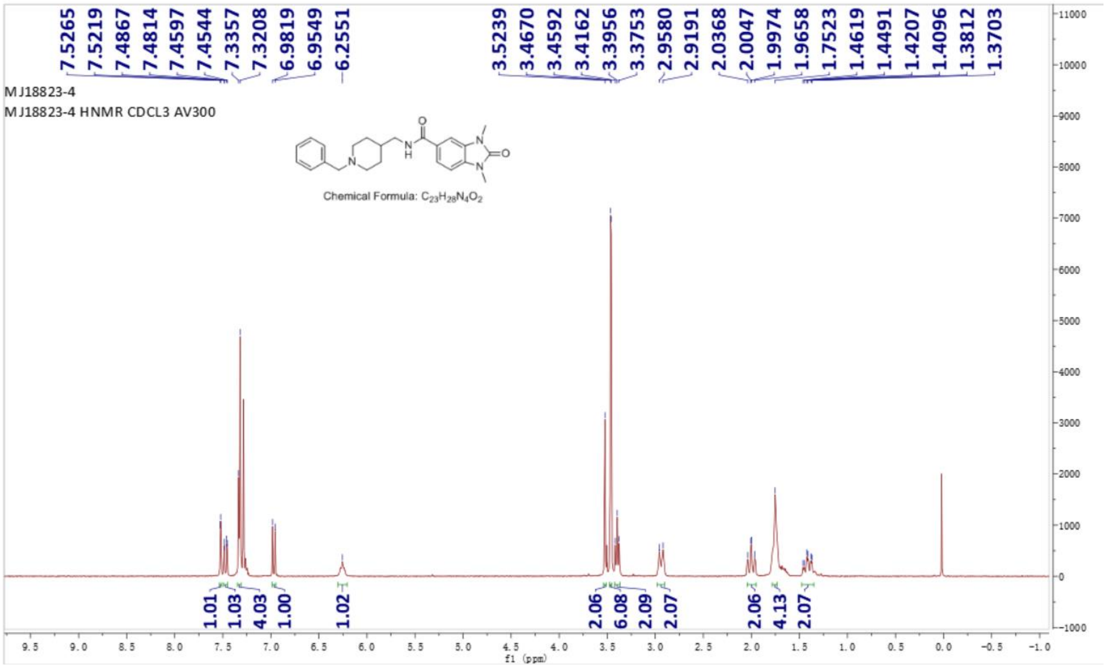

9a. ^{13}C NMR

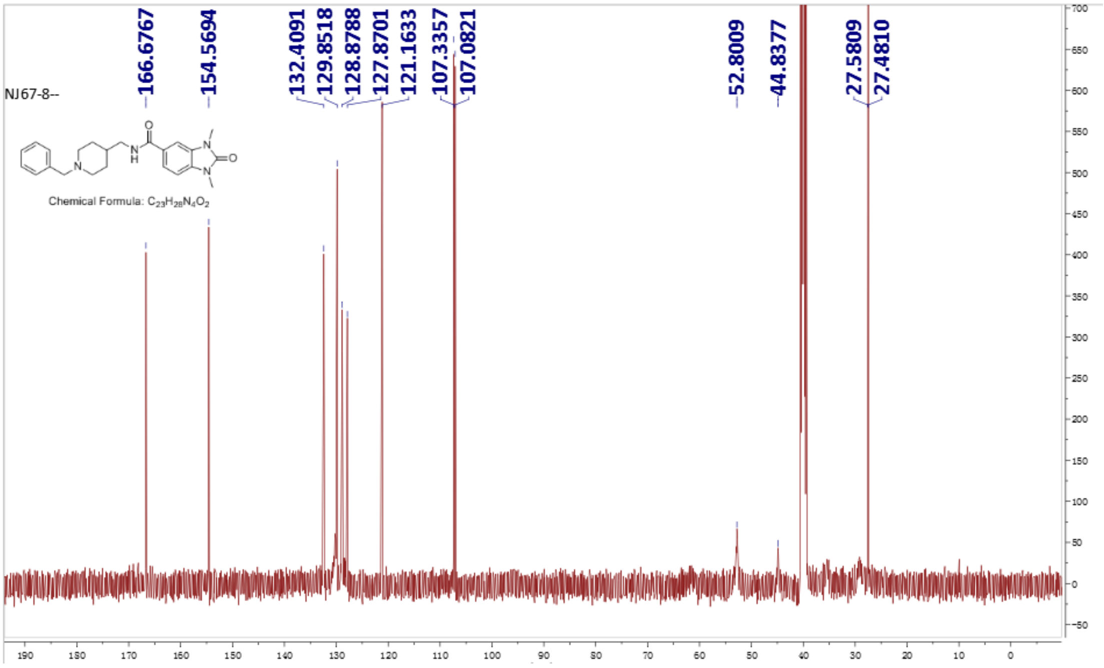

9a. HRMS (ESI)

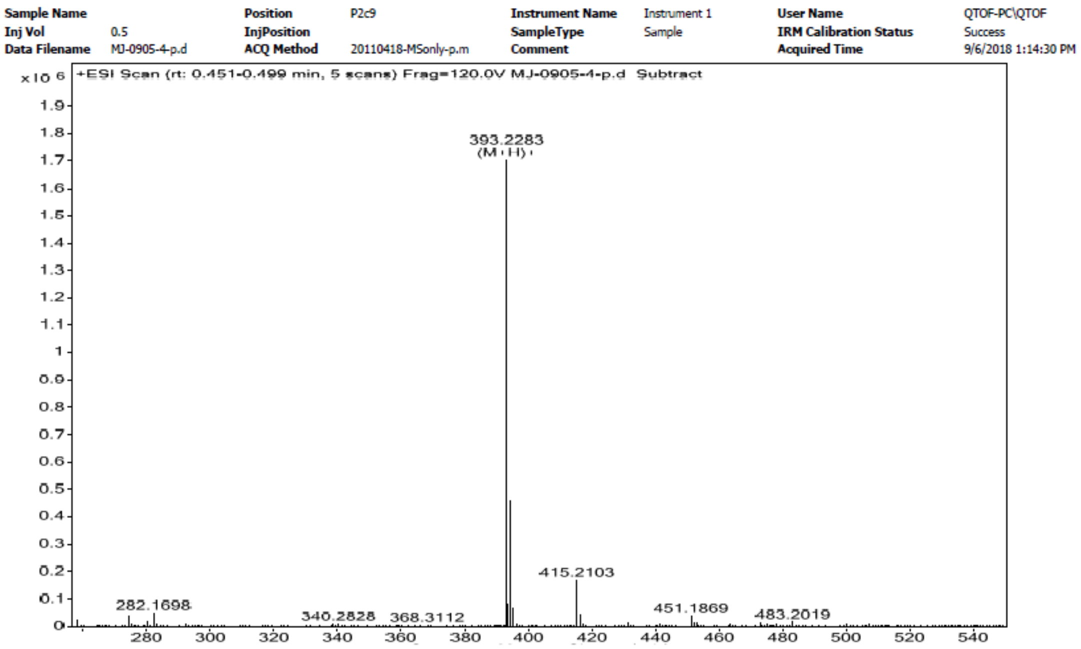

9b. ^{1}H NMR

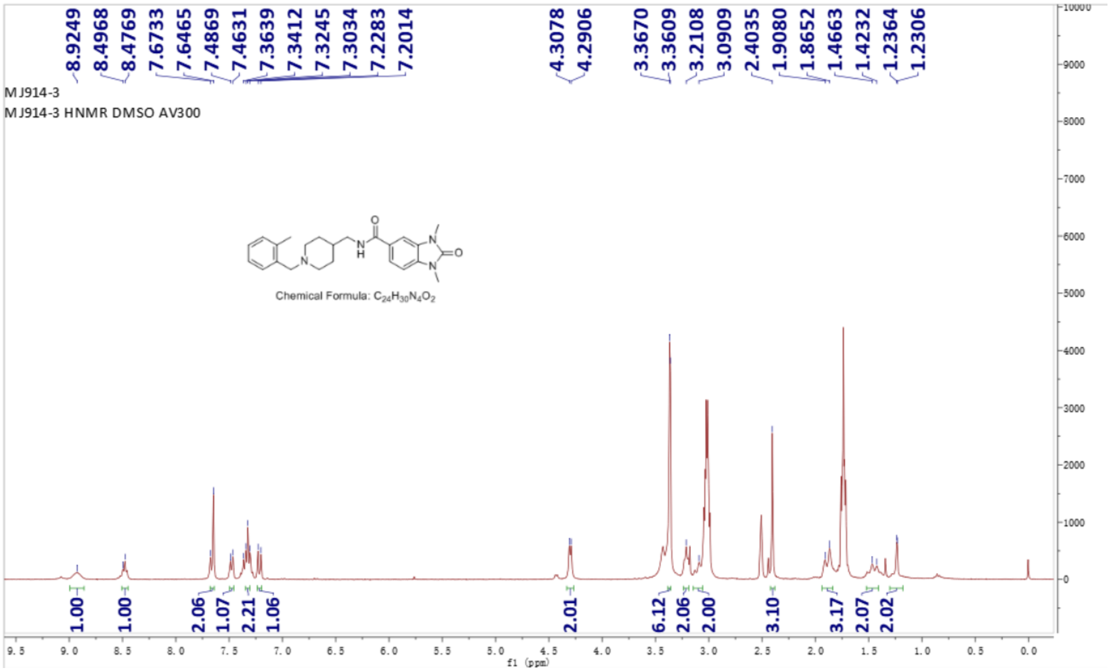

9b. ^{13}C NMR

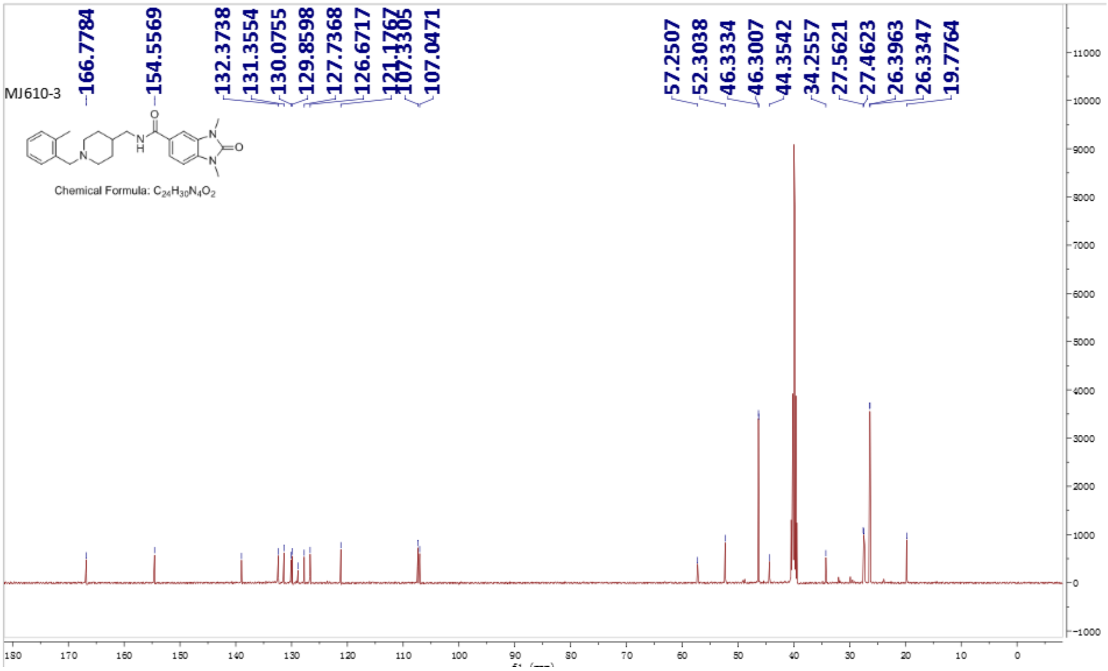

9b. HRMS (ESI)

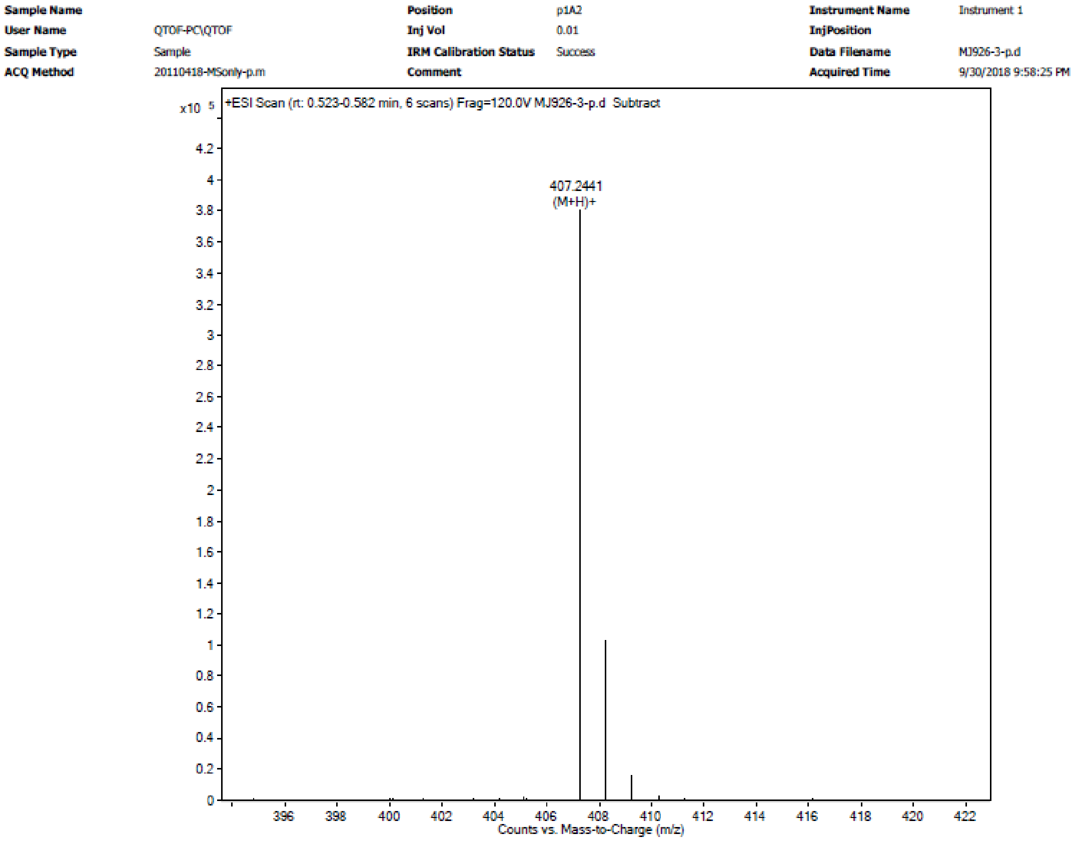

9c. ^{1}H NMR

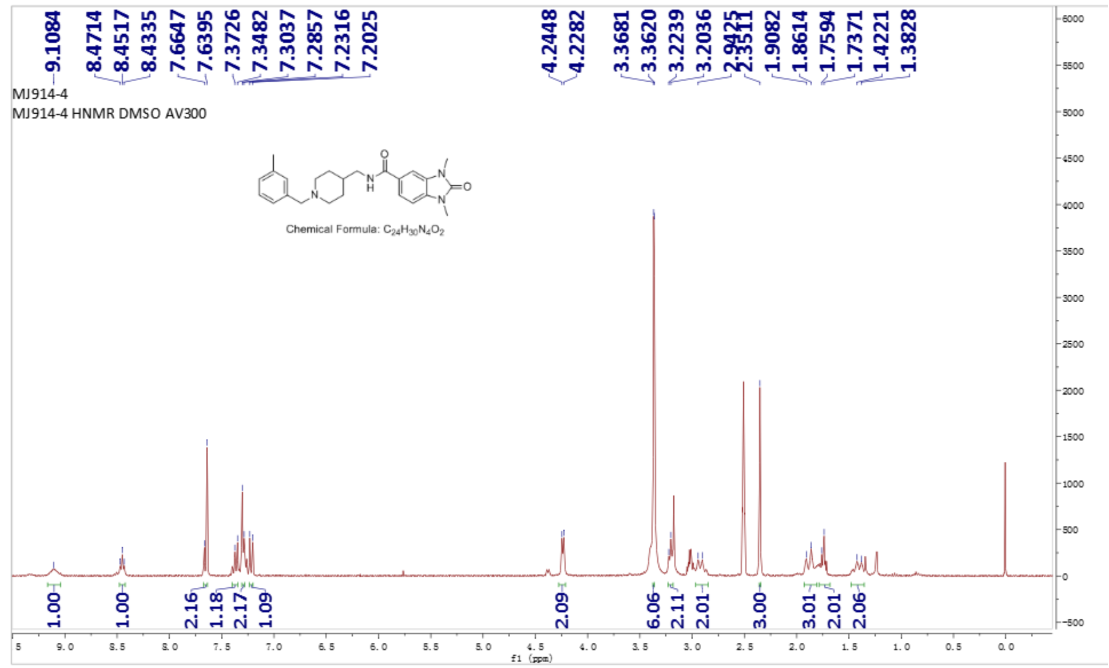

9c. ^{13}C NMR

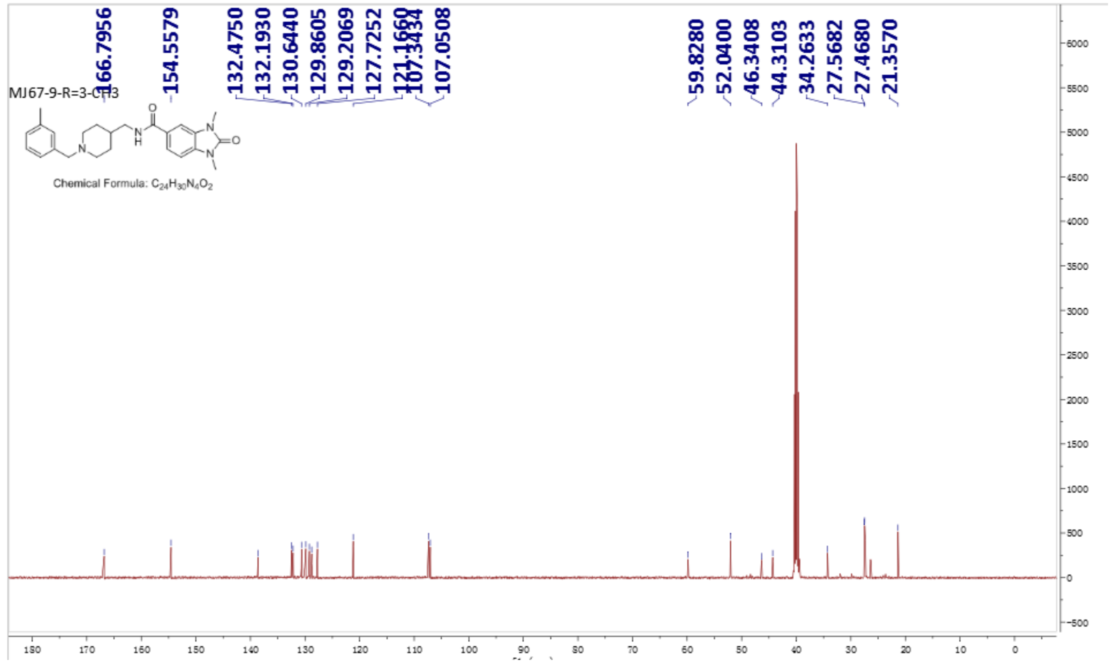

9c. HRMS (ESI)

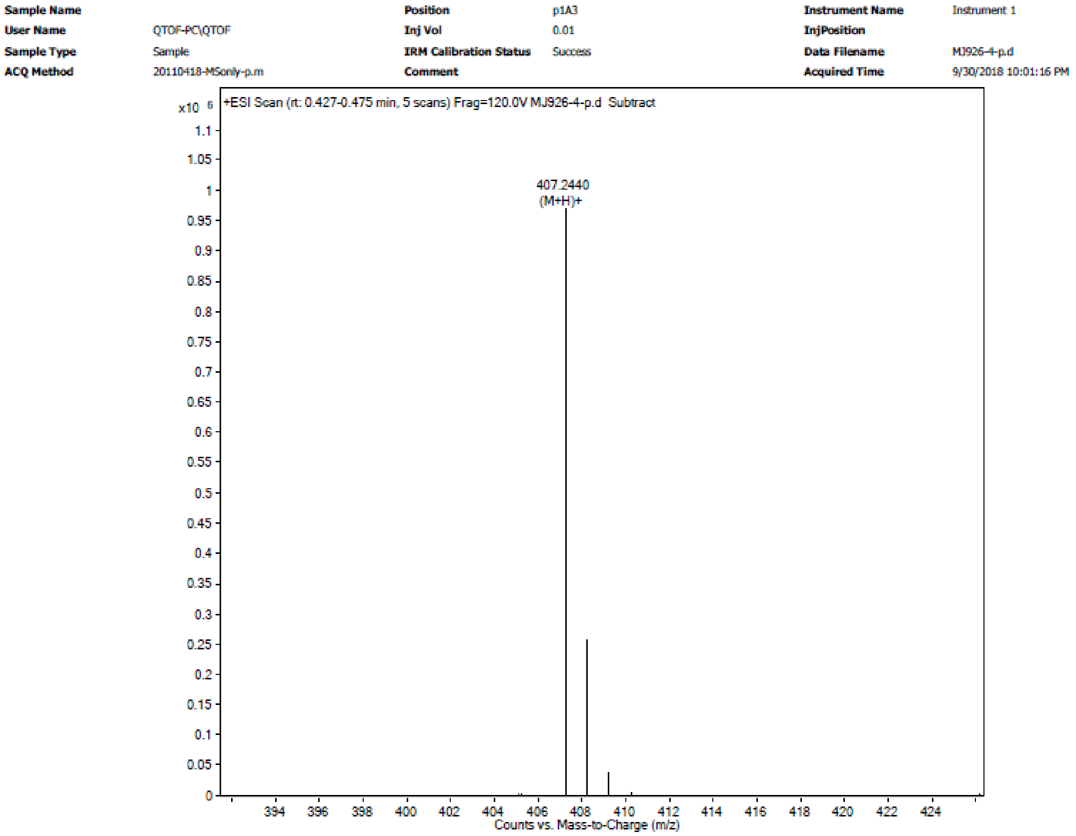

9d. ^{1}H NMR

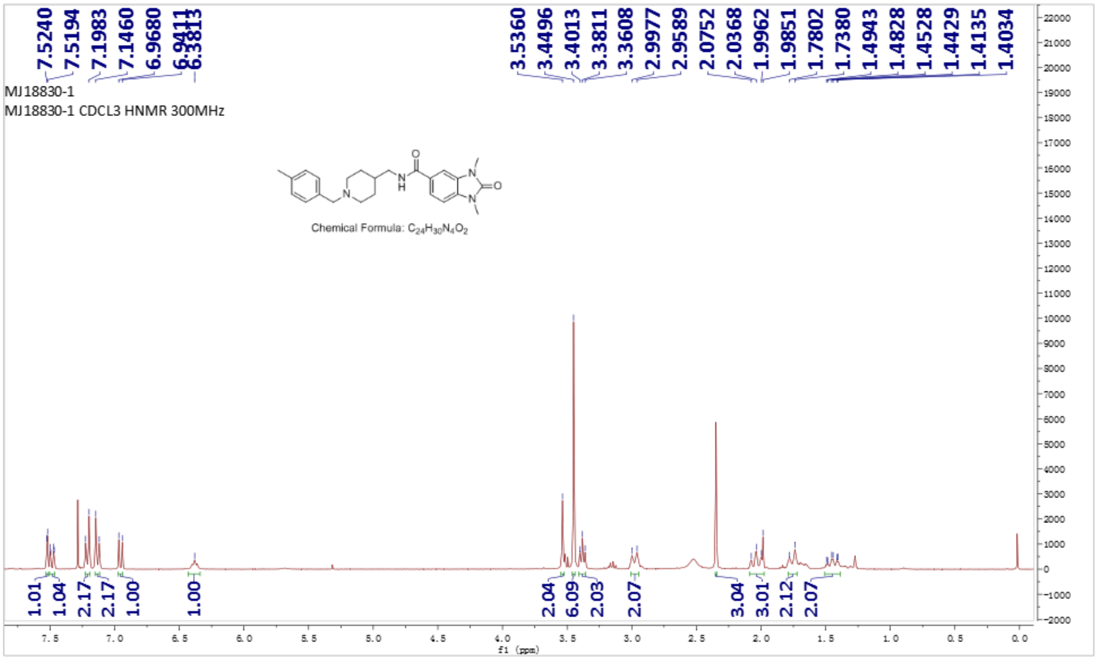

9d. ^{13}C NMR

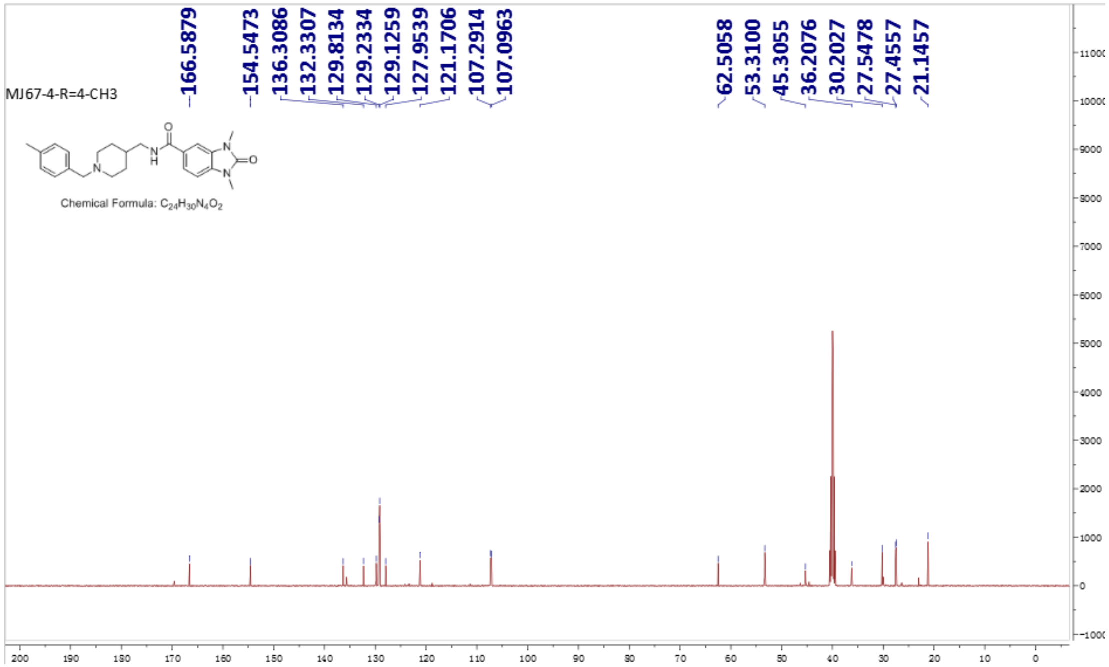

9d. HRMS (ESI)

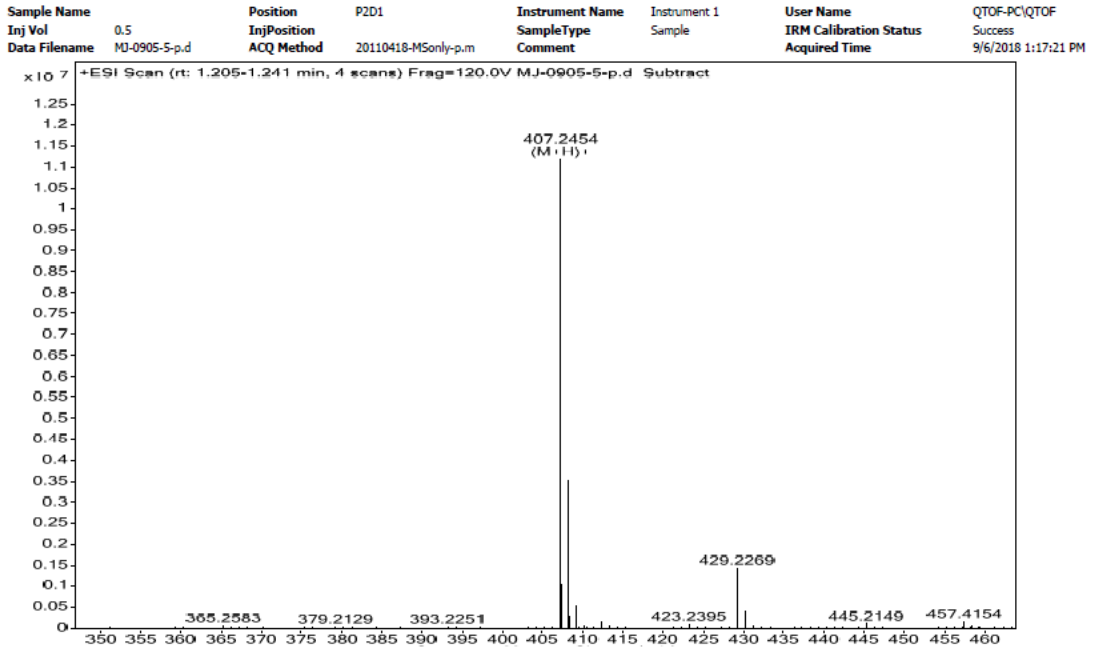

9e. ^{1}H NMR

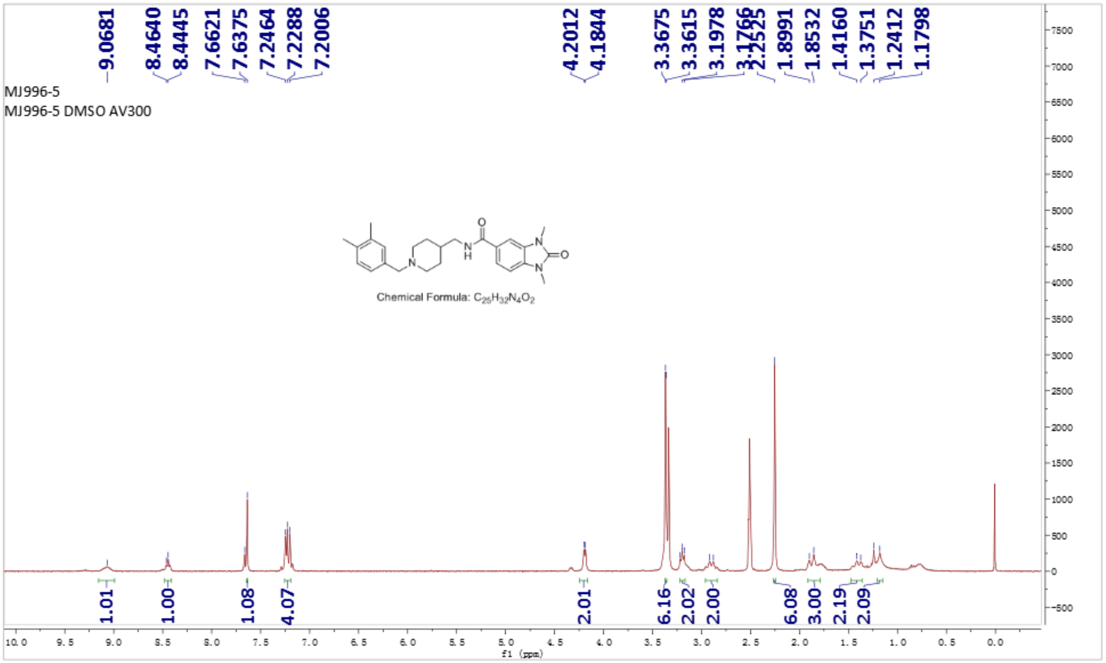

9e. ^{13}C NMR

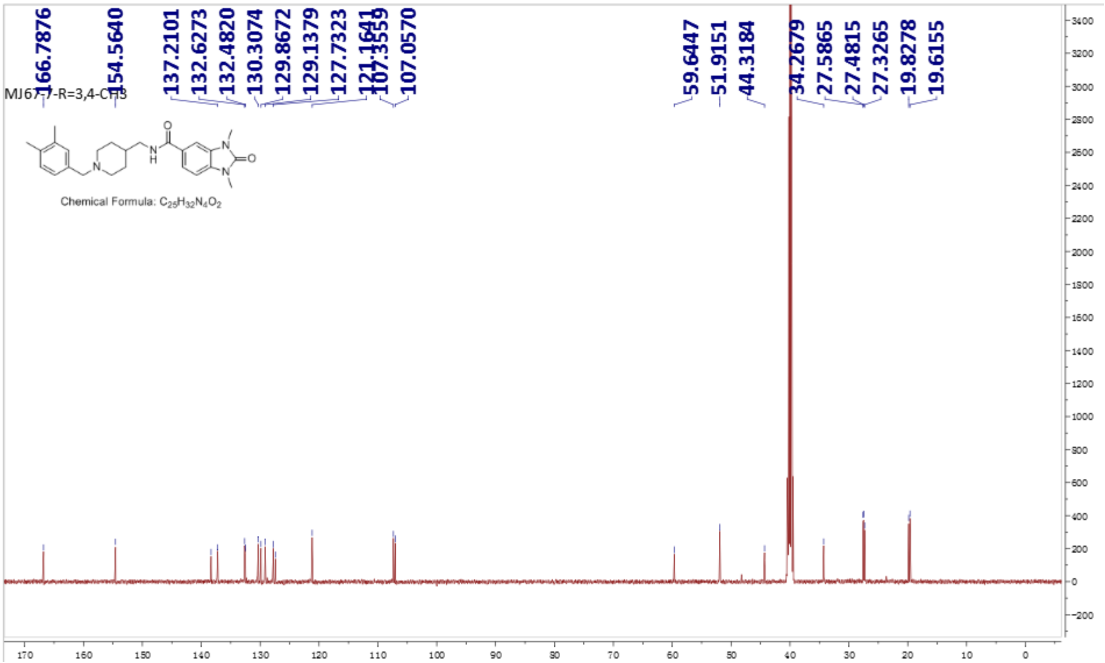

9e. HRMS (ESI)

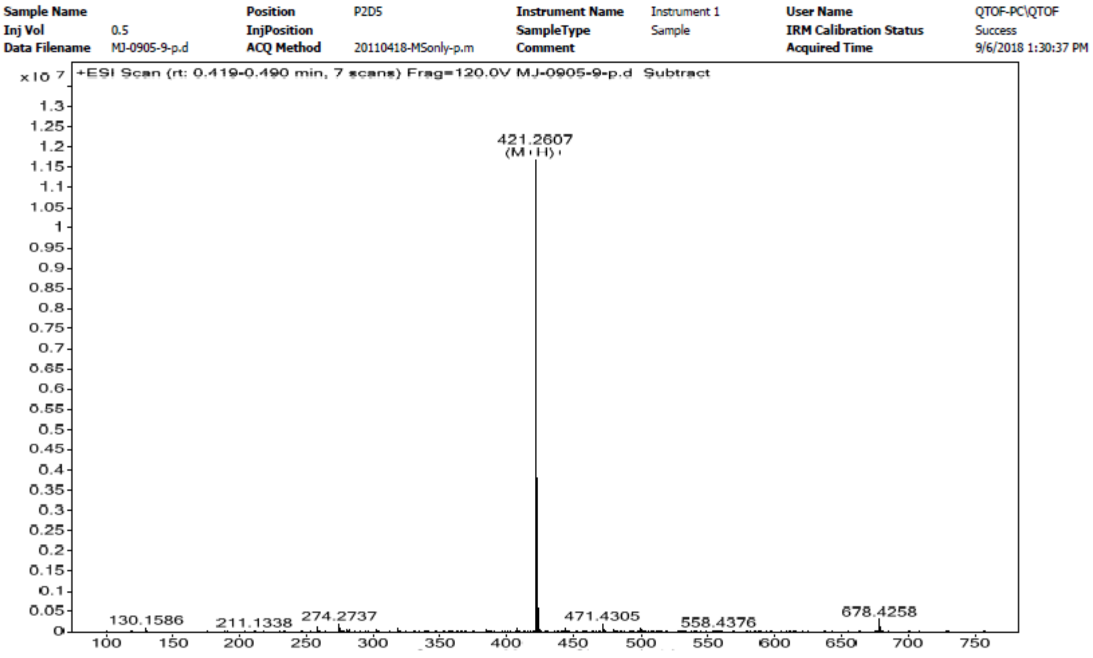

9f. ^{1}H NMR

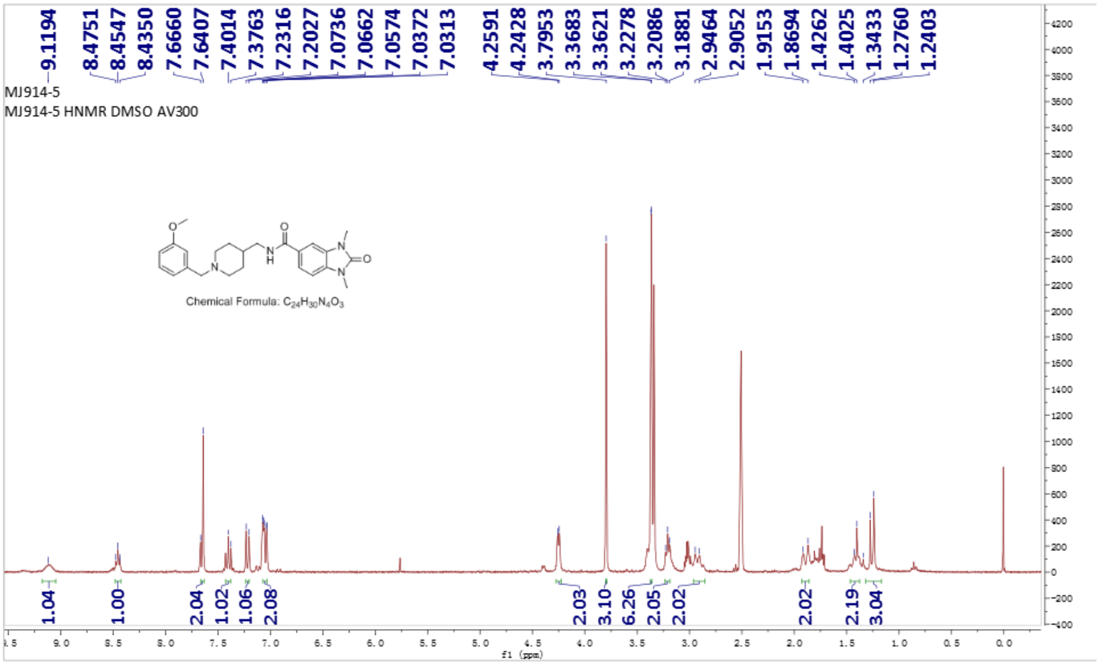

9f. ^{13}C NMR

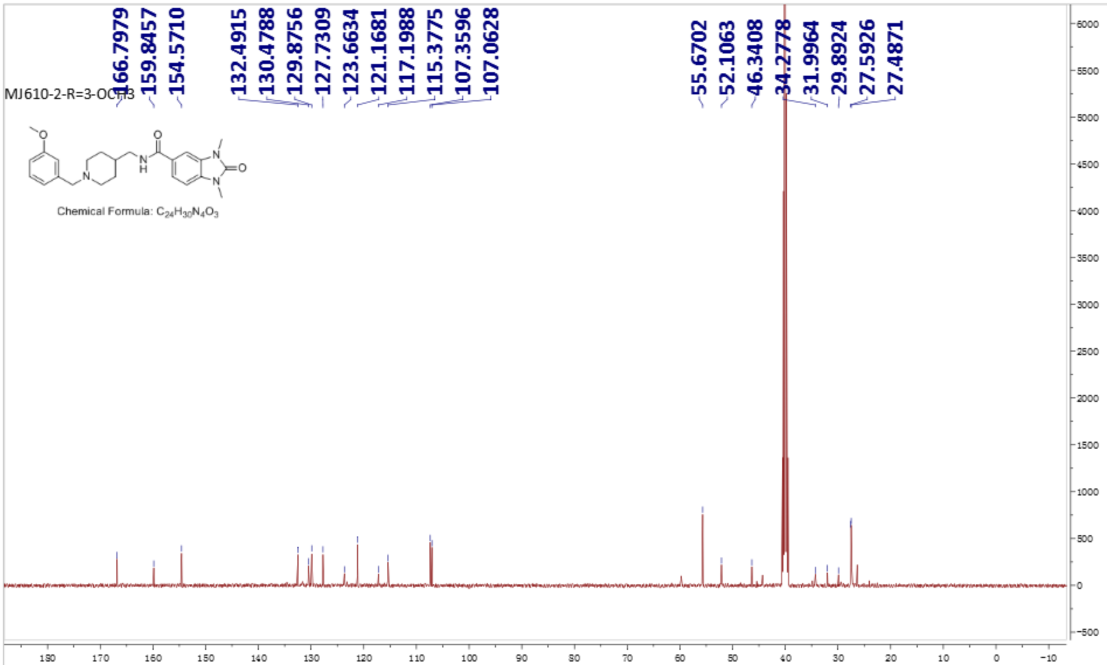

9f. HRMS (ESI)

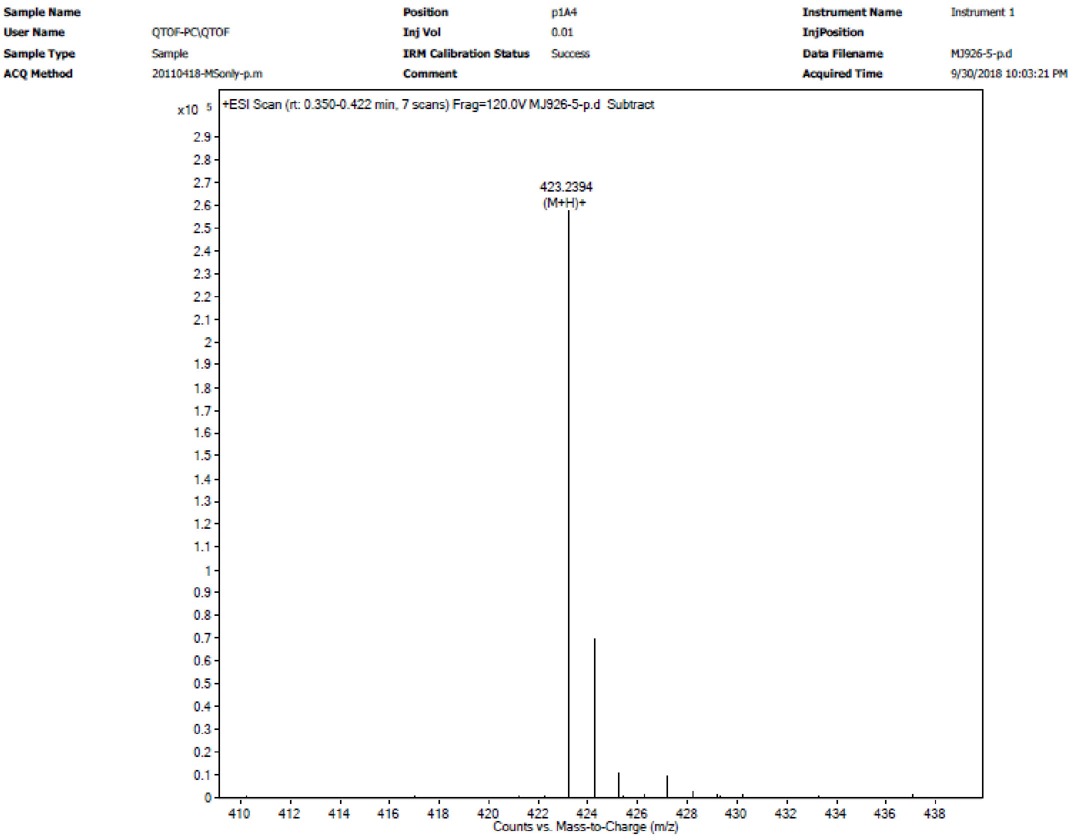

9g. ^{1}H NMR

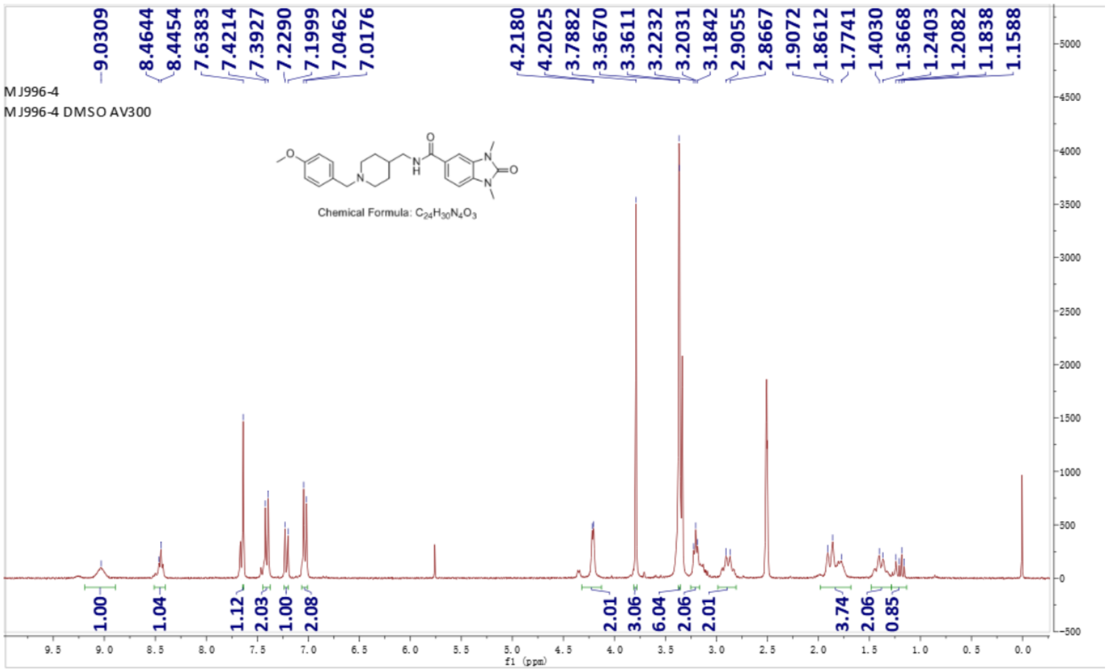

9g. ^{13}C NMR

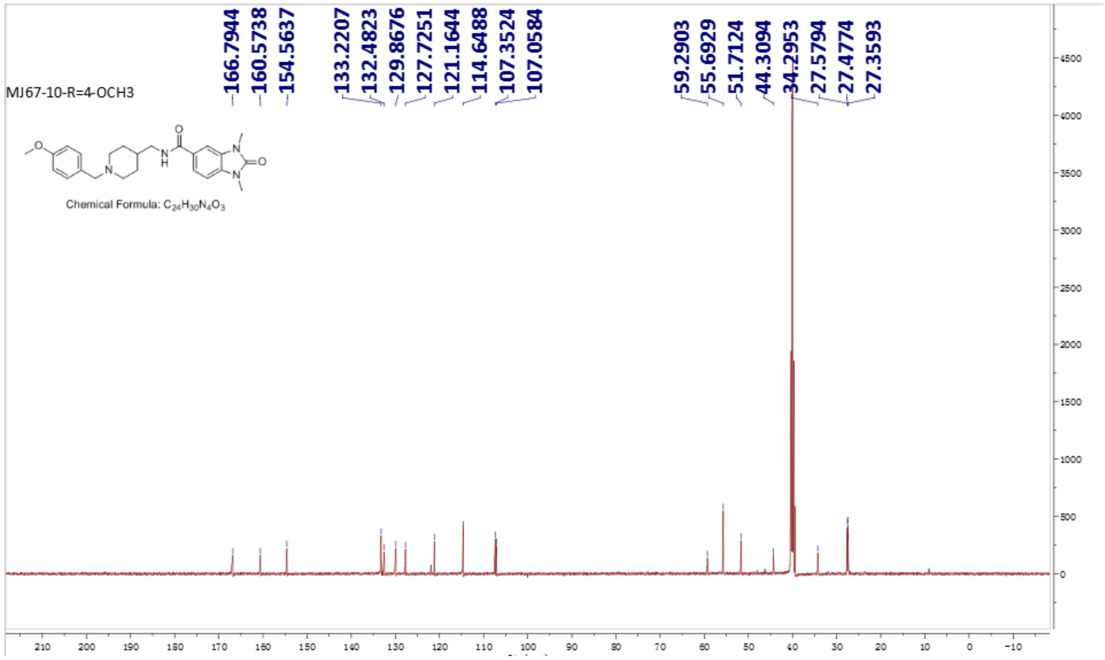

9g. HRMS (ESI)

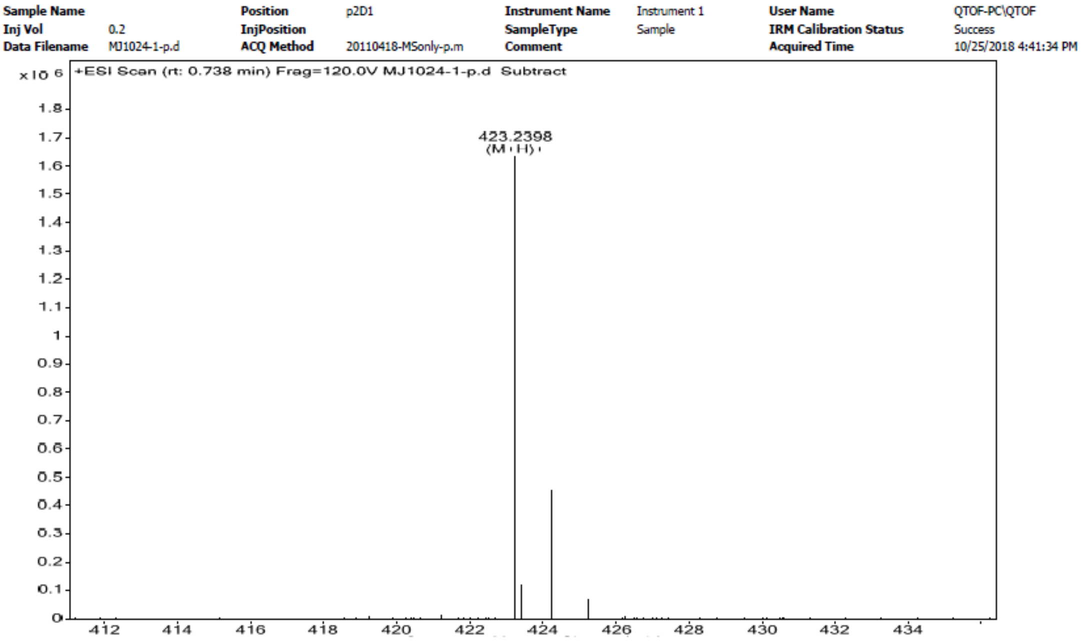

9h. ^{1}H NMR

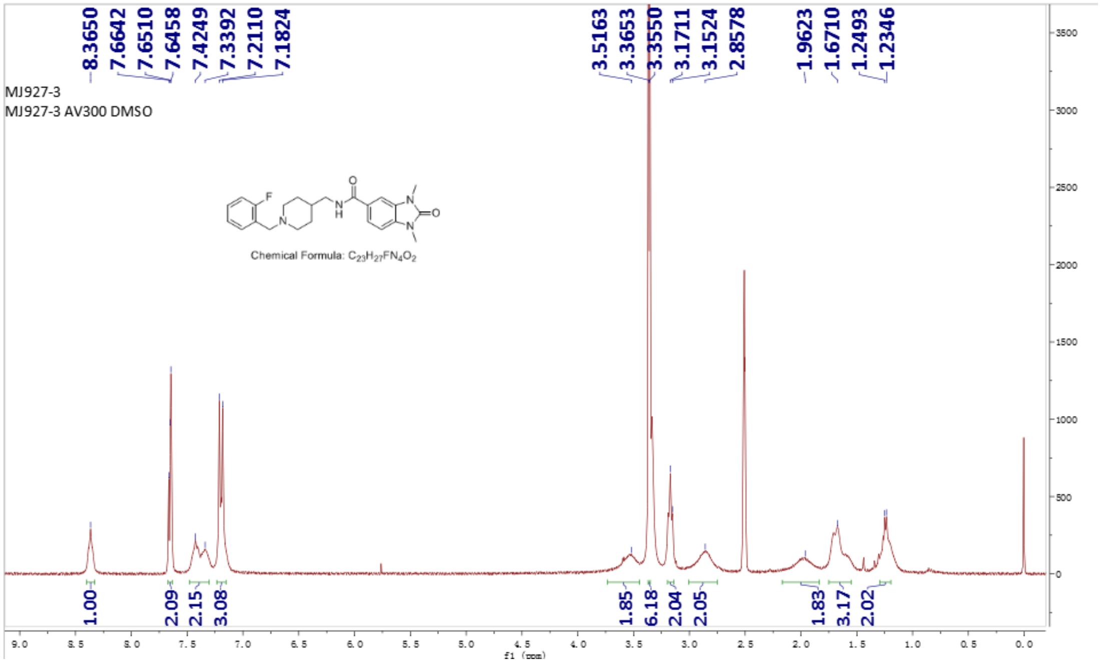

9h. ^{13}C NMR

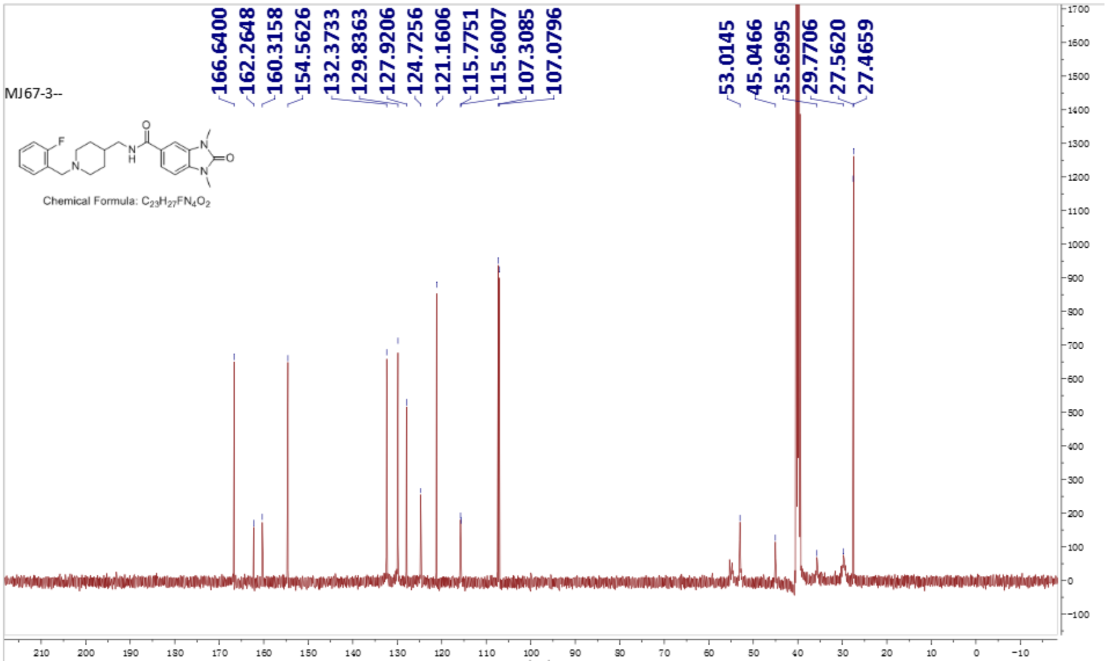

9h. HRMS (ESI)

Sample Name		Position	p1A5	Instrument Name	Instrument 1
User Name	QTOF-PC\QTOF	Inj Vol	0.01	InjPosition	
Sample Type	Sample	IRM Calibration Status	Success	Data Filename	MJ926-6-p.d
ACQ Method	20110418-MSonly-p.m	Comment		Acquired Time	9/30/2018 10:05:32 PM

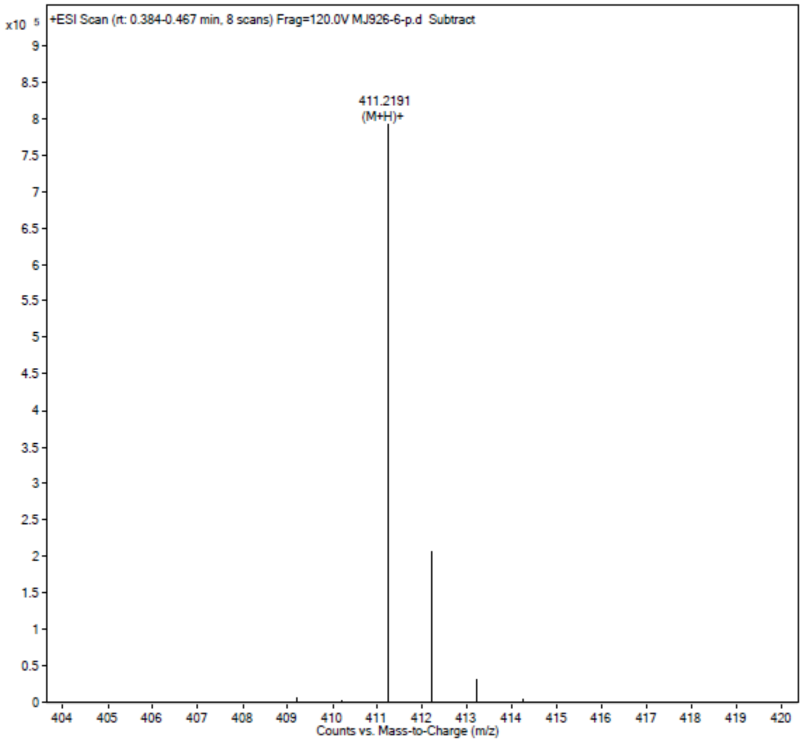

9i. ^{1}H NMR

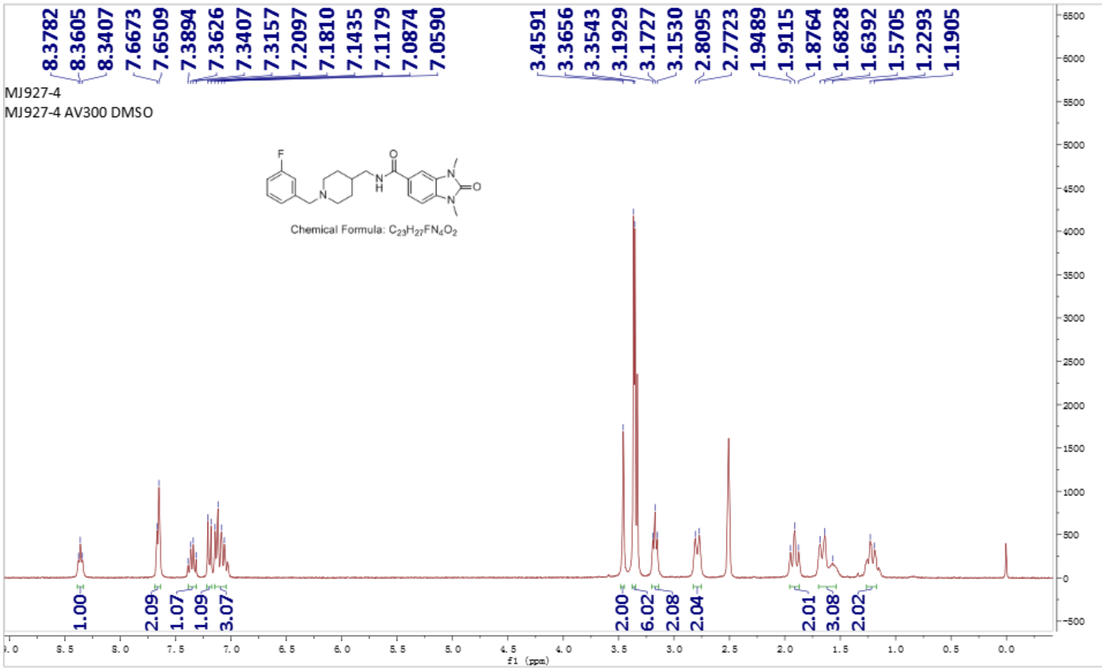

9i. ^{13}C NMR

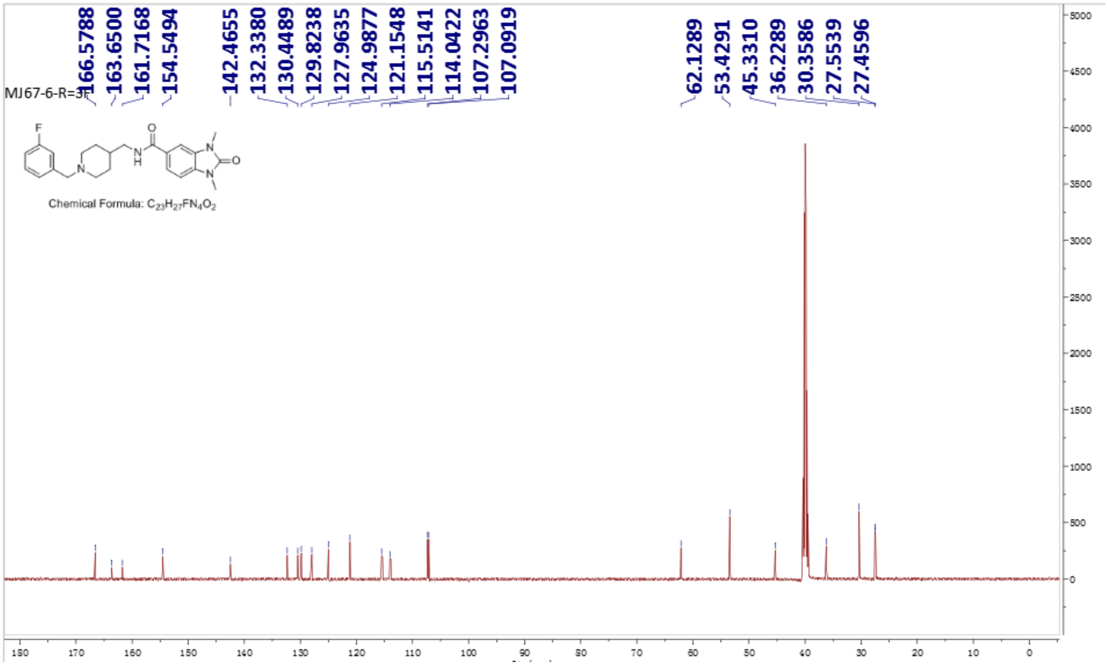

9i. HRMS (ESI)

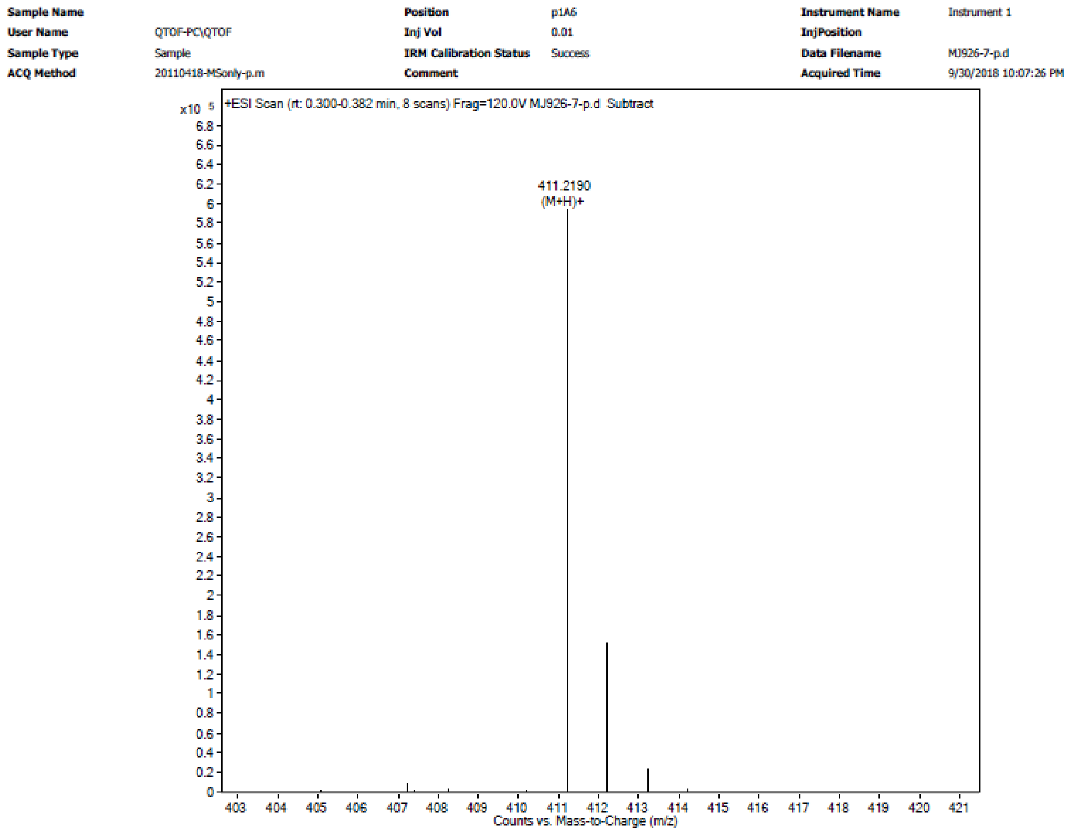

9j. ^{1}H NMR

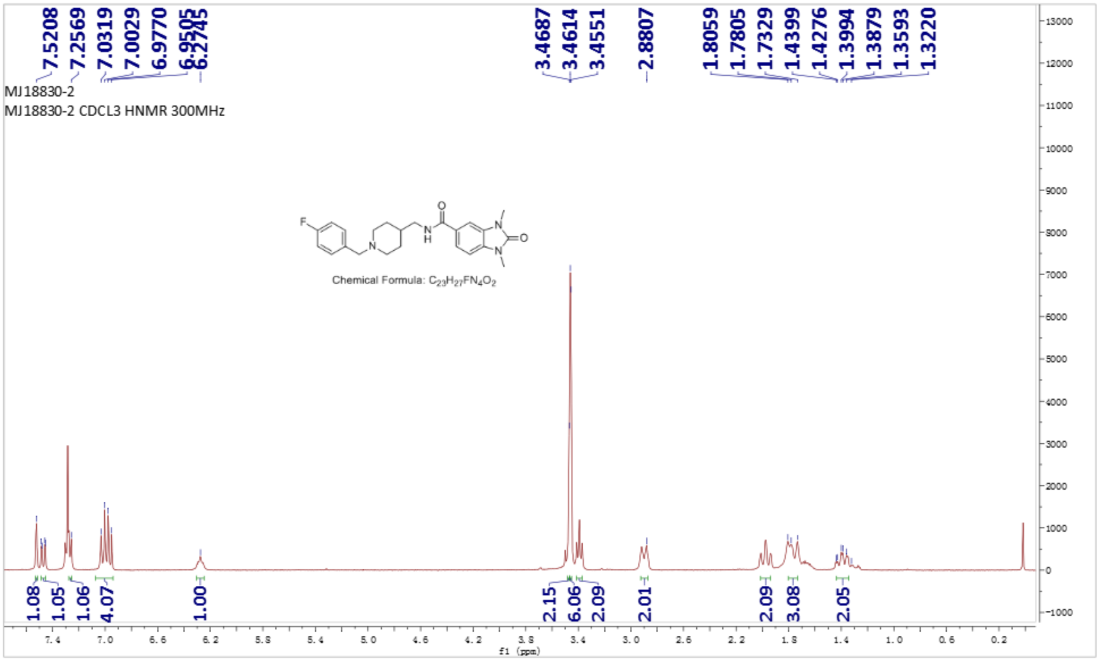

9j. ^{13}C NMR

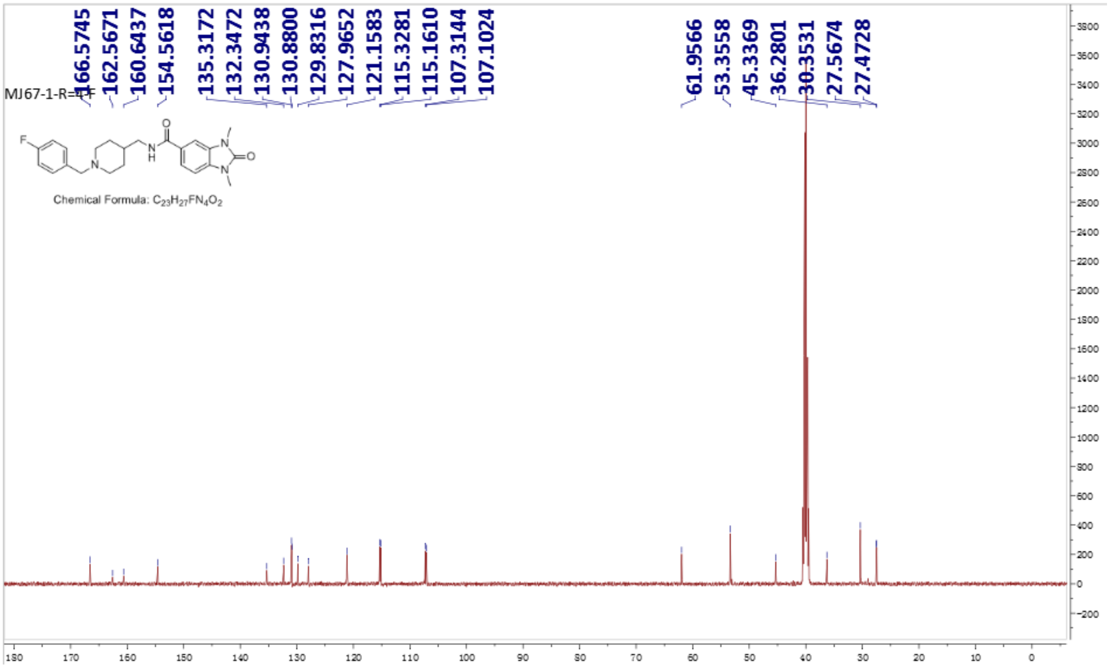

9j. HRMS (ESI)

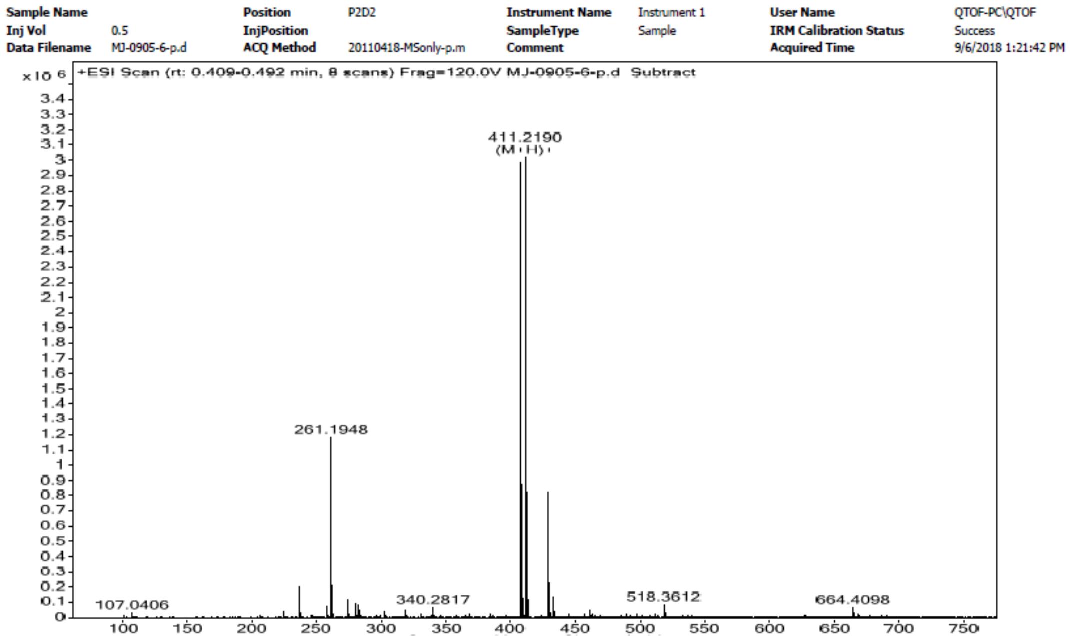

9k. ^{1}H NMR

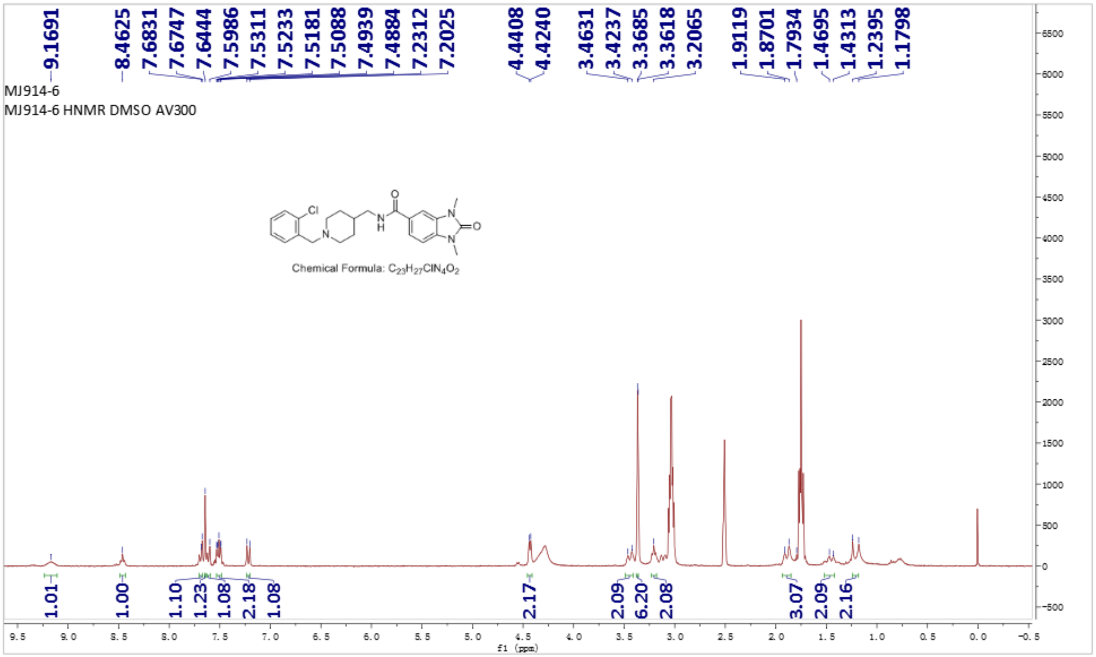

9k. ^{13}C NMR

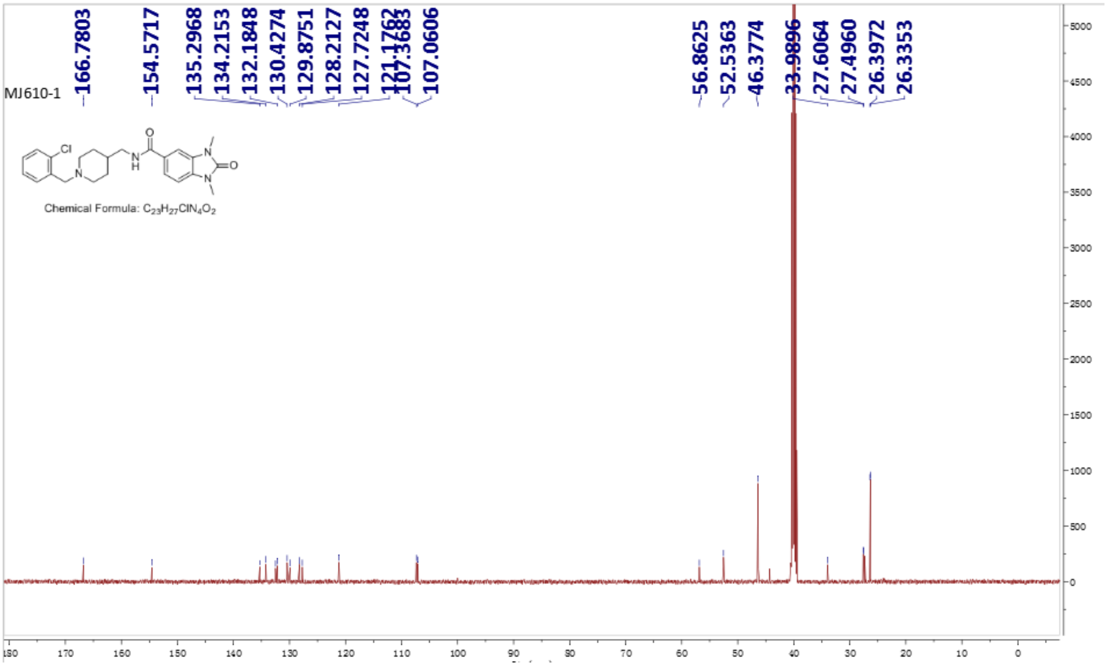

9k. HRMS (ESI)

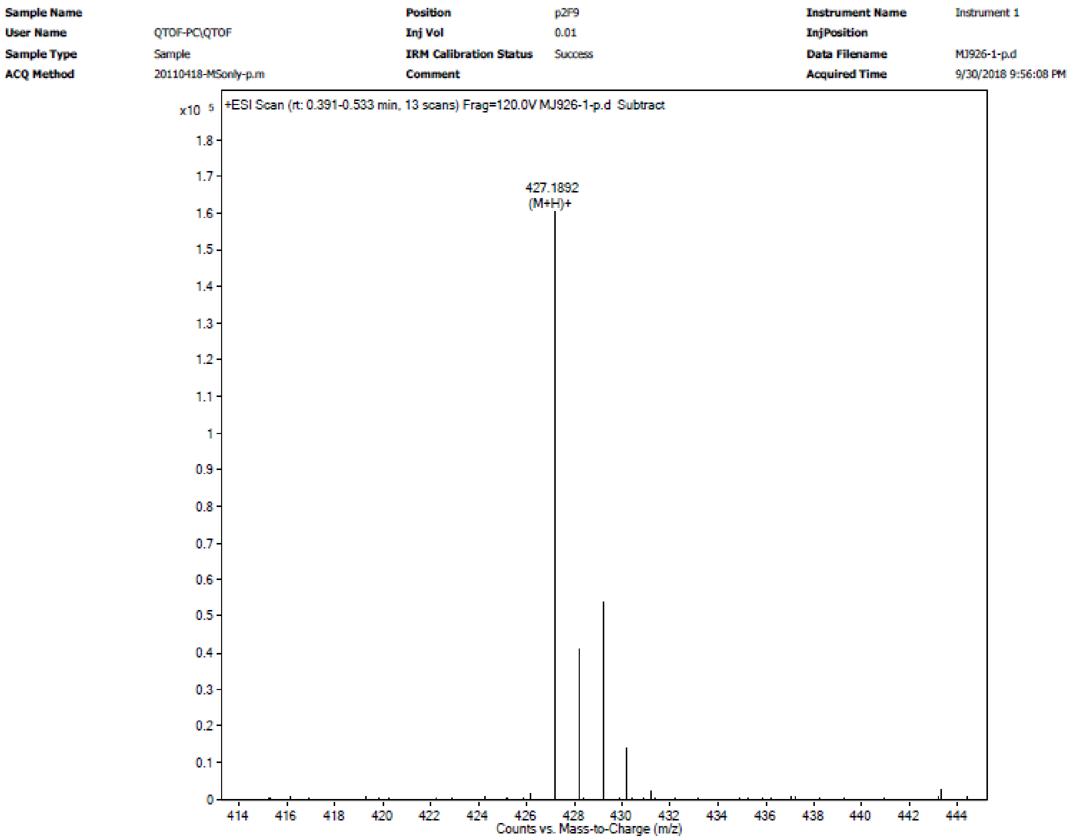

9l. ^{1}H NMR

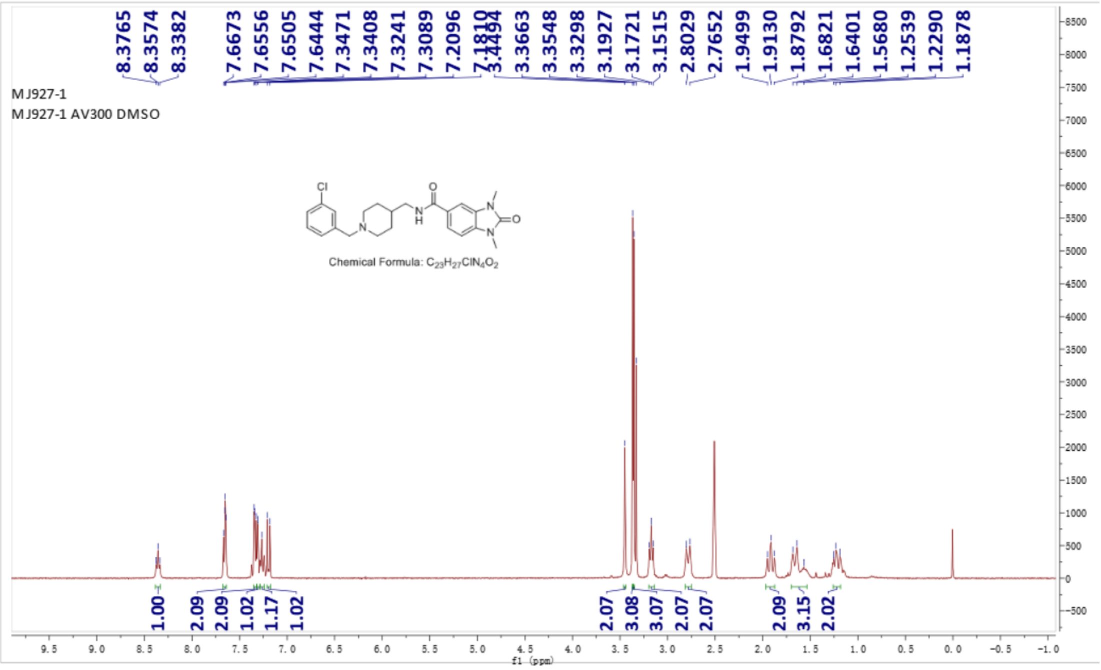

9l. ^{13}C NMR

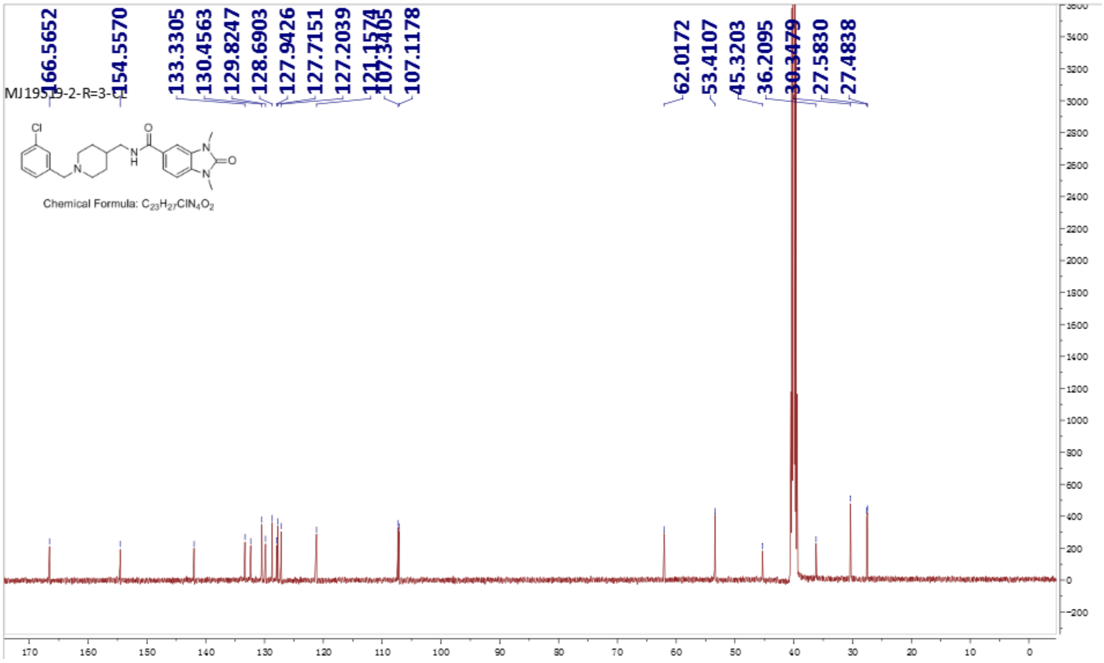

9l. HRMS (ESI)

**Sample Name**		**Position**	p1A9	**Instrument Name**	Instrument 1
**User Name**	QTOF-PC\QTOF	**Inj Vol**	0.01	**InjPosition**	
**Sample Type**	Sample	**IRM Calibration Status**	Success	**Data Filename**	MJ926-10-p.d
**ACQ Method**	20110418-MSonly-p.m	**Comment**		**Acquired Time**	9/30/2018 10:13:29 PM

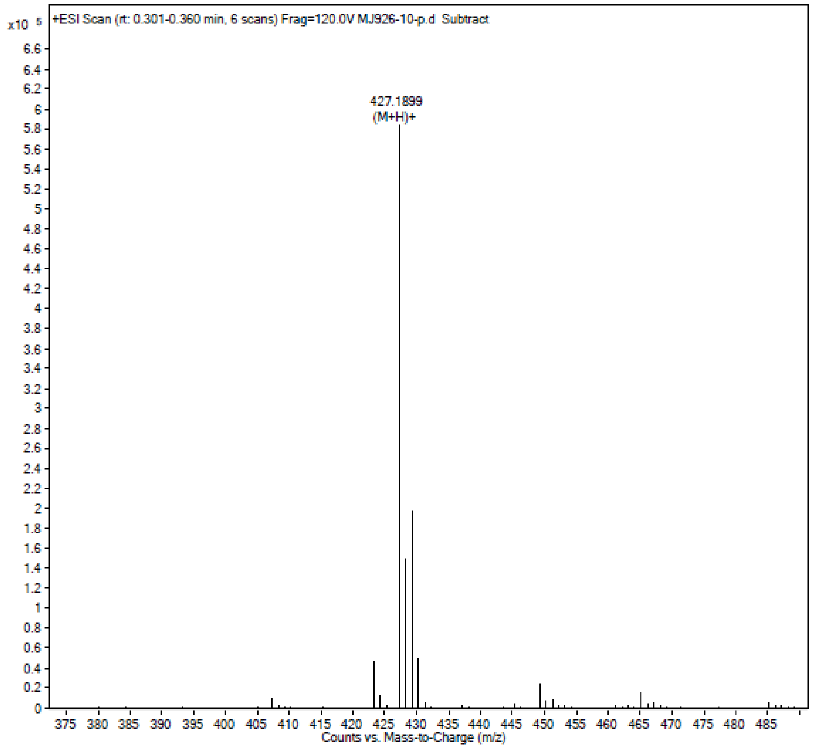

9m. ^{1}H NMR

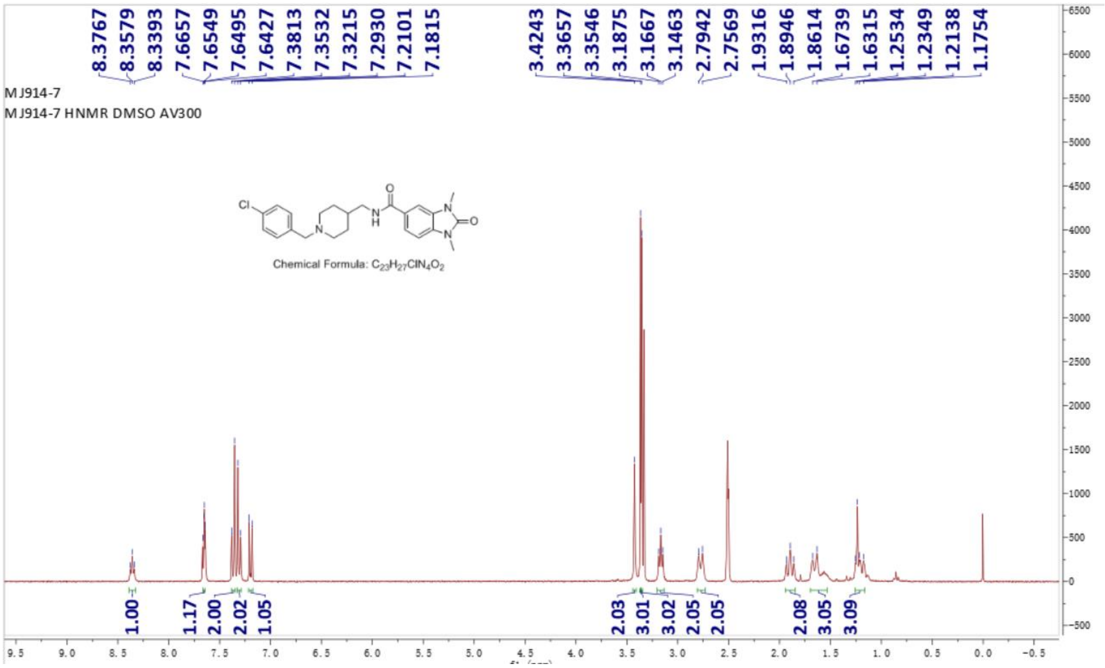

9m. ^{13}C NMR

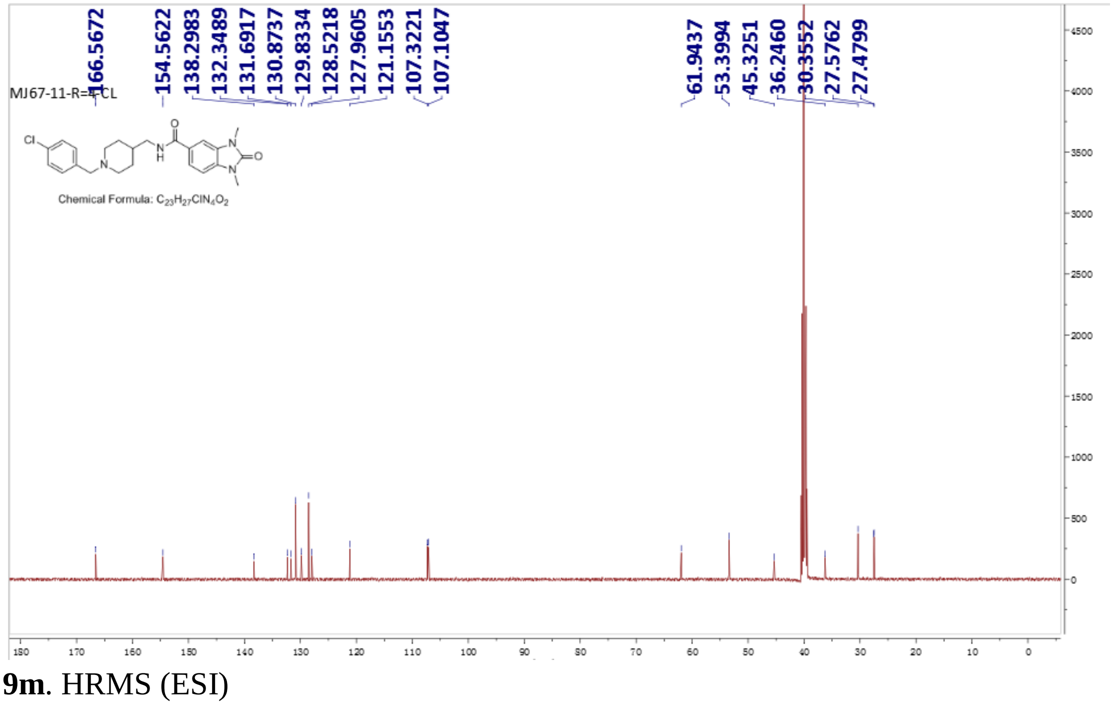

9m. HRMS (ESI)

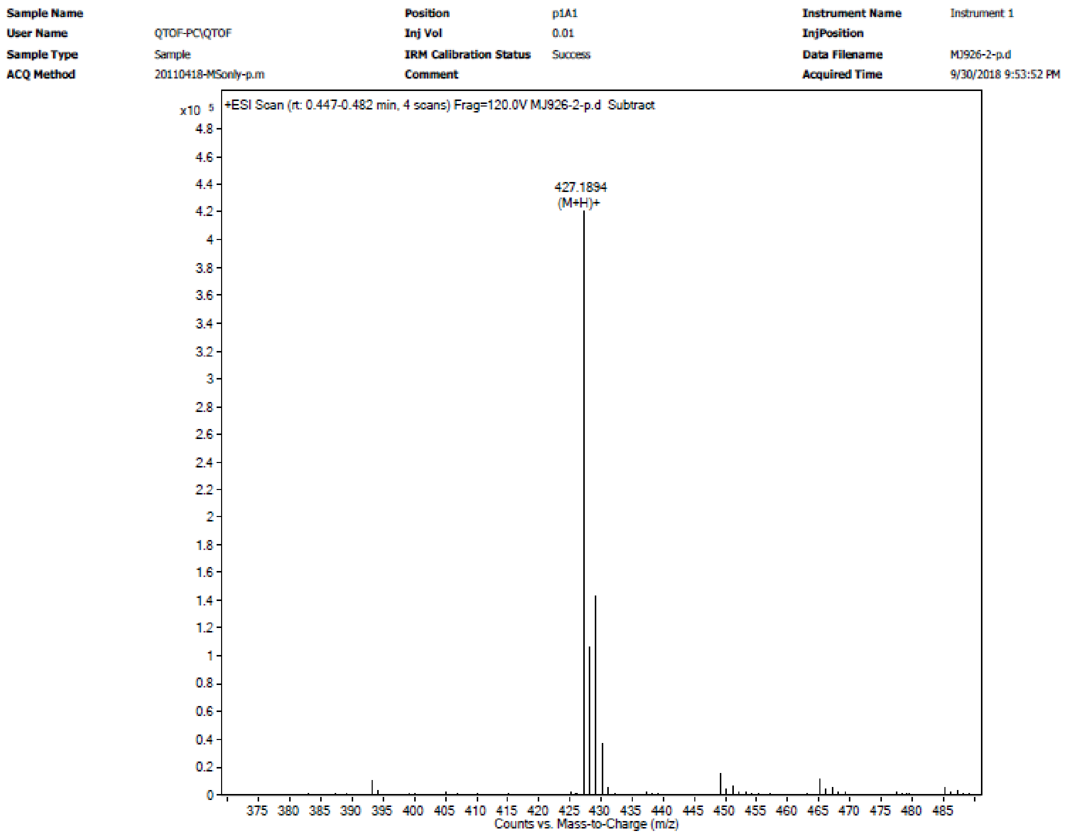

9n. ^{1}H NMR

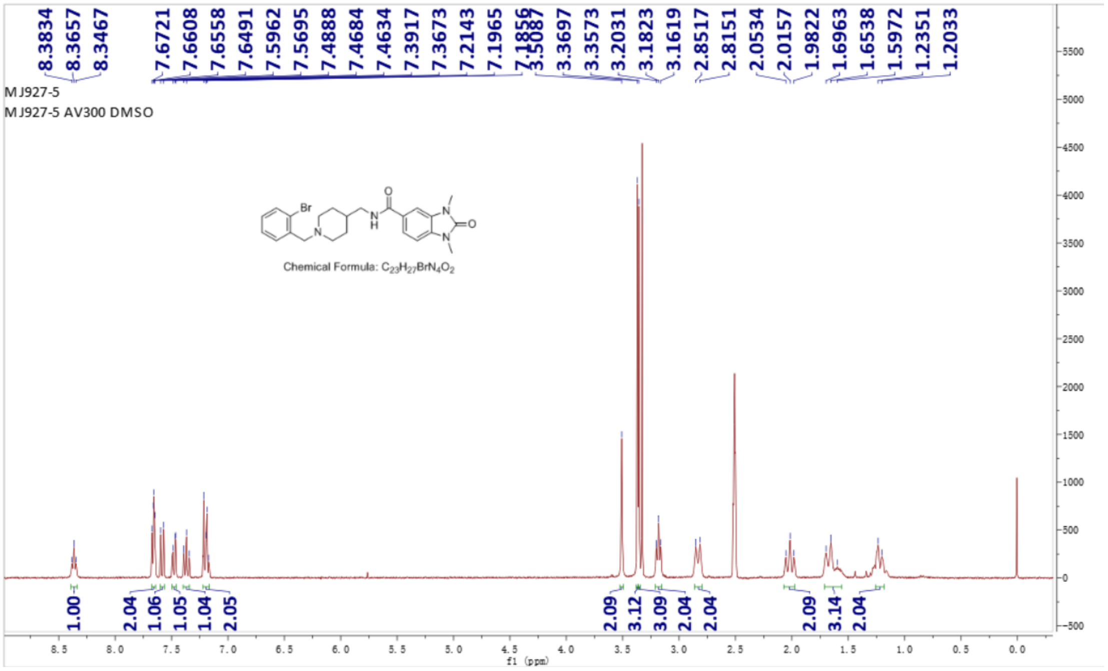

9n. ^{13}C NMR

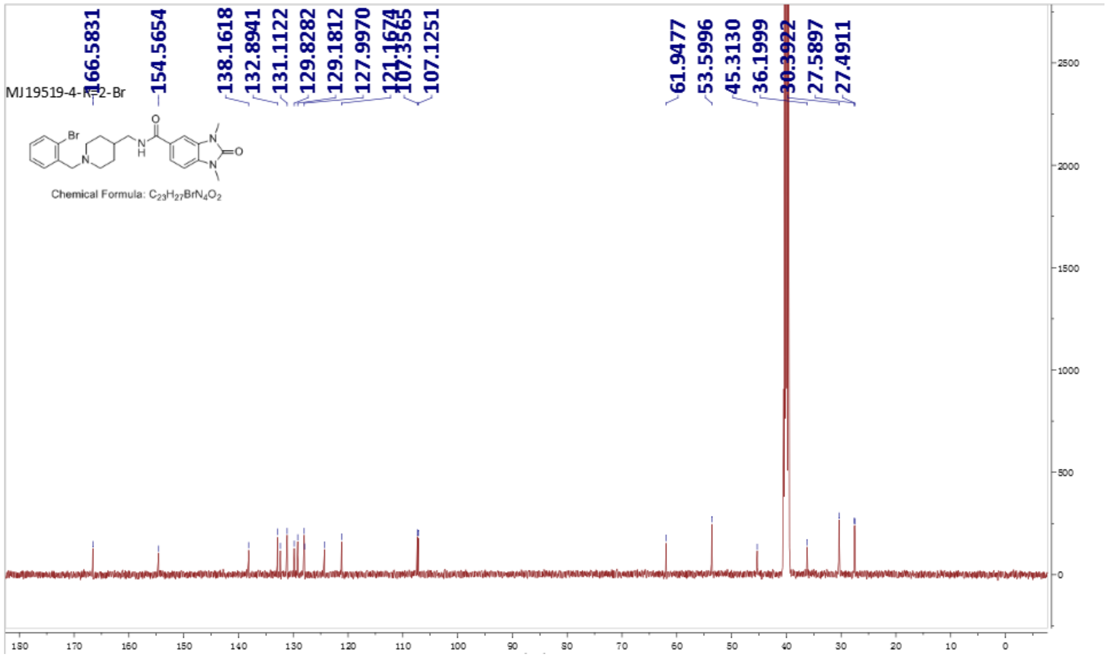

9n. HRMS (ESI)

**Sample Name**		**Position**	p1A7	**Instrument Name**	Instrument 1
**User Name**	QTOF-PC\QTOF	**Inj Vol**	0.01	**InjPosition**	
**Sample Type**	Sample	**IRM Calibration Status**	Success	**Data Filename**	MJ926-8-p.d
**ACQ Method**	20110418-MSonly-p.m	**Comment**		**Acquired Time**	9/30/2018 10:09:14 PM

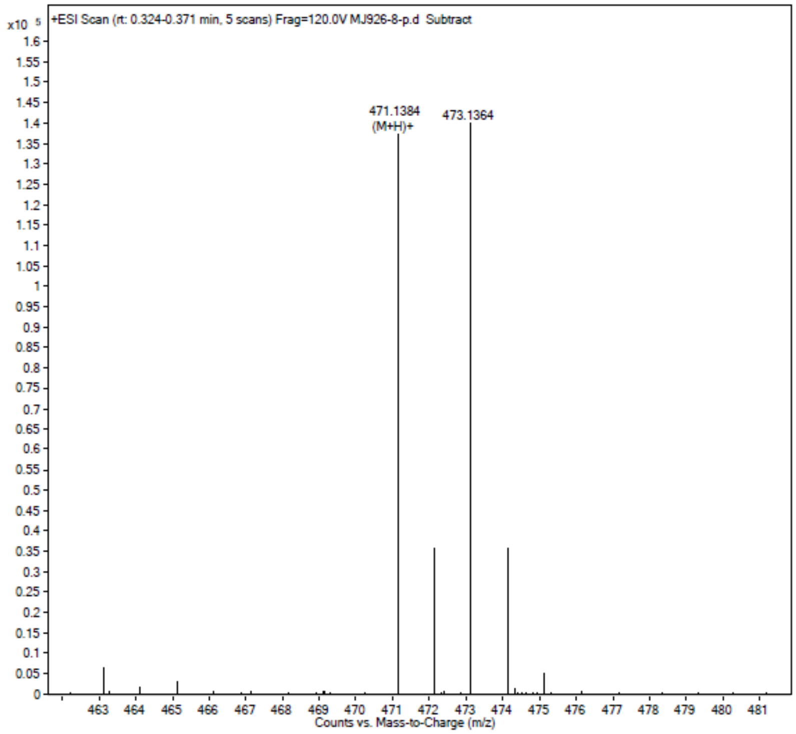

9o. ^{1}H NMR

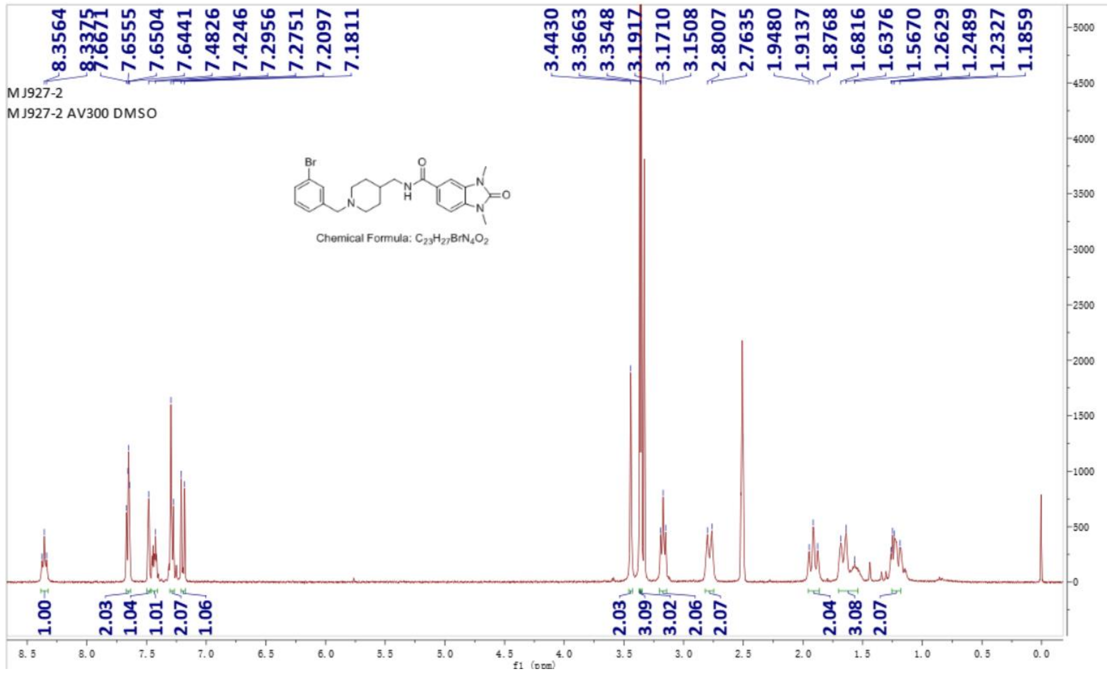

9o. ^{13}C NMR

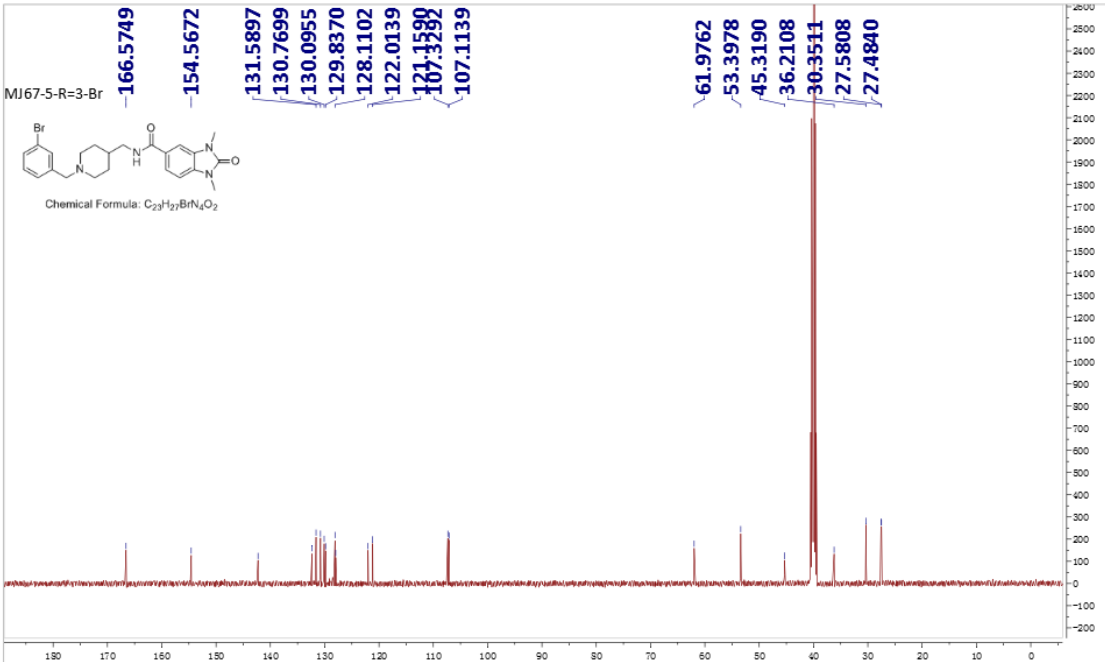

9o. HRMS (ESI)

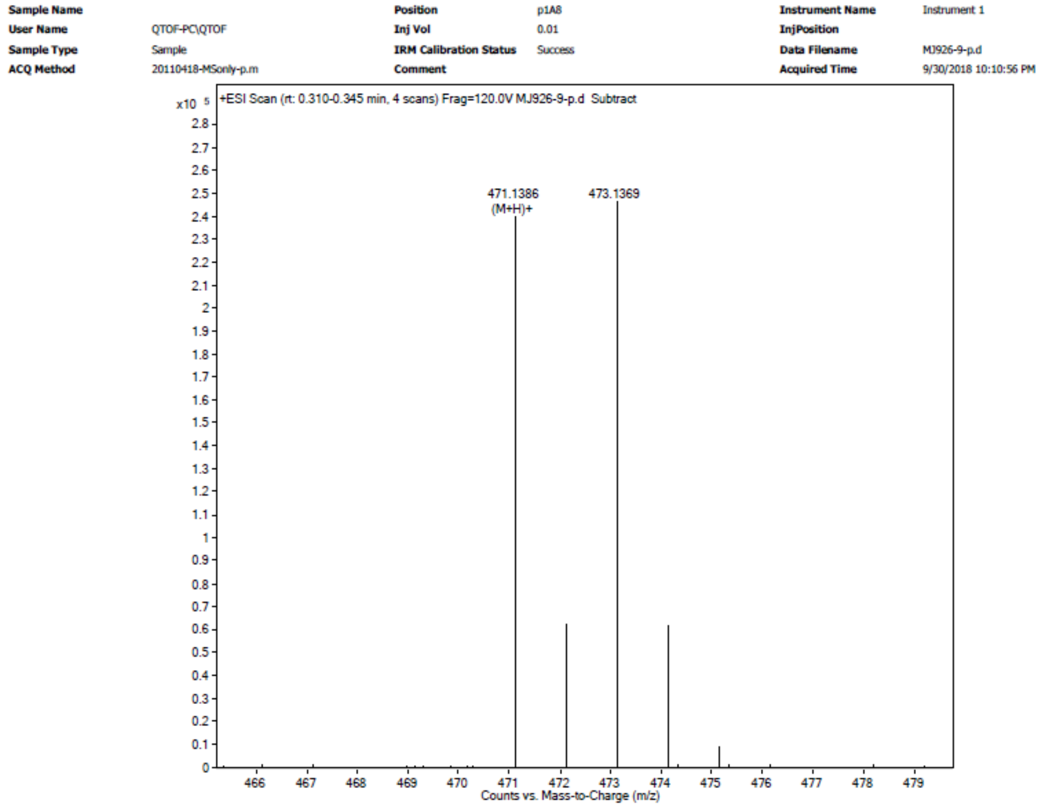

9p. ^{1}H NMR

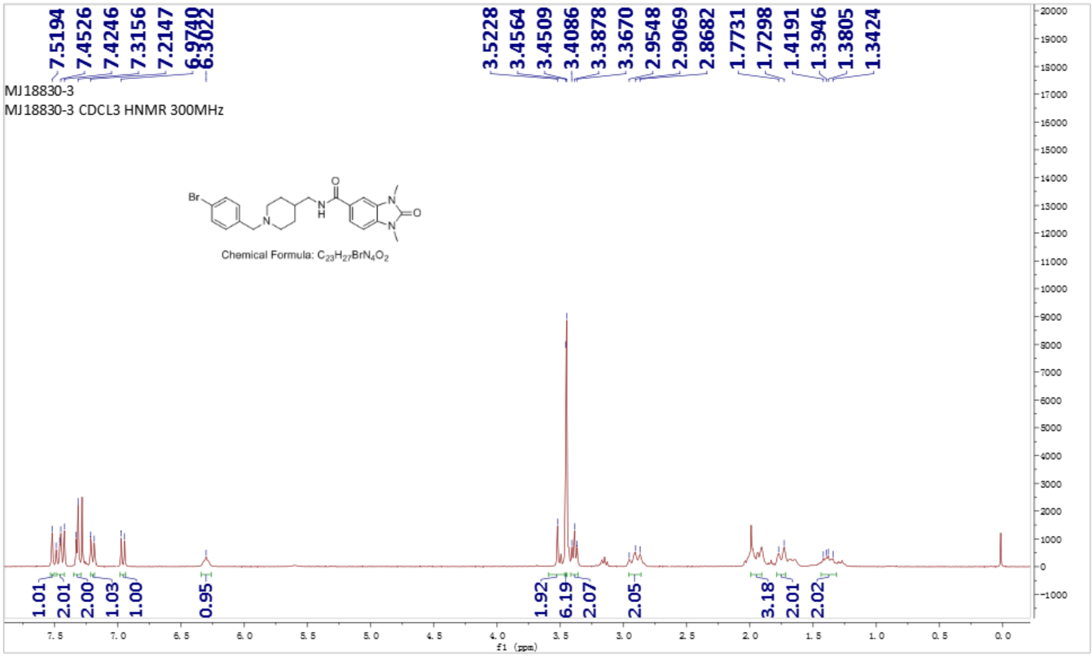

9p. ^{13}C NMR

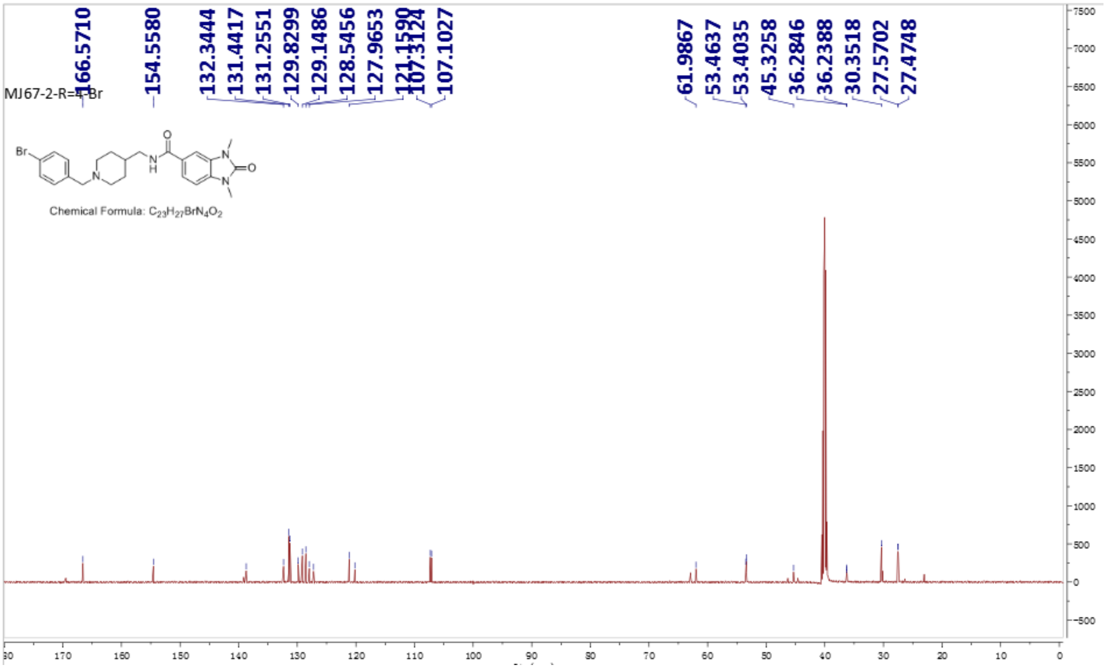

9p. HRMS (ESI)

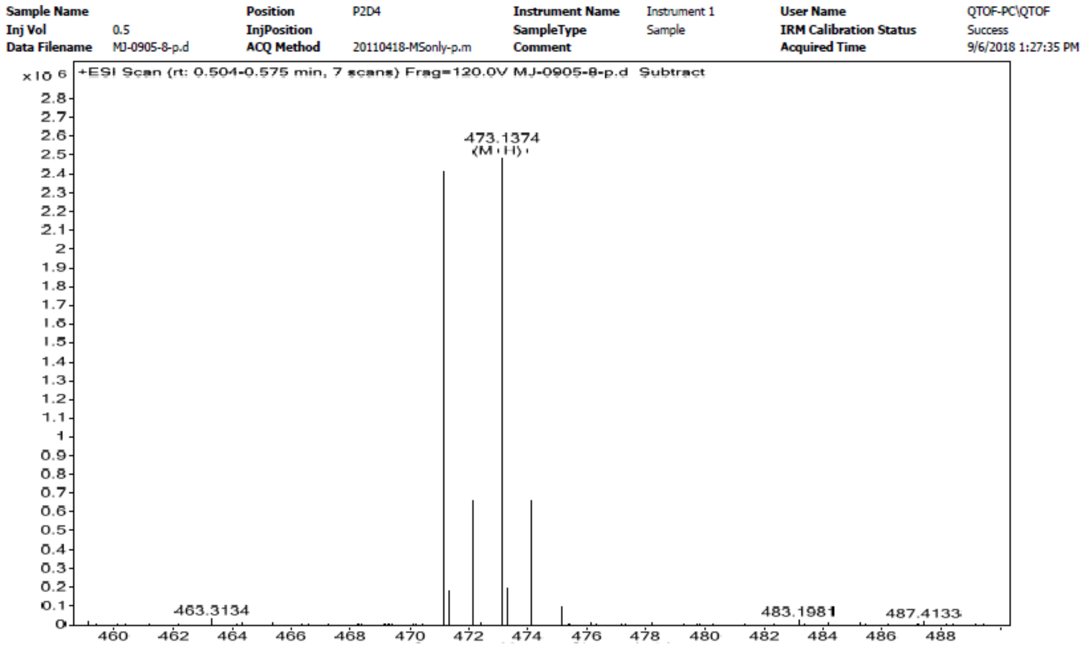

11a.^{1}H NMR

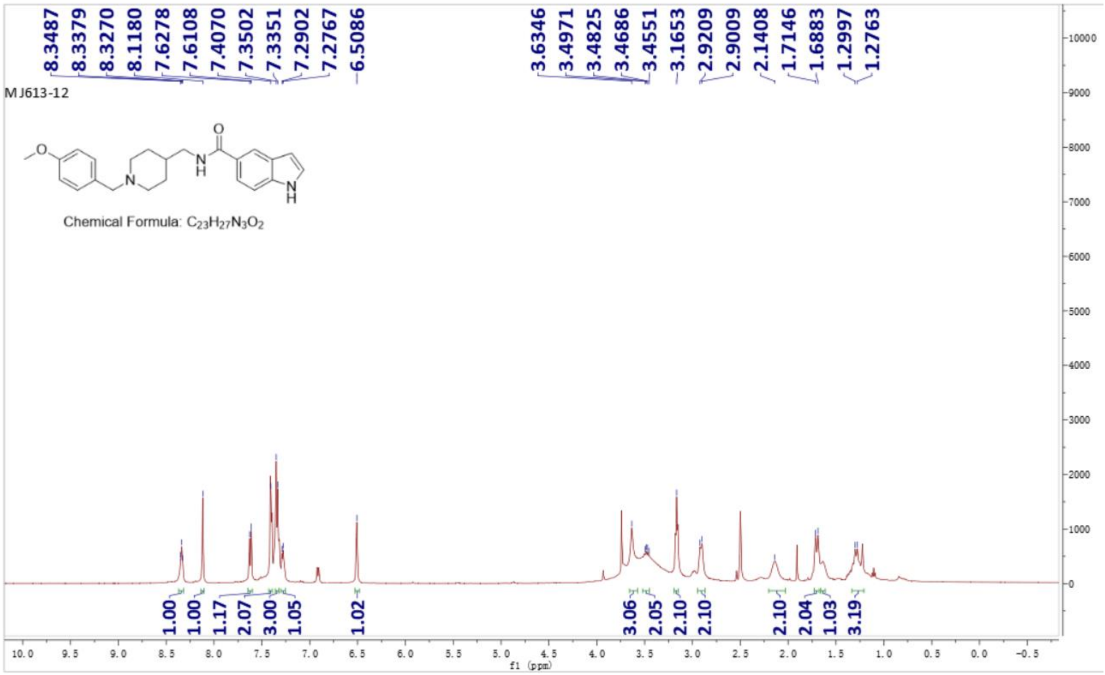

11a.^{13}C NMR

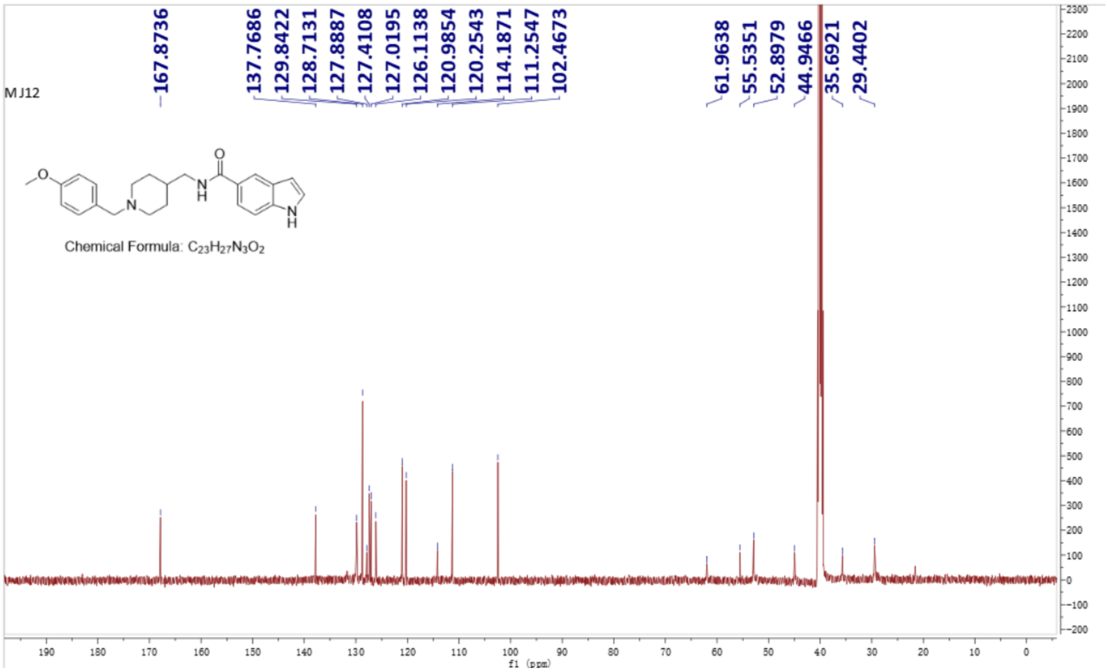

11a.HRMS (ESI)

	**Position**	P2B2	**Instrument Name**	Instrument 1
QTOF-PC\QTOF	**Inj Vol**	0.1	**InjPosition**	
Sample	**IRM Calibration Status**	Success	**Data Filename**	MJ-7-p.d
20110418-MSonly-p.m	**Comment**		**Acquired Time**	6/18/2019 10:53:52 AM

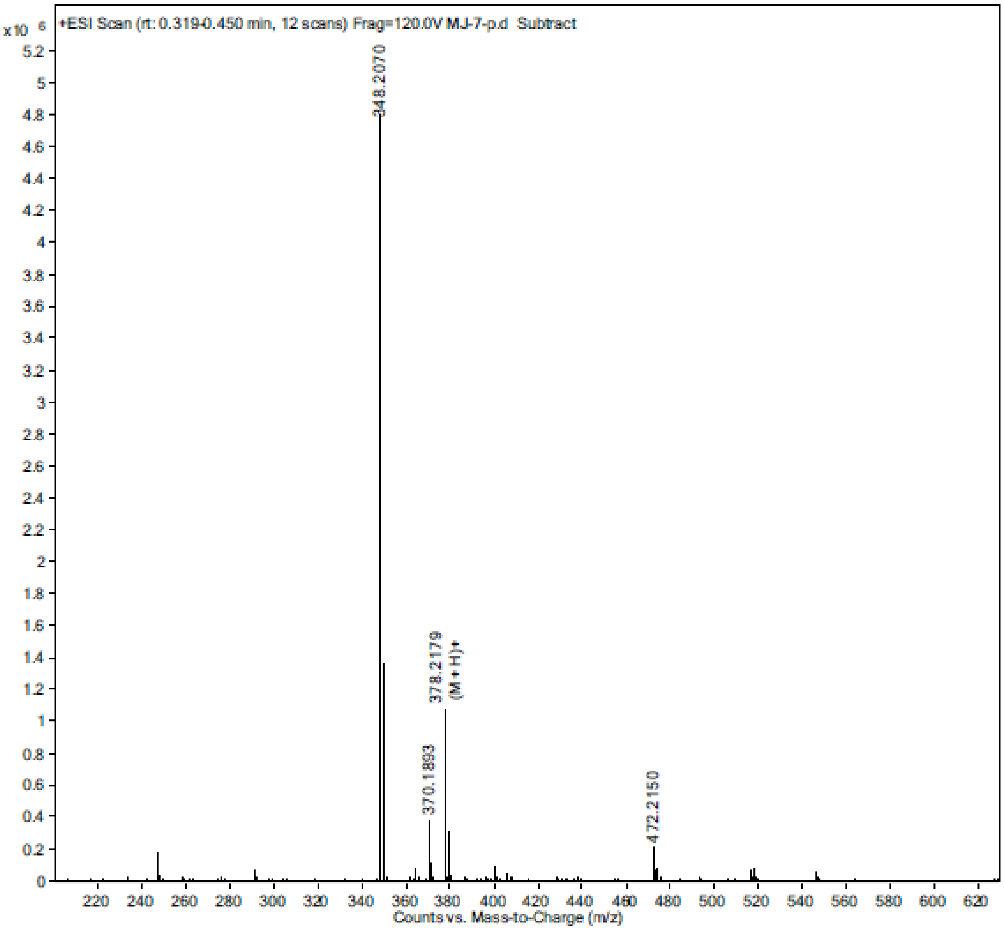

11b.^{1}H NMR

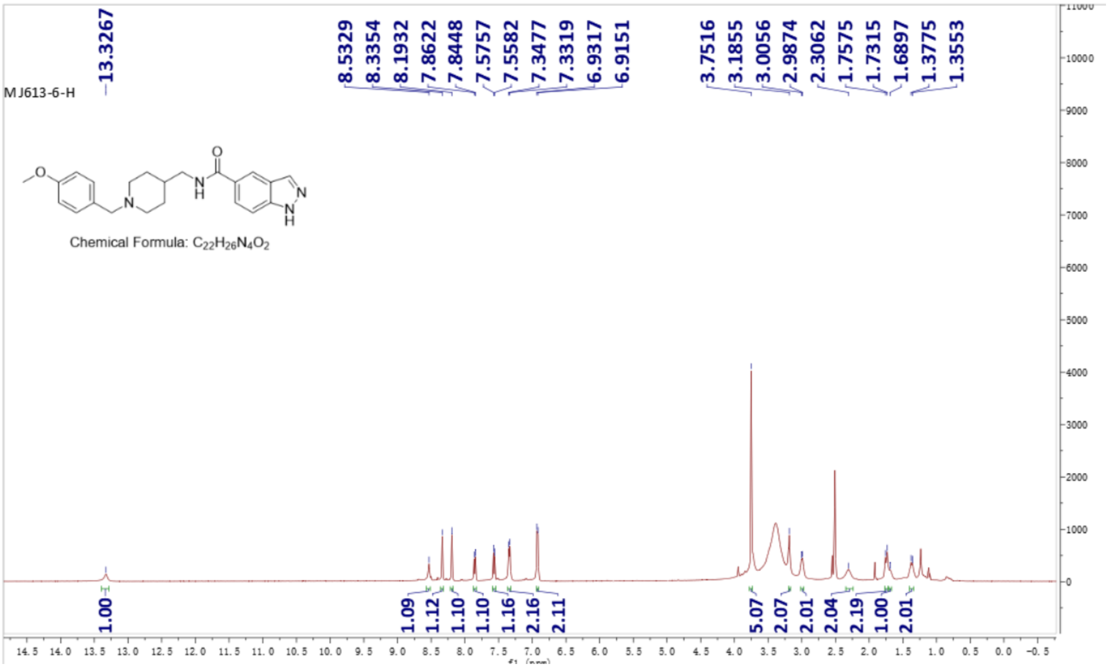

11b.^{13}C NMR

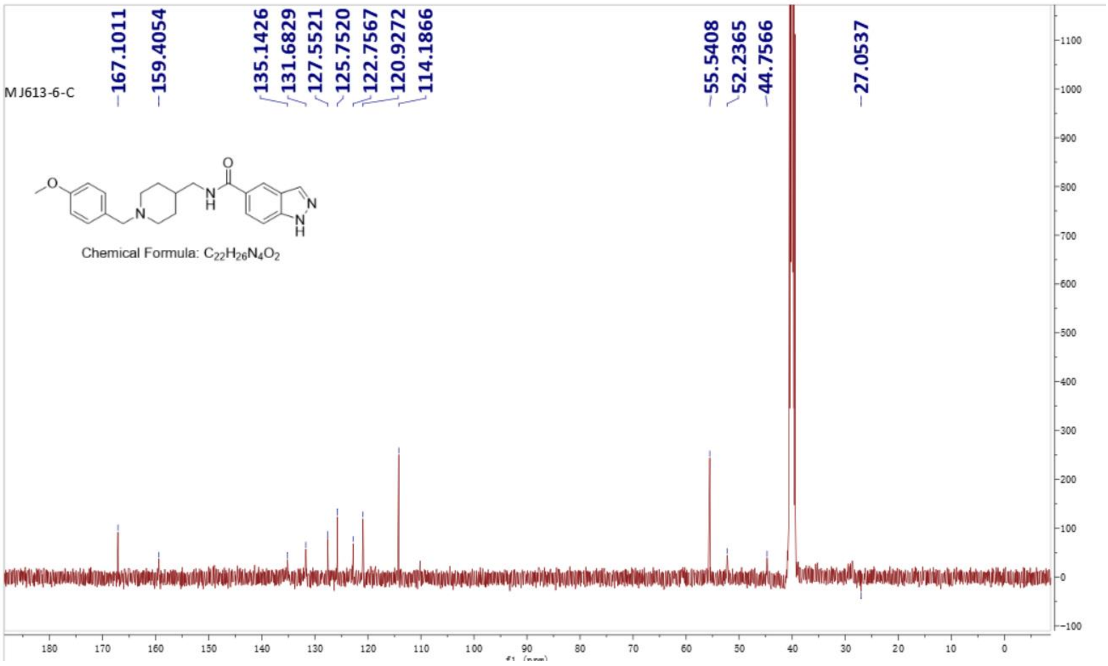

11b.HRMS (ESI)

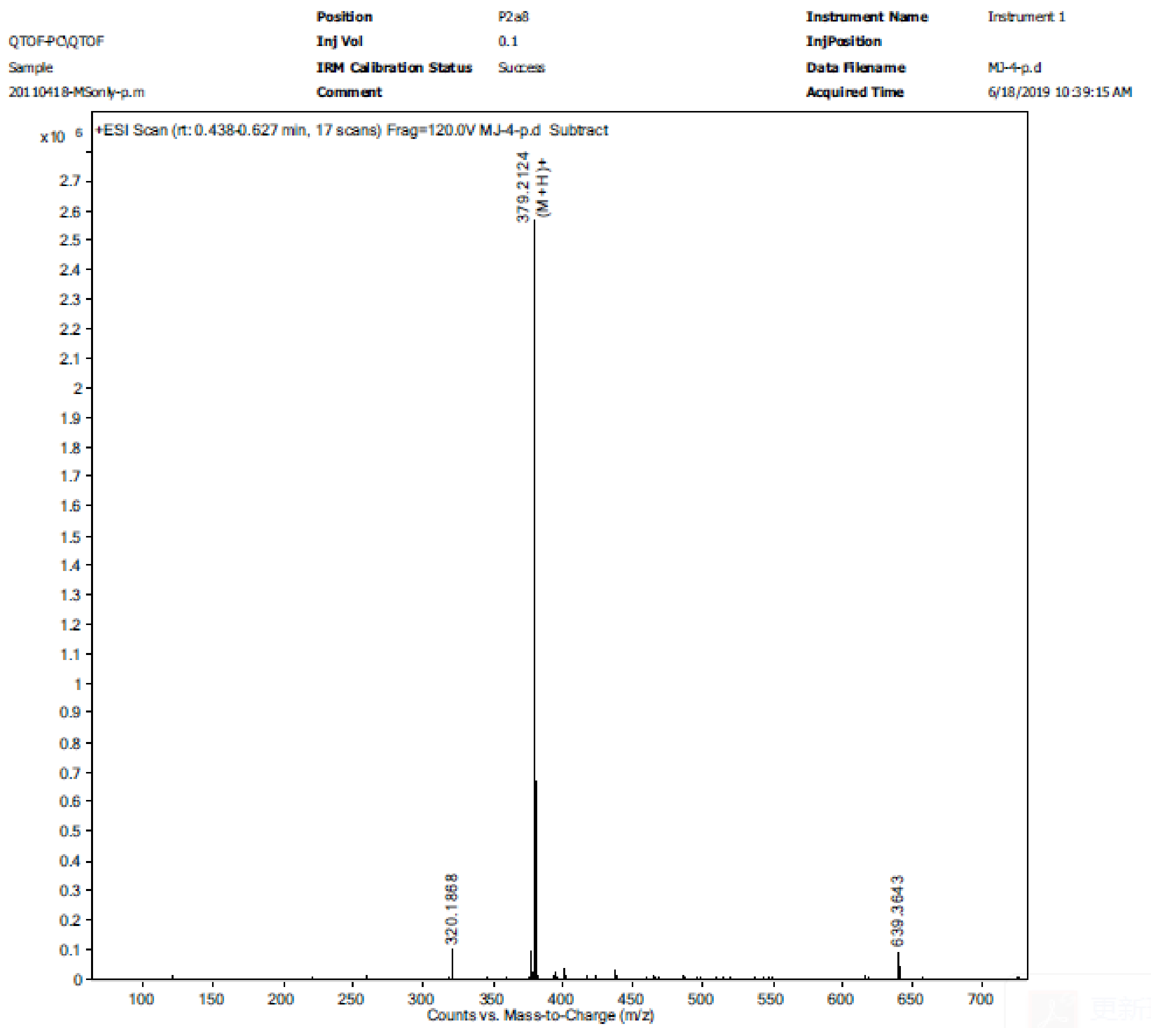

QTOF-PC\QTOF
Sample
20110418-MSonly-p.m
Position
P2a8
Inj Vol
0.1
IRM Calibration Status
Success
Comment
Instrument Name
Instrument 1
InjPosition
Data Filename
MJ-4-p.d
Acquired Time
6/18/2019 10:39:15 AM
+ESI Scan (rt: 0.438-0.627 min, 17 scans) Frag=120.0V MJ-4-p.d Subtract
x10 6
379.2124
(M+H)+
320.1868
639.3643
Counts vs. Mass-to-Charge (m/z)

11c. ^{1}H NMR

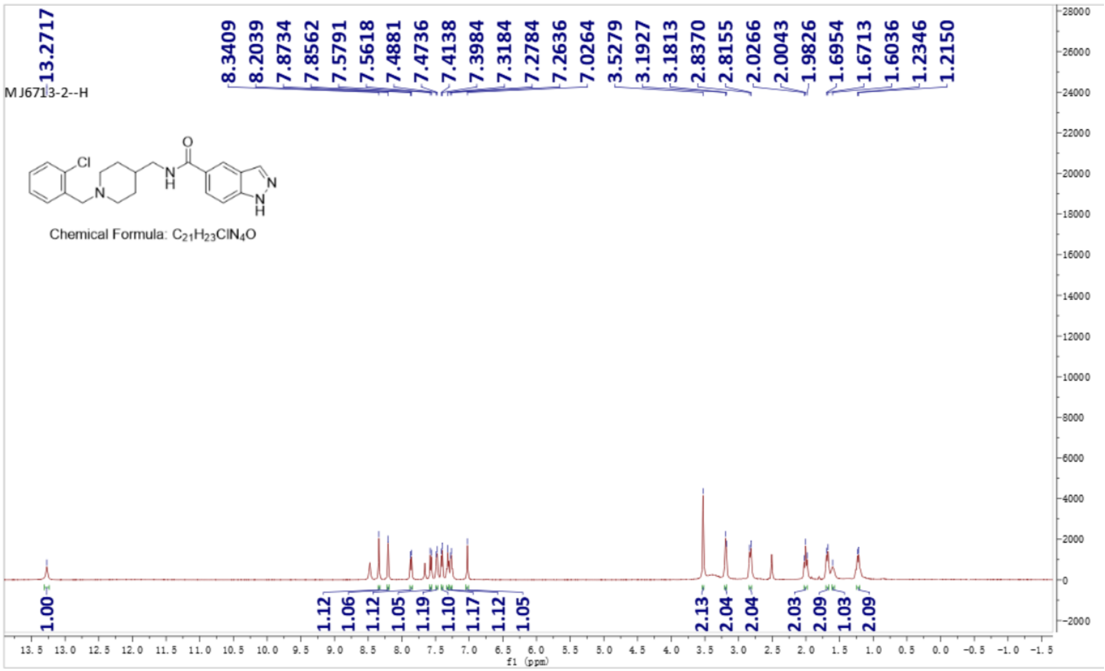

11c.^{13}C NMR

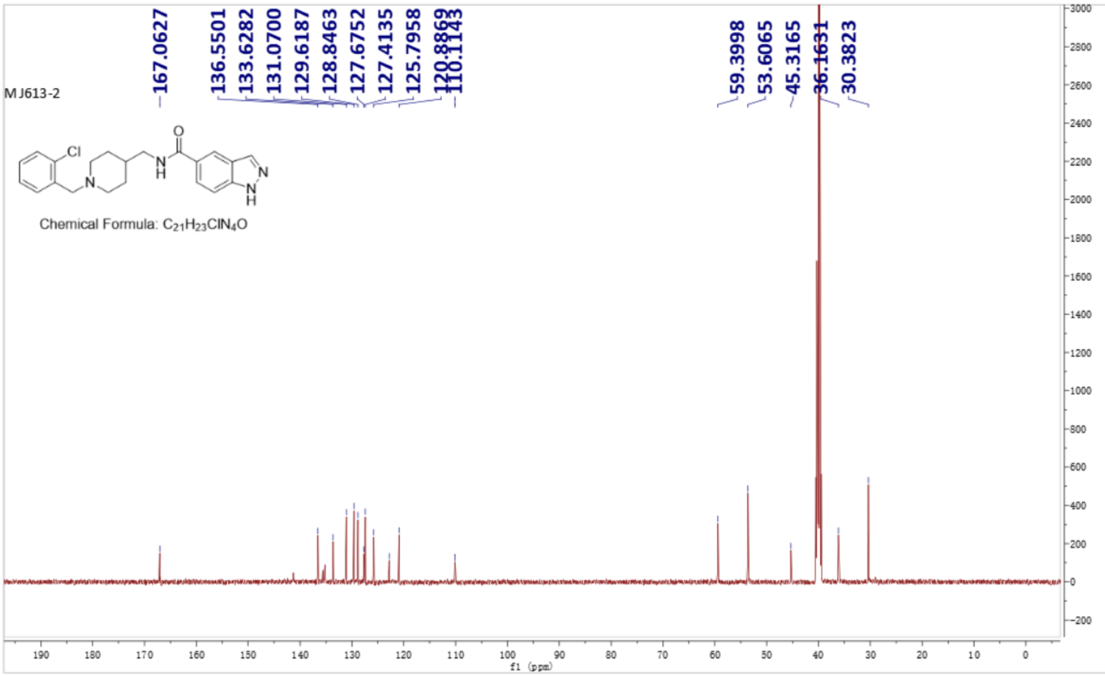

11c.HRMS (ESI)

	**Position**	P2a9	**Instrument Name**	Instrument 1
QTOF-PC\QTOF	**Inj Vol**	0.1	**InjPosition**	
Sample	**IRM Calibration Status**	Success	**Data Filename**	MJ-5-p.d
20110418-MSonly-p.m	**Comment**		**Acquired Time**	6/18/2019 10:44:44 AM

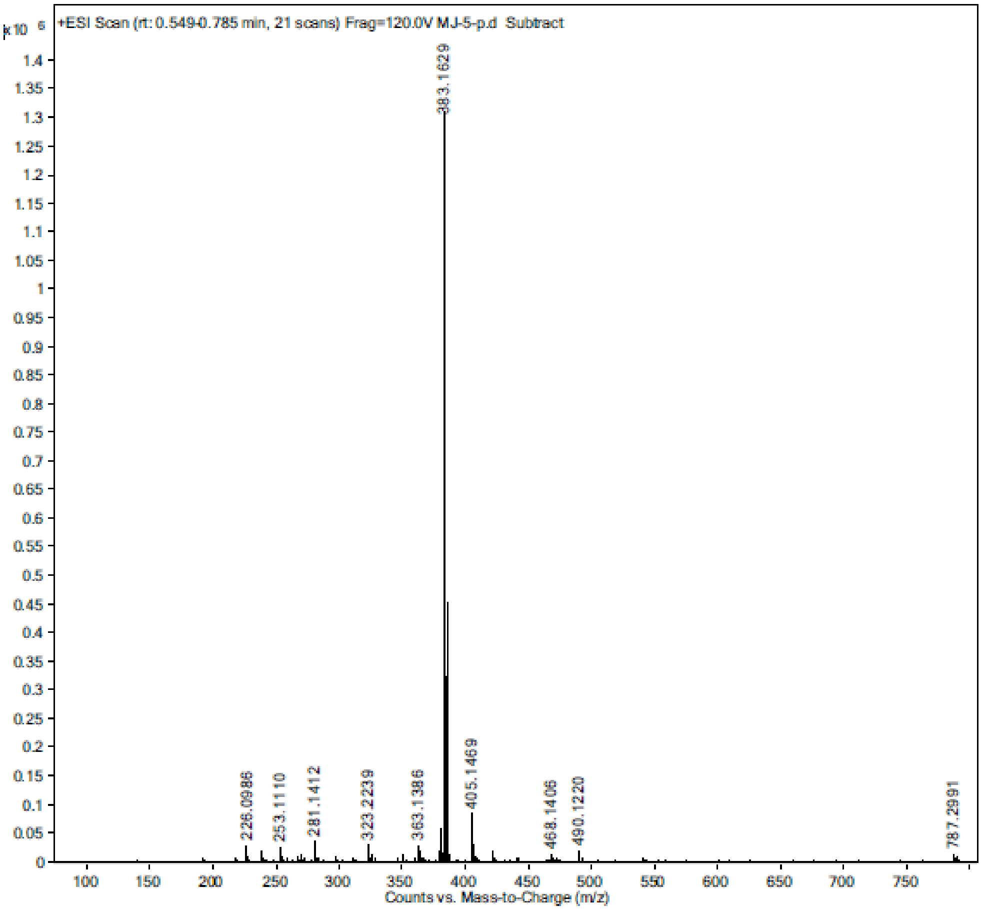

11d.^{1}H NMR

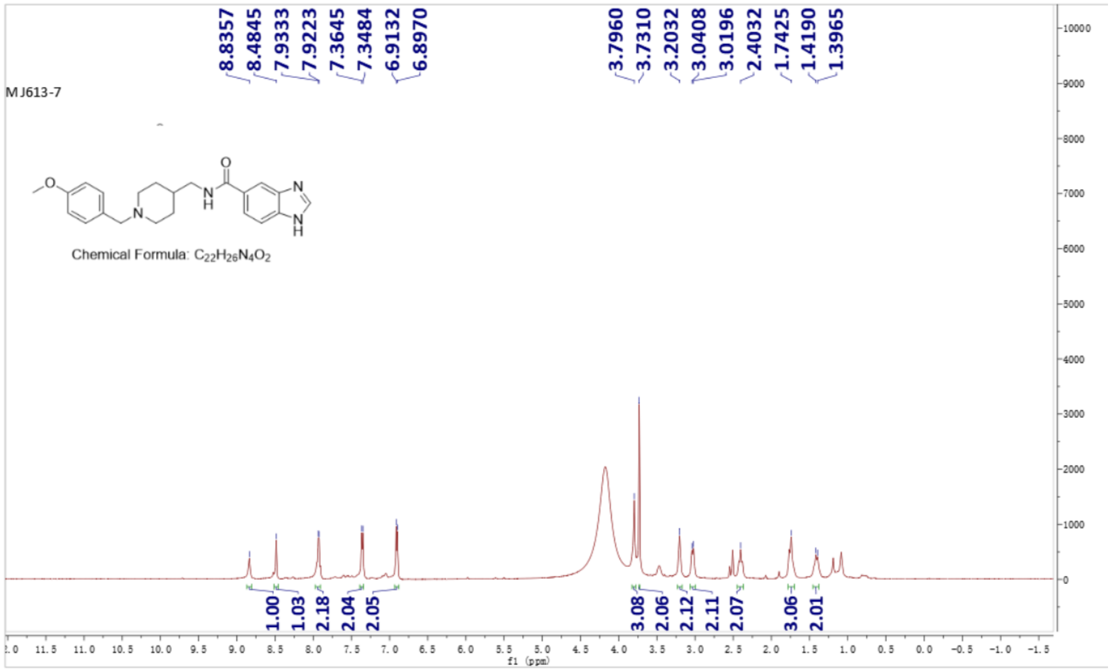

11d.^{13}C NMR

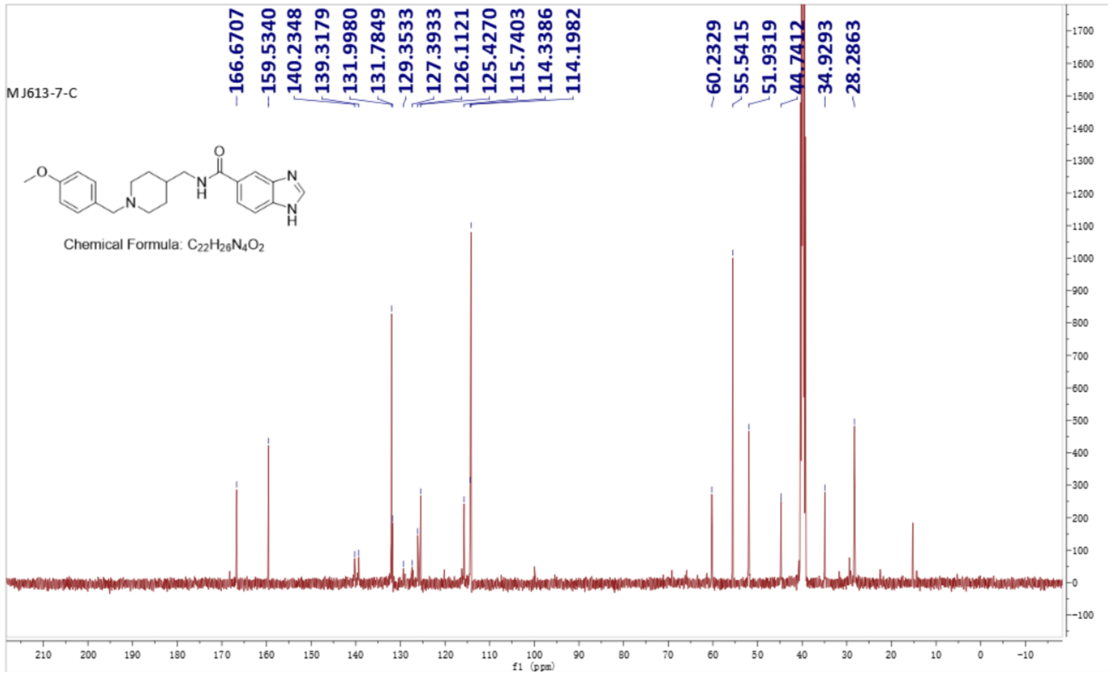

11d.HRMS (ESI)

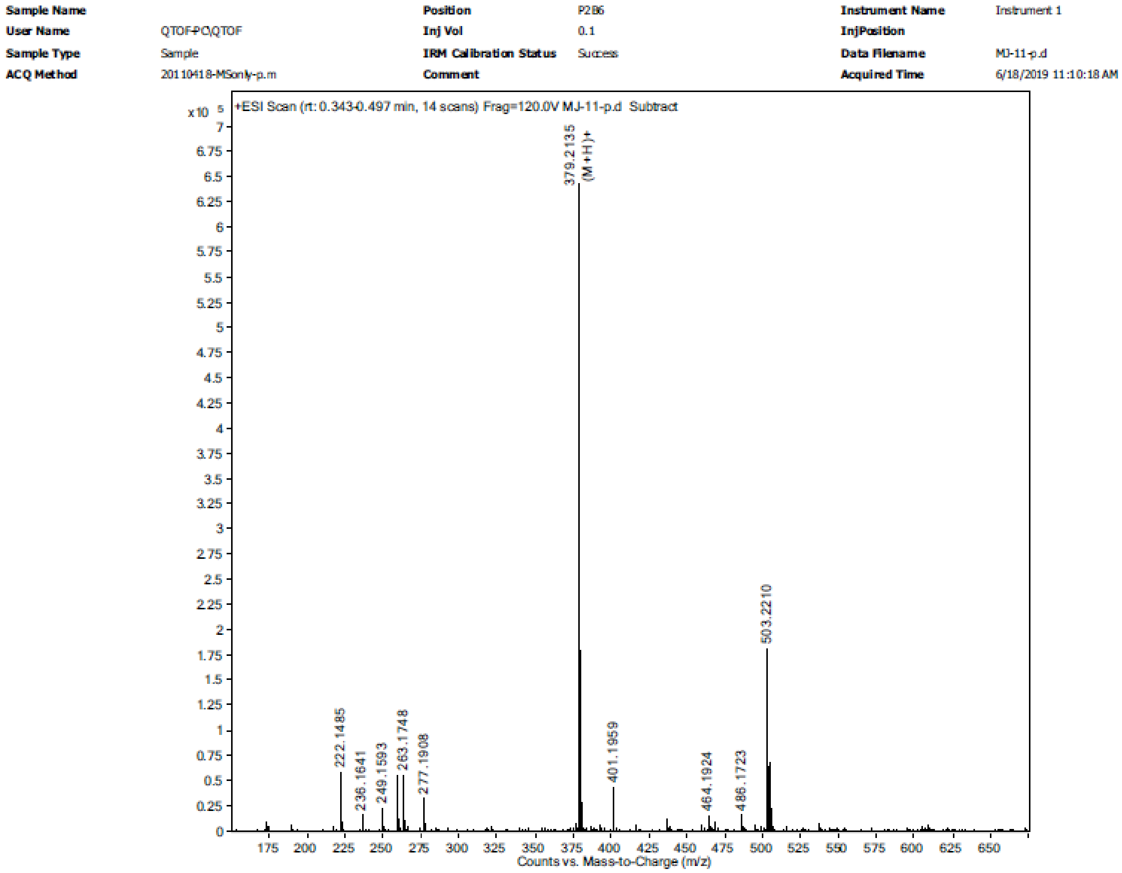

11e.^{1}H NMR

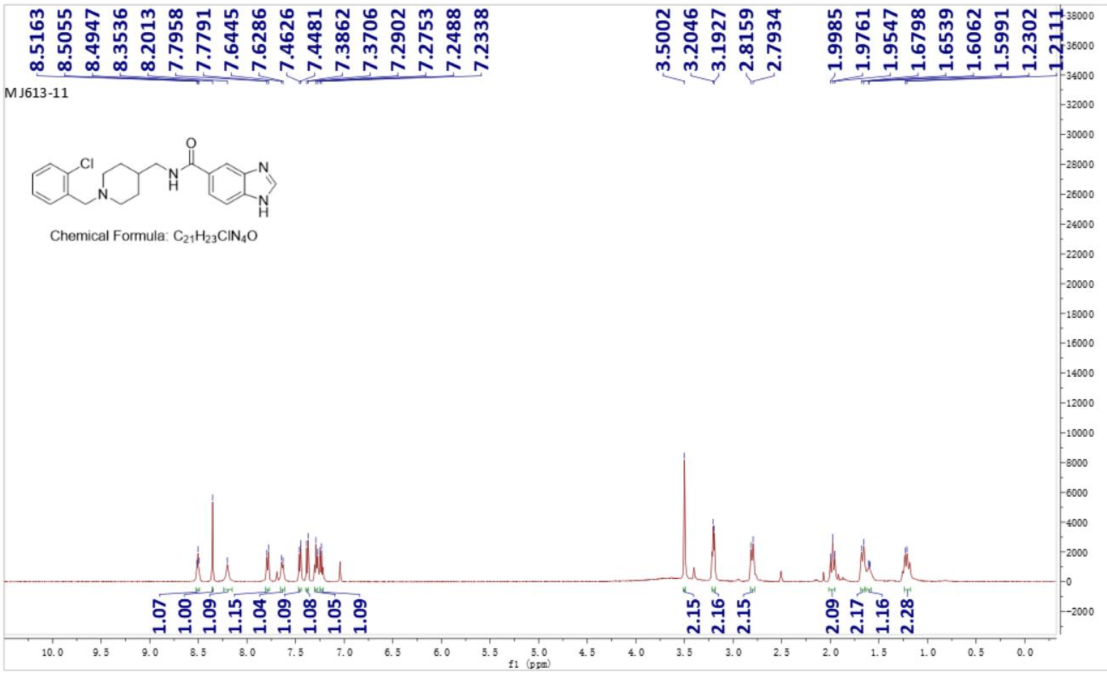

11e.^{13}C NMR

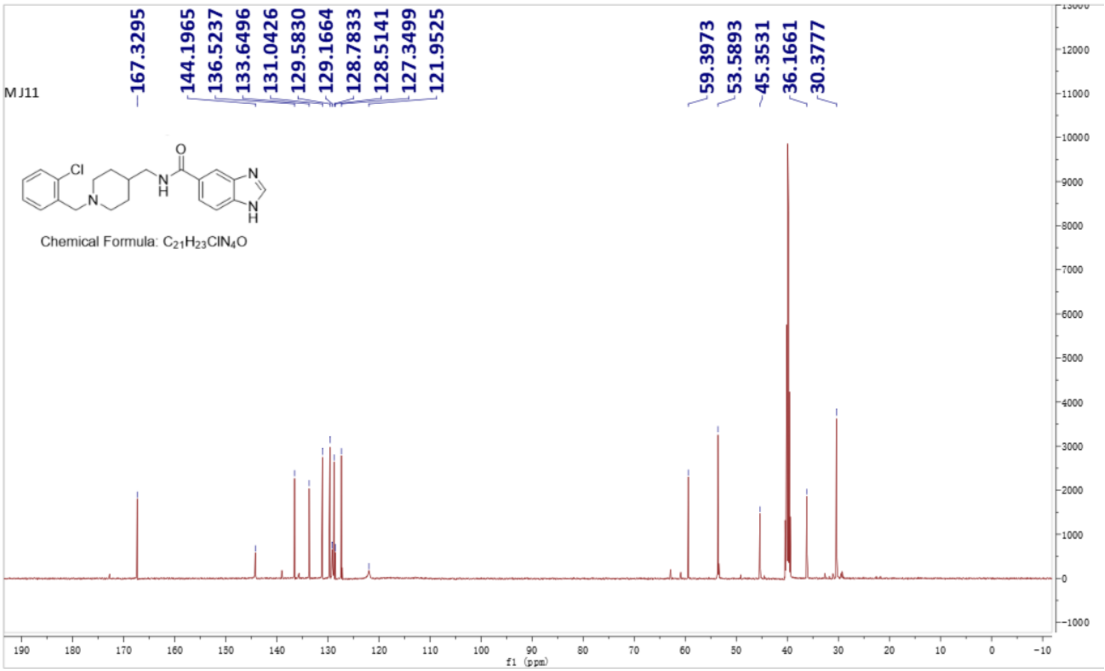

MJ11
167.3295
144.1965
136.5237
133.6496
131.0426
129.5830
129.1664
128.7833
128.5141
127.3499
121.9525
59.3973
53.5893
45.3531
36.1661
30.3777
Chemical Formula: C21H23ClN4O
f1 (ppm)

11e.HRMS (ESI)

	**Position**	P2a5	**Instrument Name**	Instrument 1
QTOF-PC\QTOF	**Inj Vol**	0.01	**InjPosition**	
Sample	**IRM Calibration Status**	Success	**Data Filename**	MJ-1-p.d
20110418-MSonly-p.m	**Comment**		**Acquired Time**	6/18/2019 10:24:29 AM

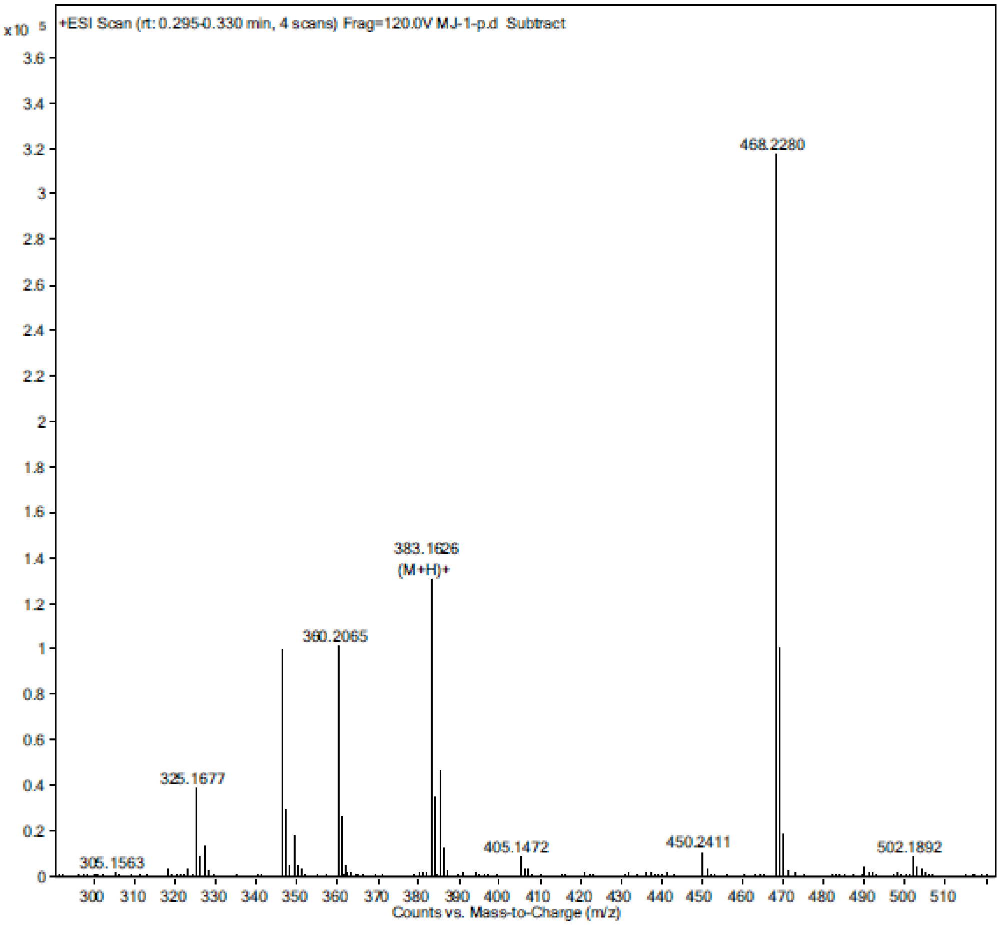

11f.^{1}H NMR

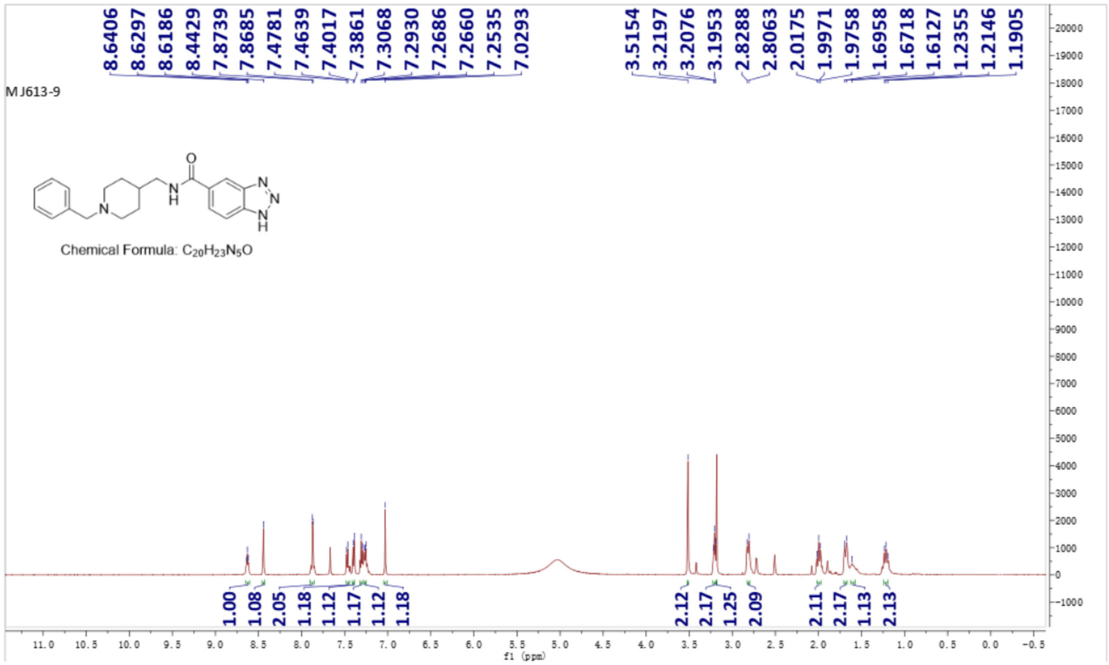

11f.^{13}C NMR

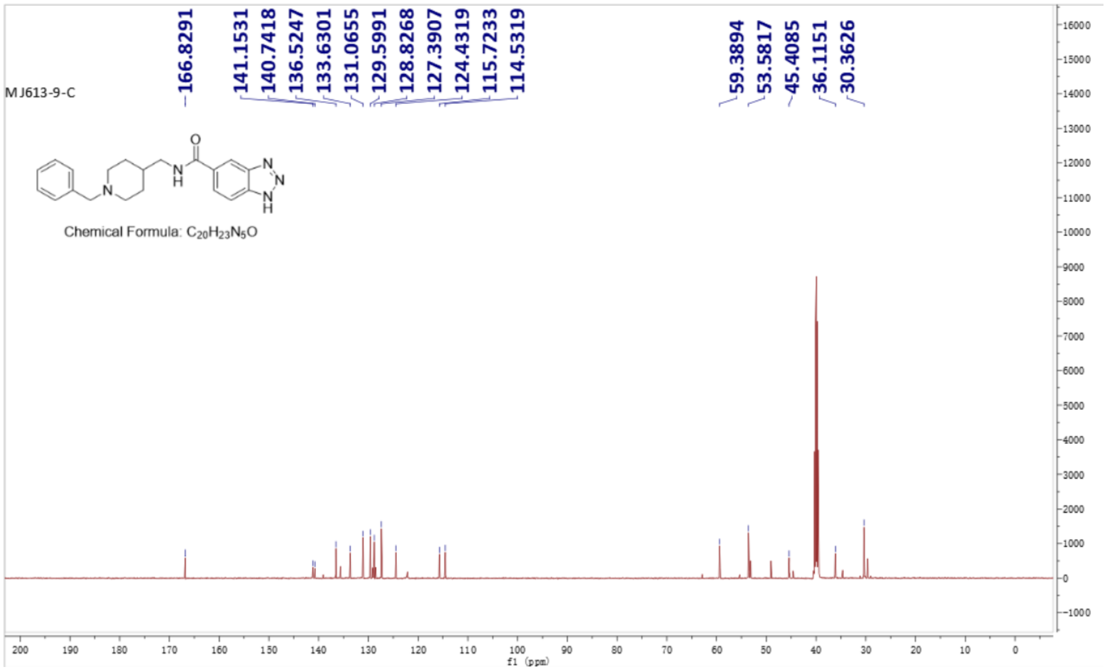

11f.HRMS (ESI)

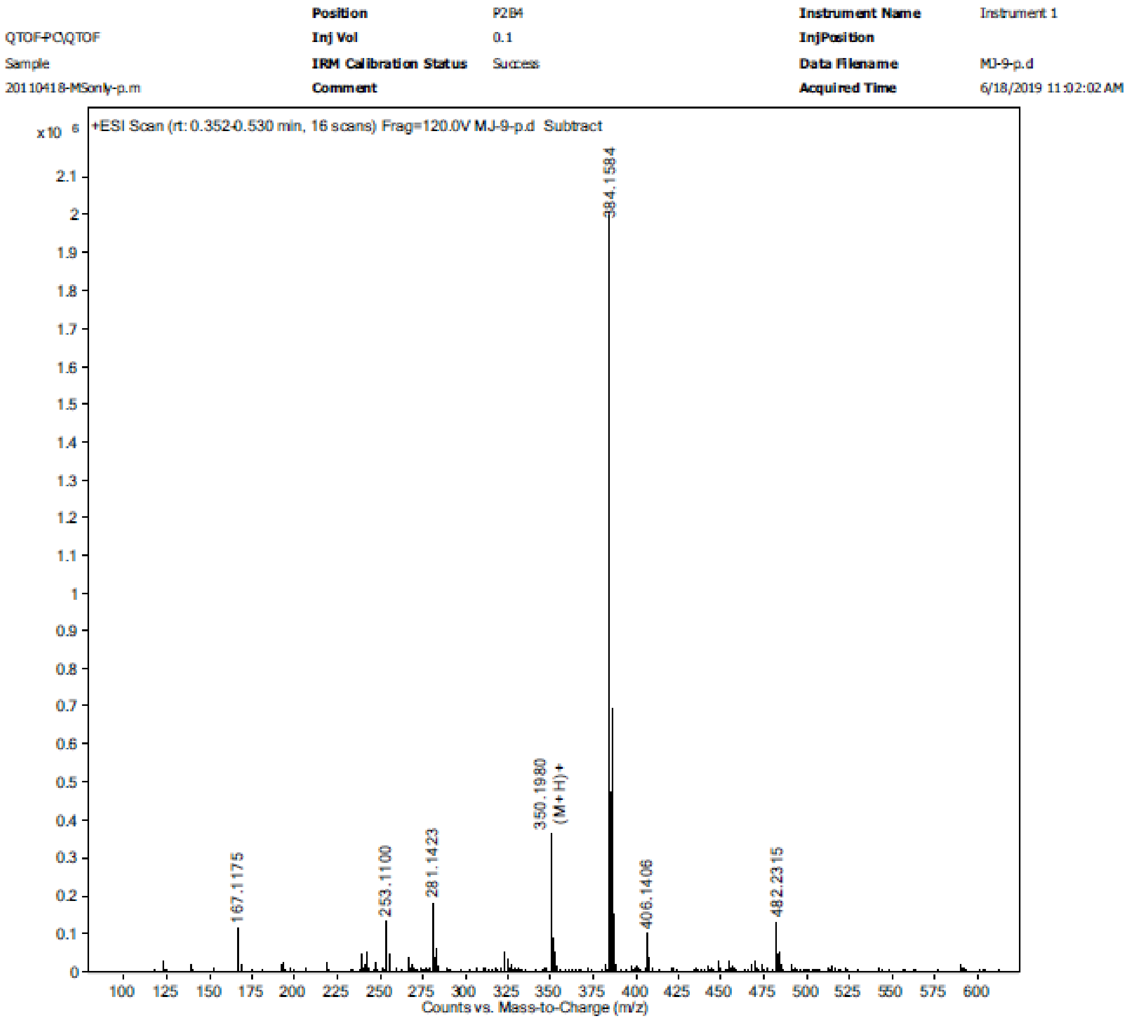
Position P2B4
QTOF-PC\QTOF
Inj Vol 0.1
Sample
IRM Calibration Status Success
20110418-MSonly-p.m
Comment
Instrument Name Instrument 1
InjPosition
Data Filename MJ-9-p.d
Acquired Time 6/18/2019 11:02:02 AM
x10 6
+ESI Scan (rt: 0.352-0.530 min, 16 scans) Frag=120.0V MJ-9-p.d Subtract
384.1584
350.1980
(M+H)+
167.1175
253.1100
281.1423
406.1406
482.2315
Counts vs. Mass-to-Charge (m/z)

15a. ^{1}H NMR

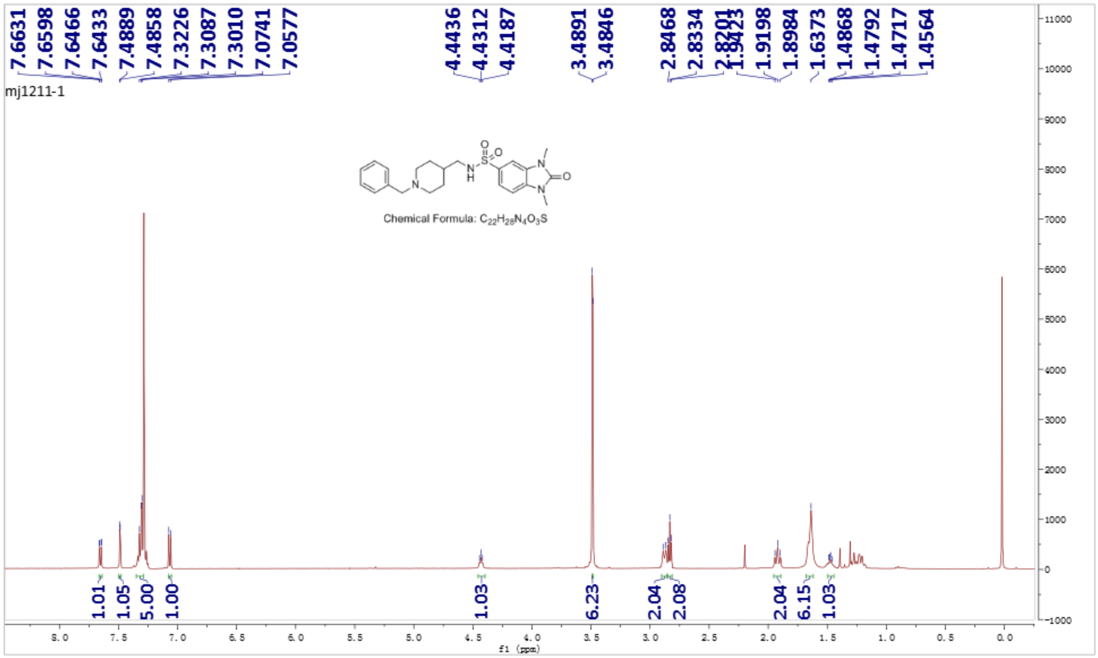

15a. ^{13}C NMR

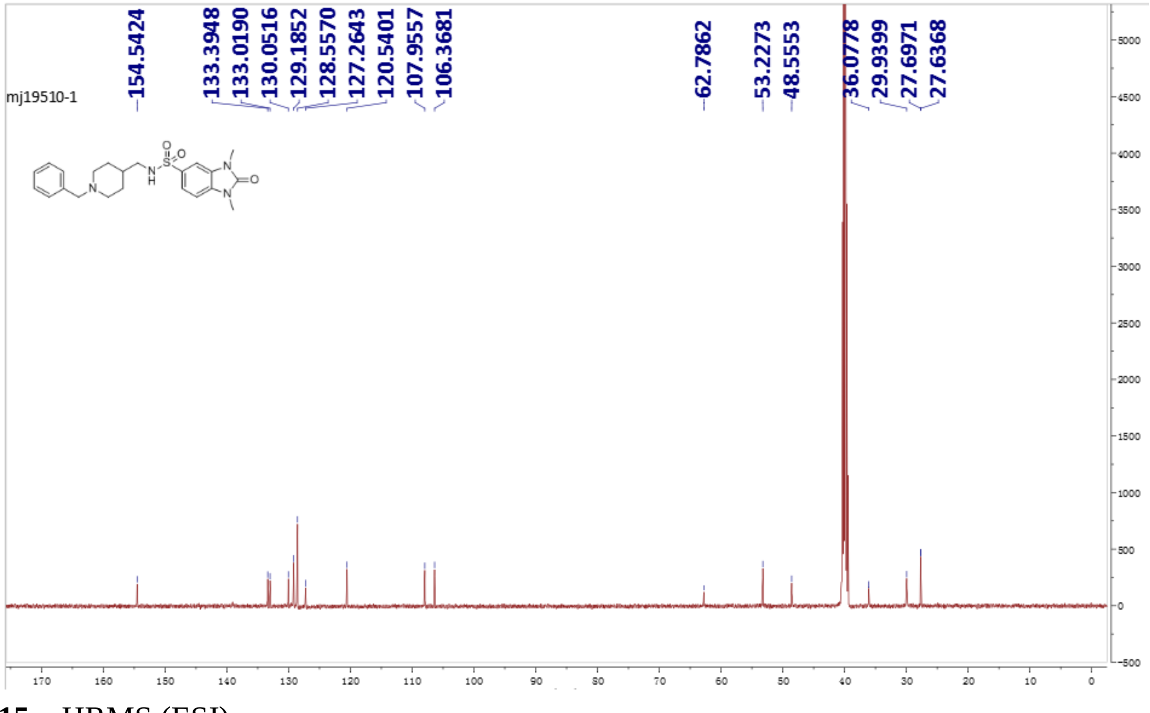

15a. HRMS (ESI)

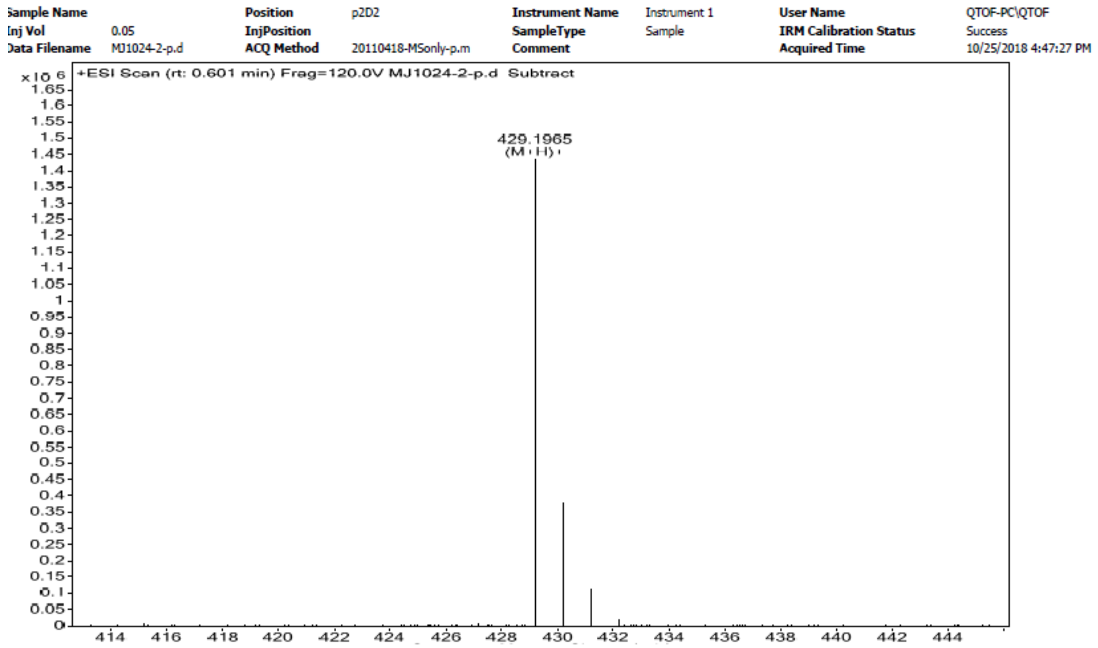

15b. ^{1}H NMR

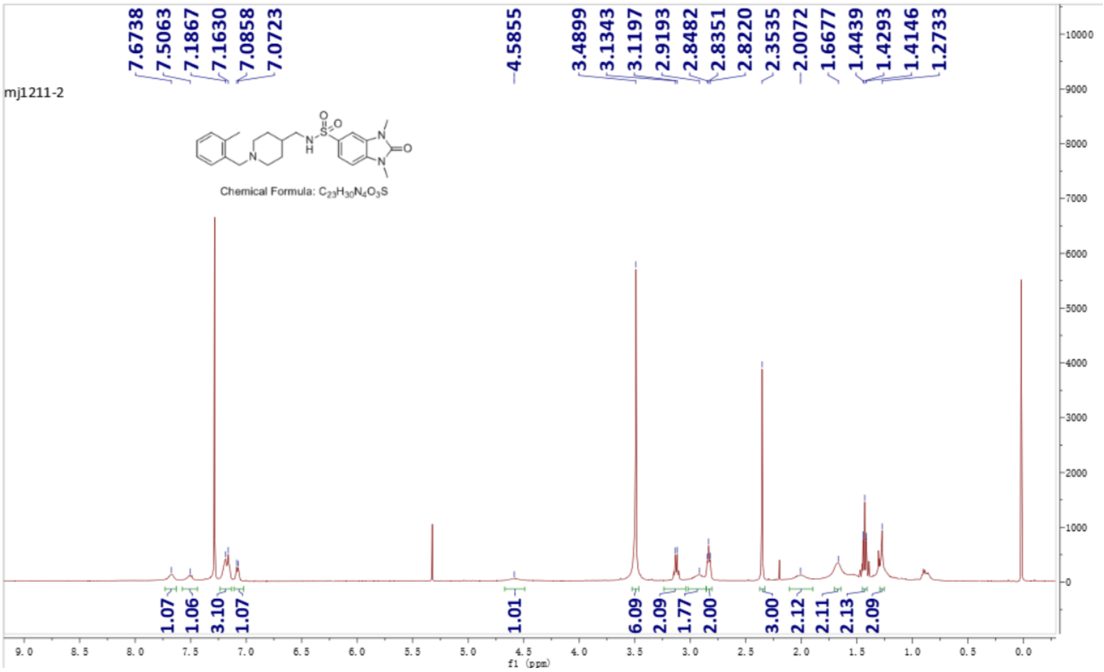

15b. ^{13}C NMR

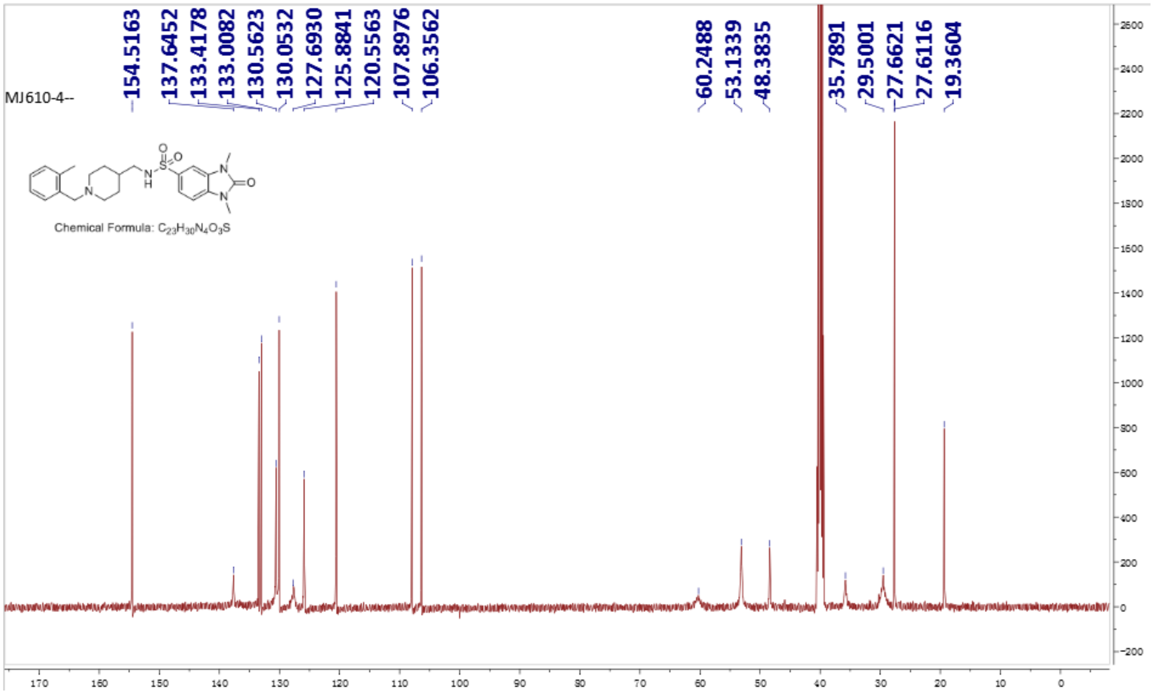

15b. HRMS (ESI)

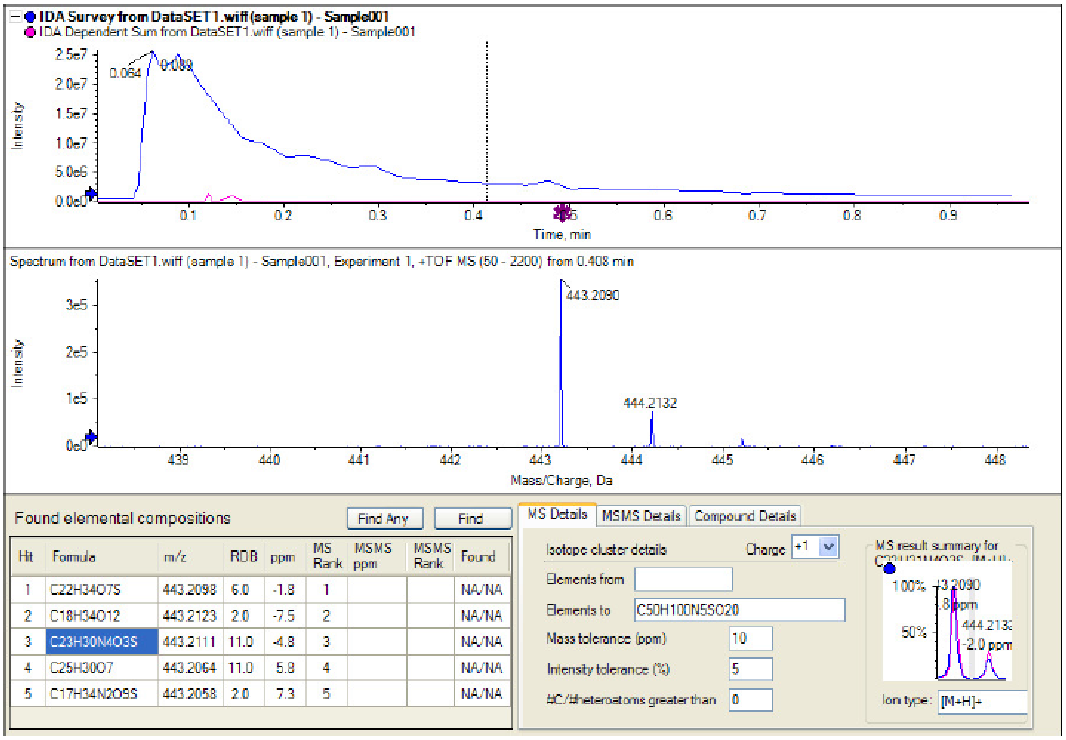

15c. ^{1}H NMR

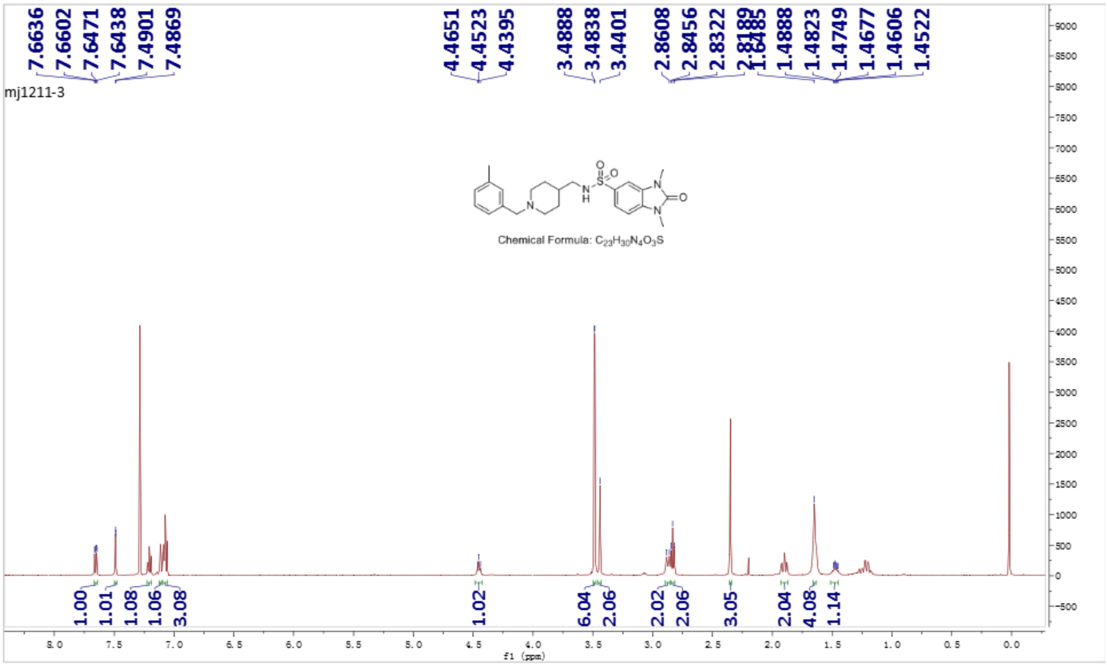

15c. ^{13}C NMR

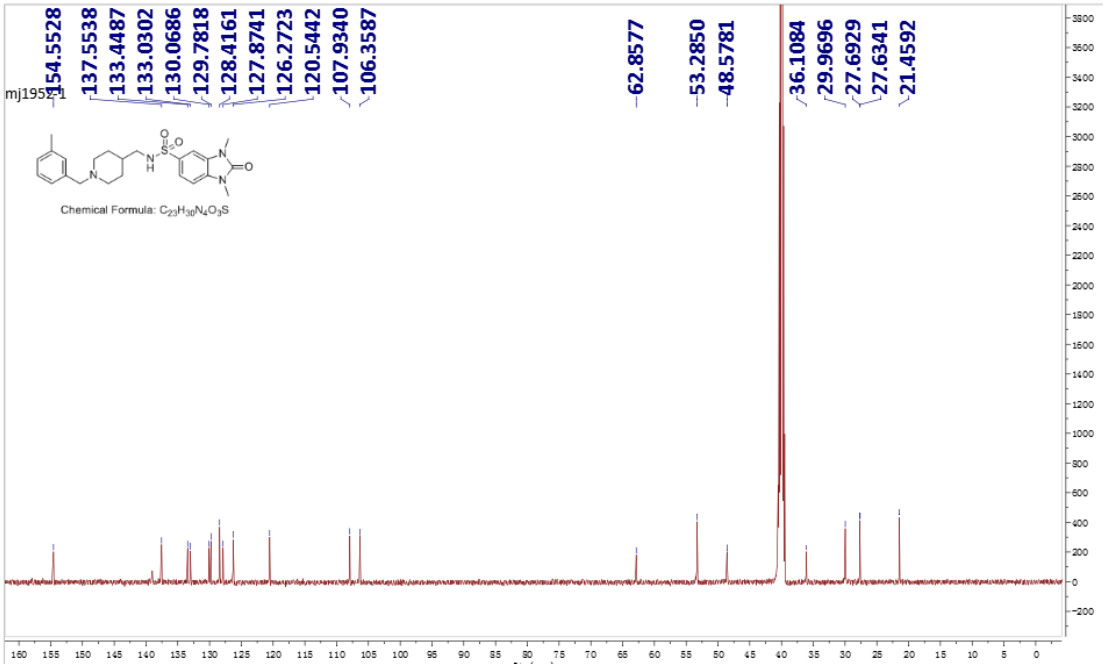

15c. HRMS (ESI)

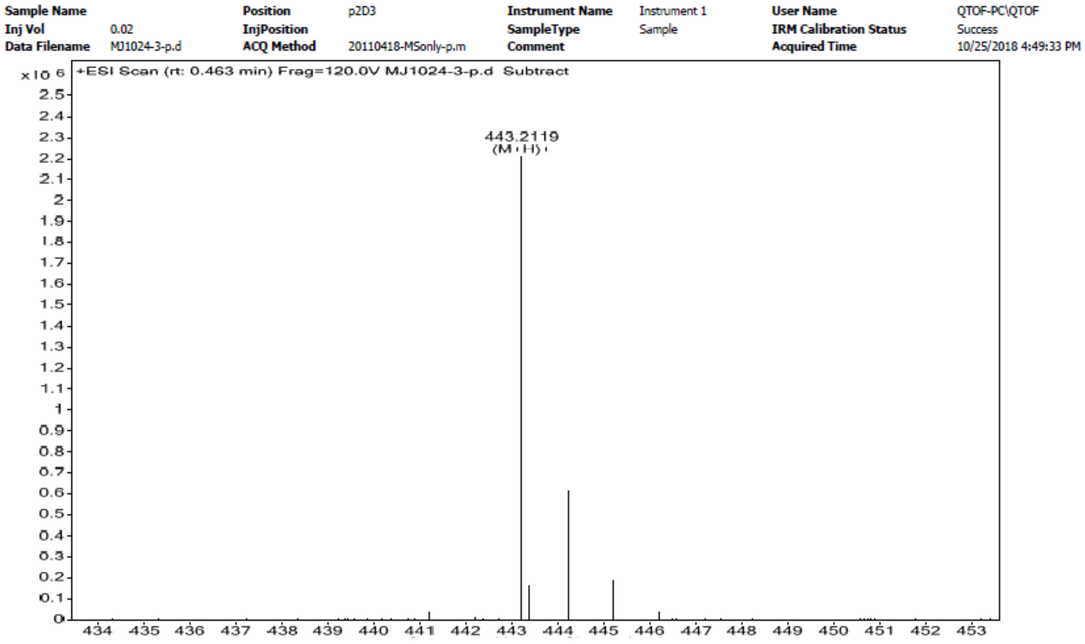

15d. ^{1}H NMR

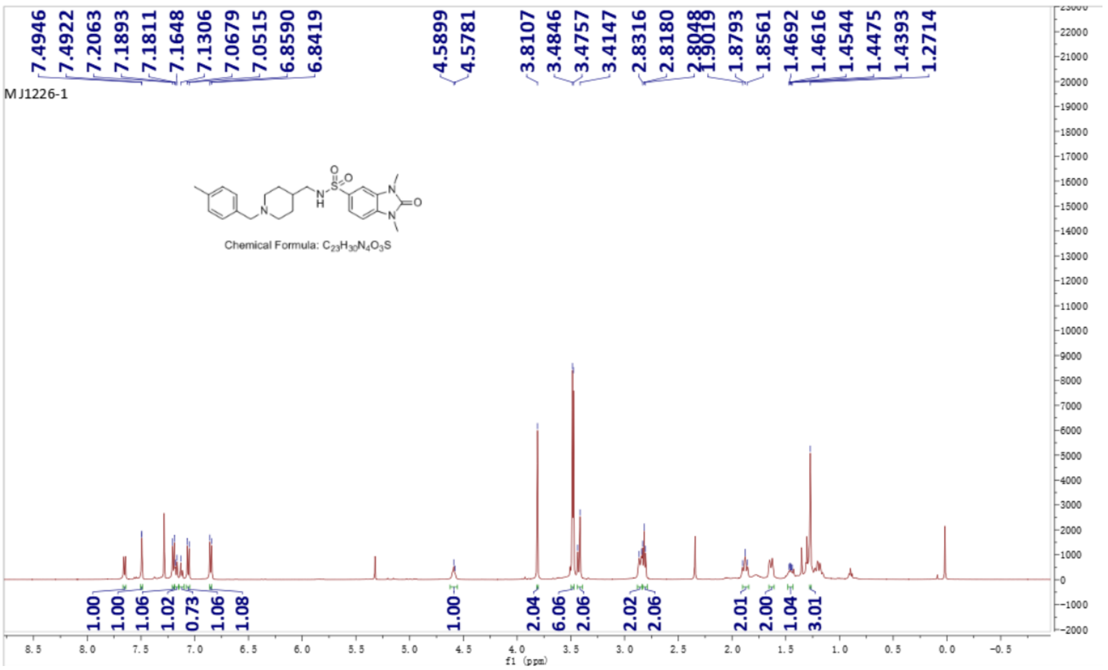

15d. ^{13}C NMR

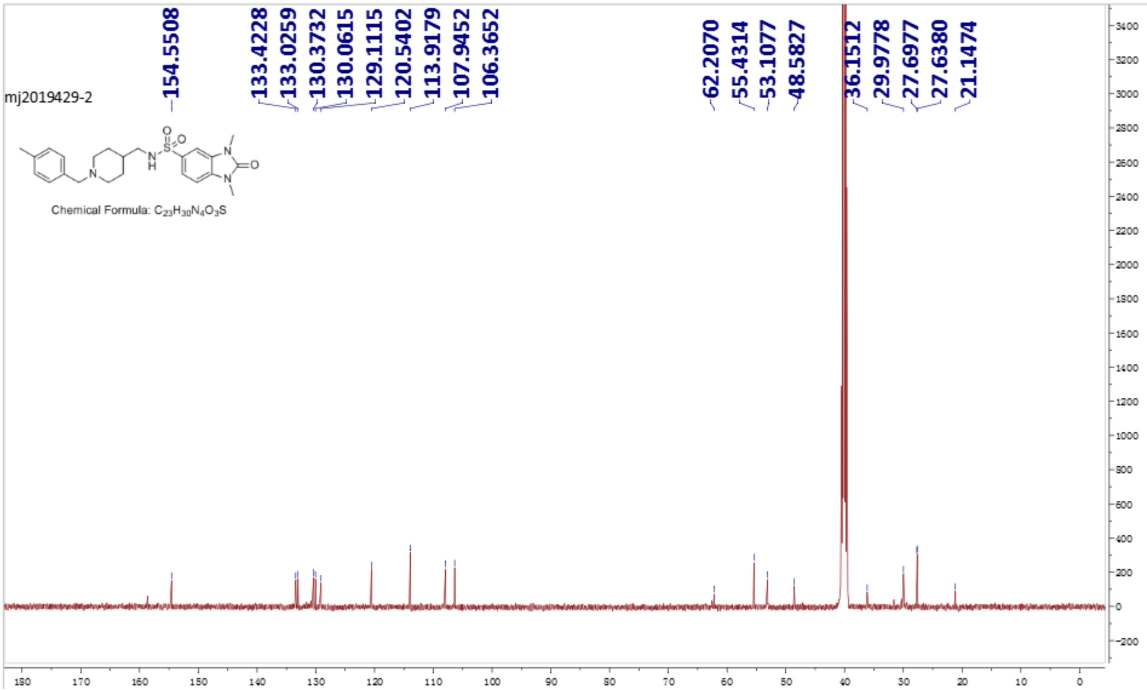

15d. HRMS (ESI)

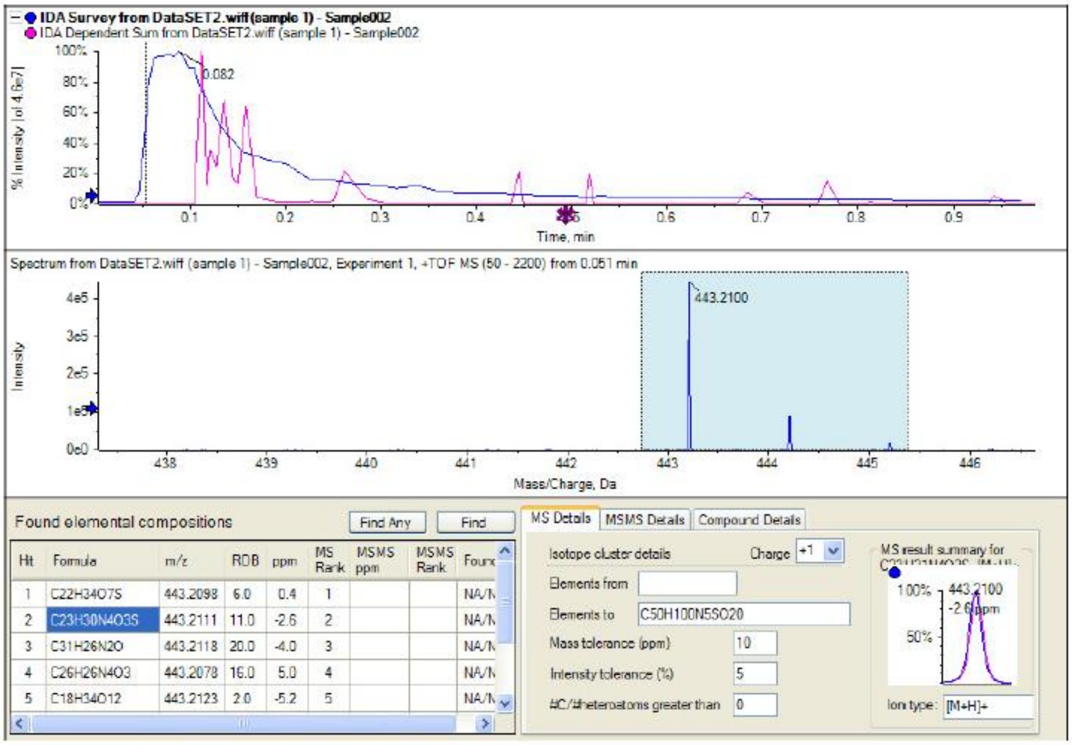

Hit	Formula	m/z	RDB	ppm	MS Rank	MSMS ppm	MSMS Rank	Found
1	C22H34O7S	443.2098	6.0	0.4	1			NA/N
2	C23H30N4O3S	443.2111	11.0	-2.6	2			NA/N
3	C31H26N2O	443.2118	20.0	-4.0	3			NA/N
4	C26H26N4O3	443.2078	16.0	5.0	4			NA/N
5	C18H34O12	443.2123	2.0	-5.2	5			NA/N

15e. ^{1}H NMR

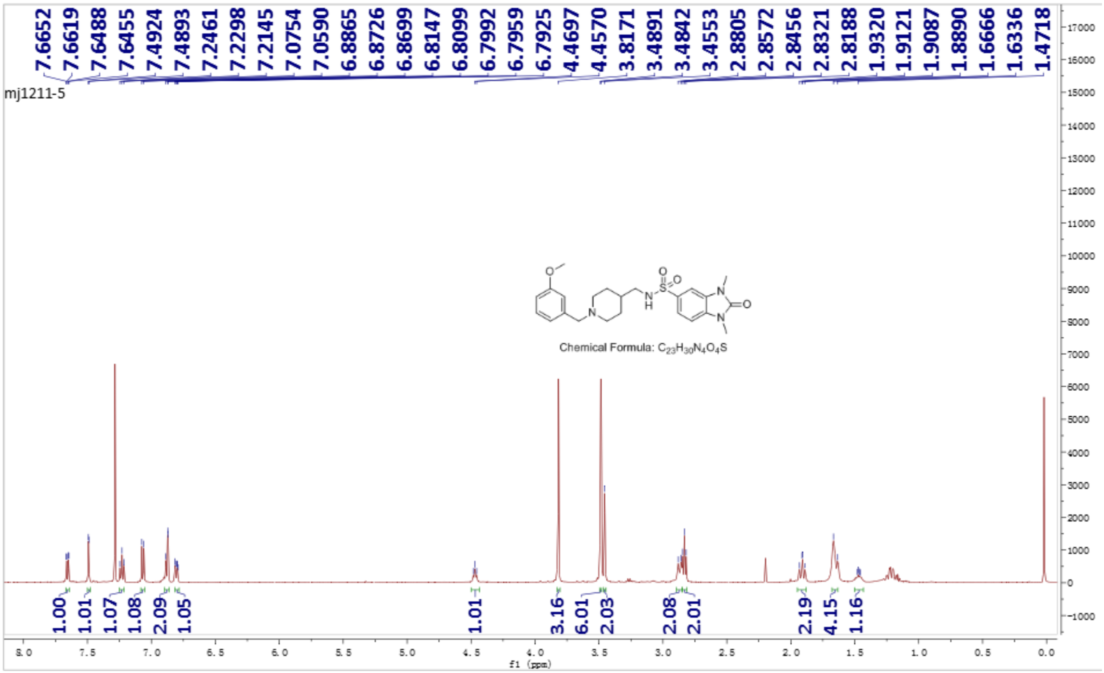

15e. ^{13}C NMR

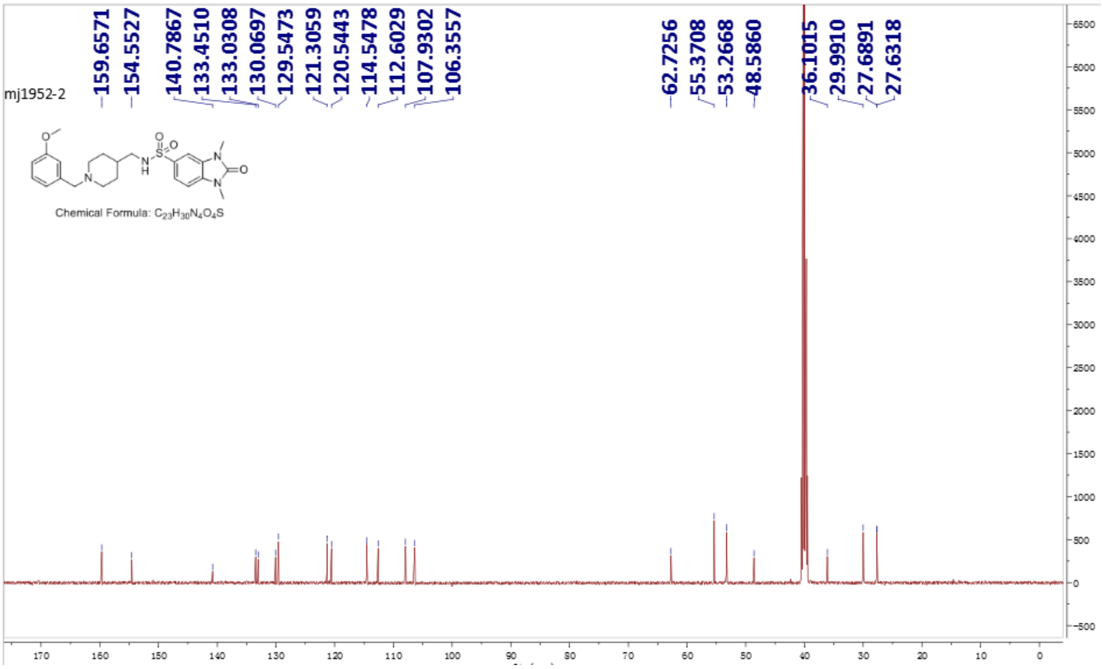

15e. HRMS (ESI)

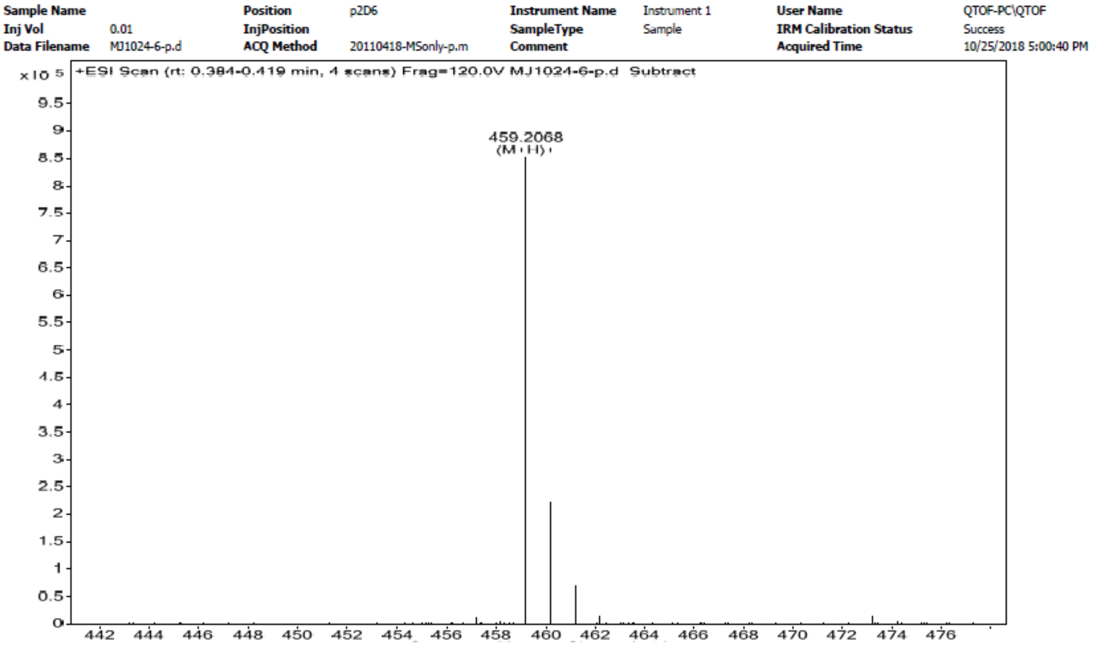

15f .^{1}H NMR

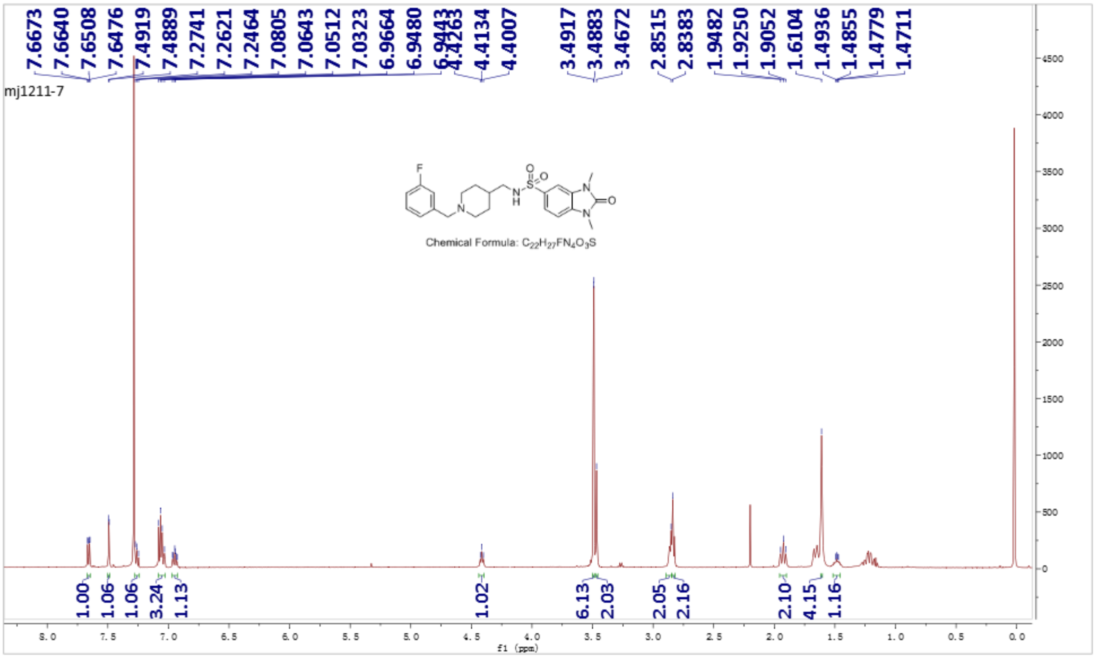

15f. ^{13}C NMR

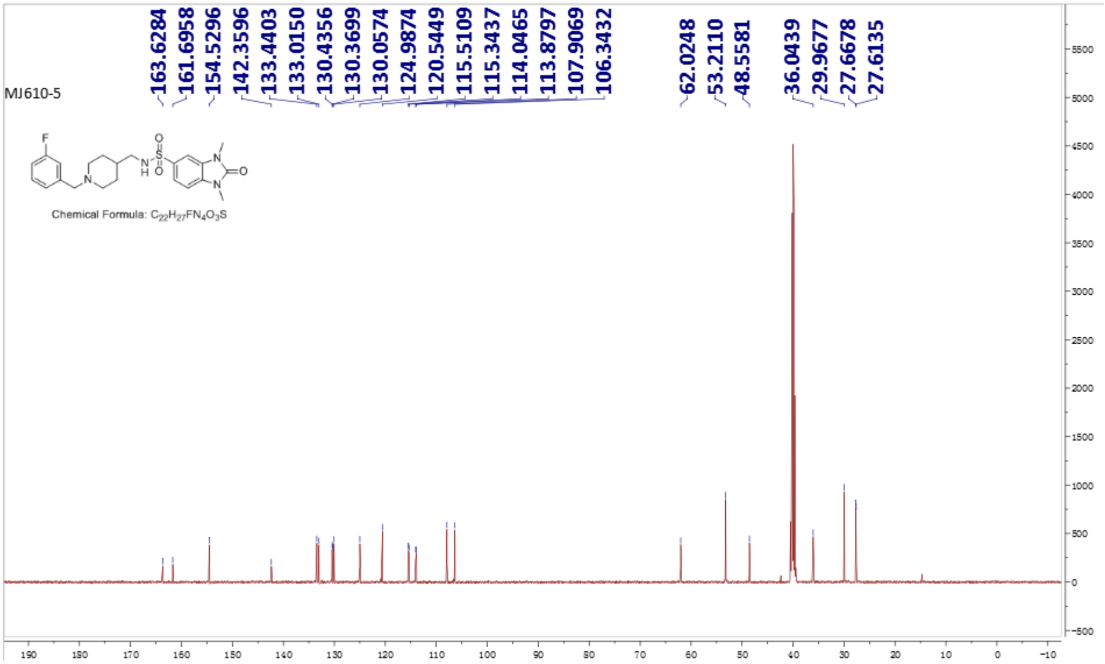

15f .HRMS (ESI)

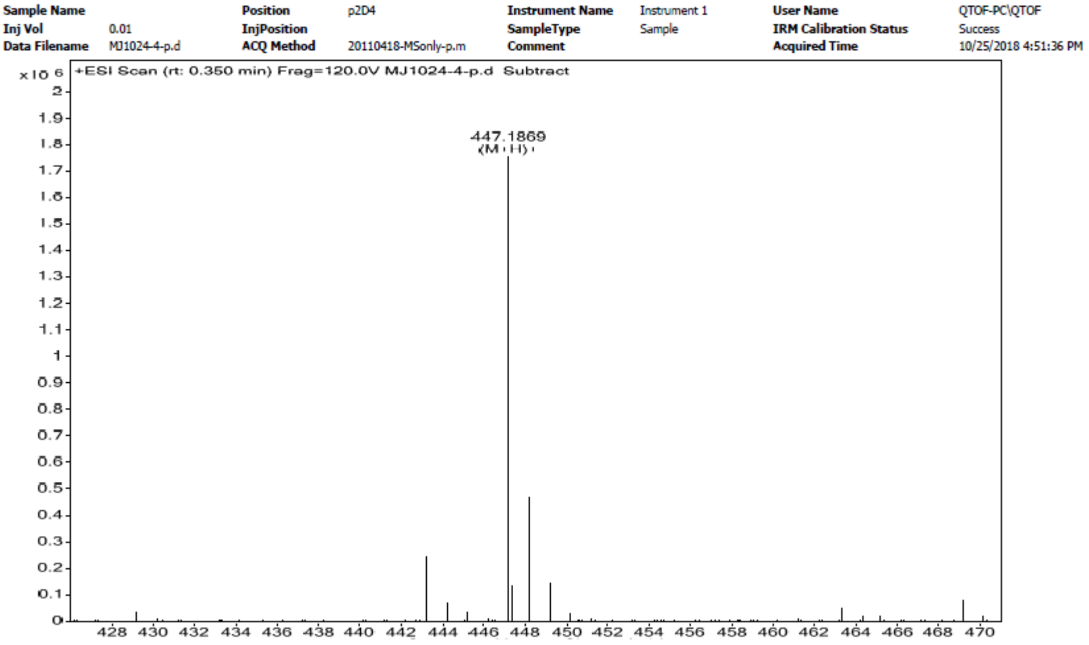

15g.^{1}H NMR

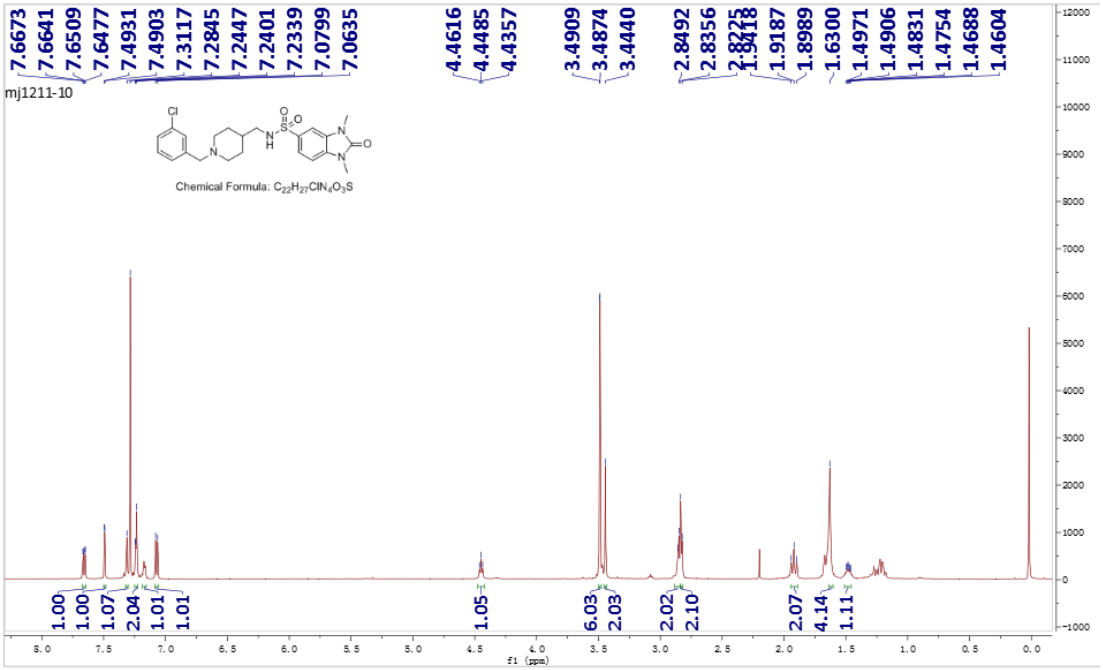

15g. ^{13}C NMR

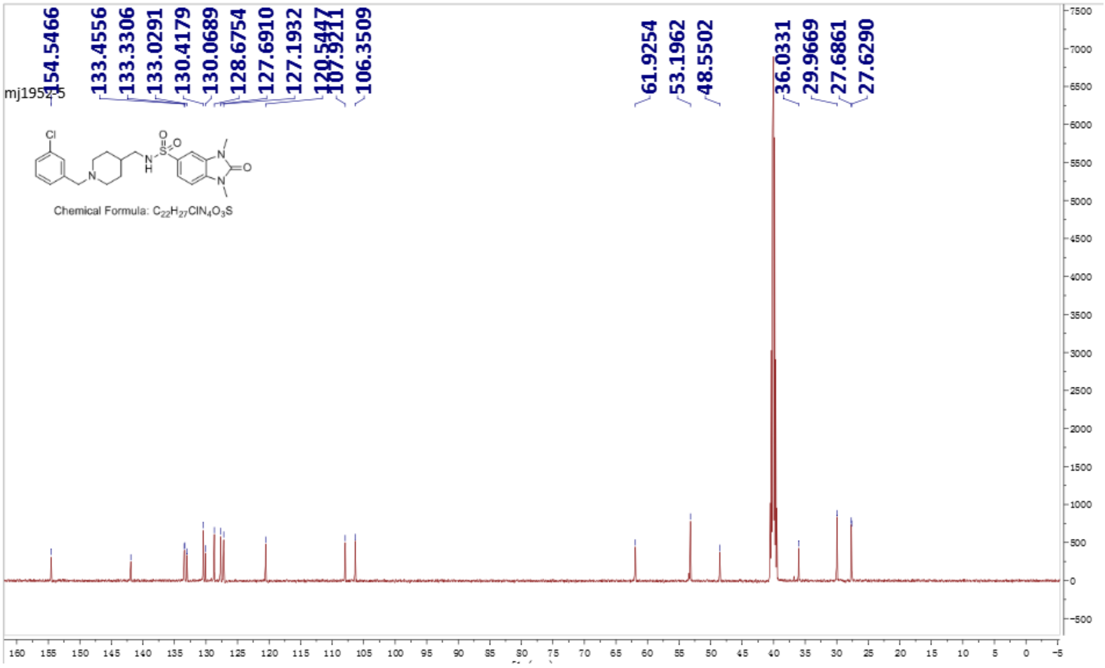

15g. HRMS (ESI)

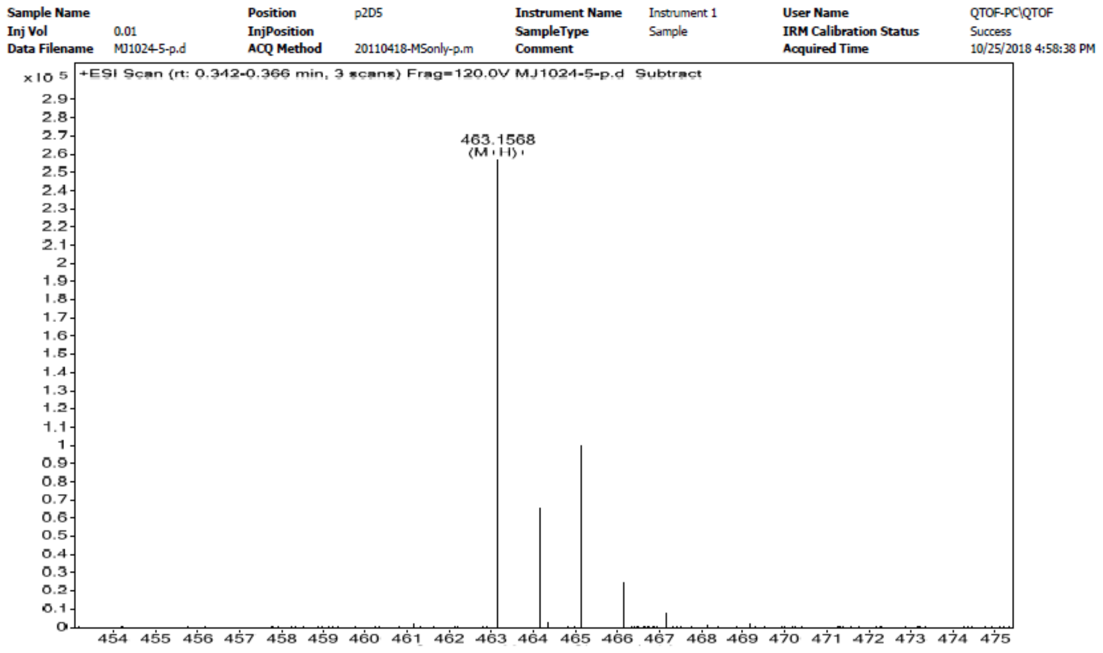

15h. ^{1}H NMR

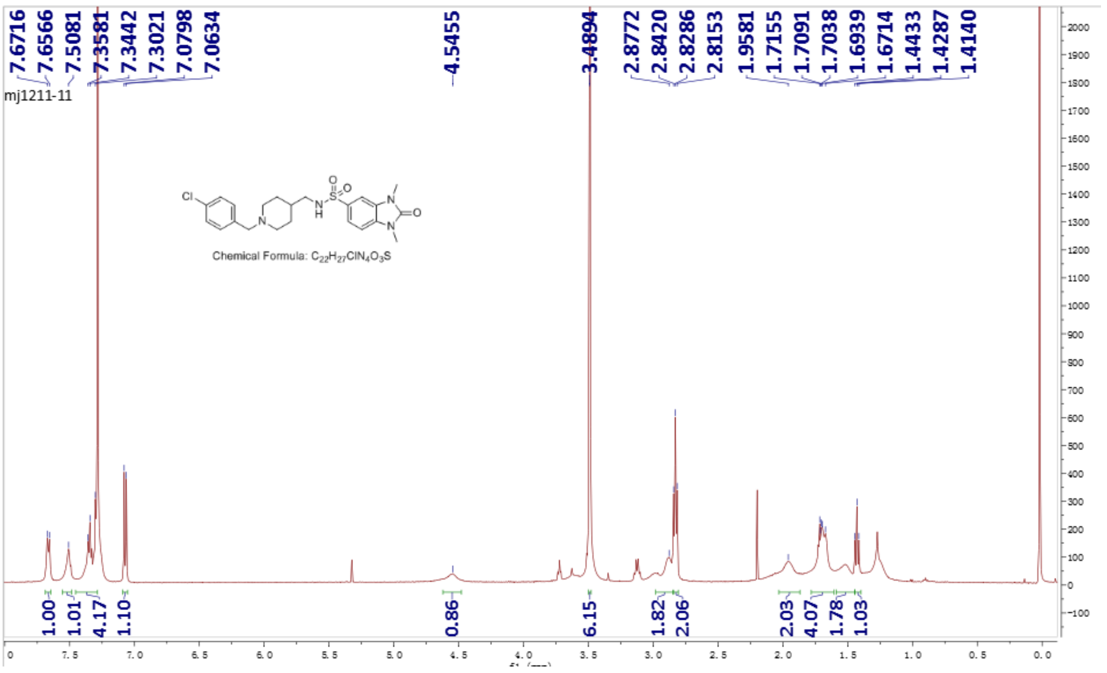

15h. ^{13}C NMR

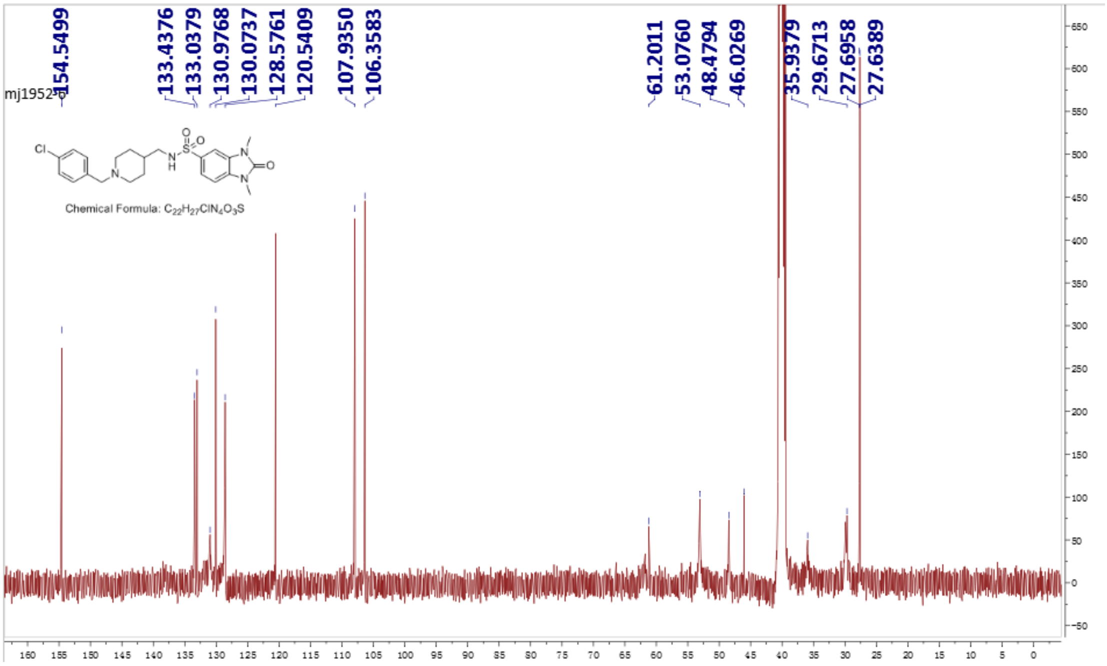

15h.HRMS (ESI)

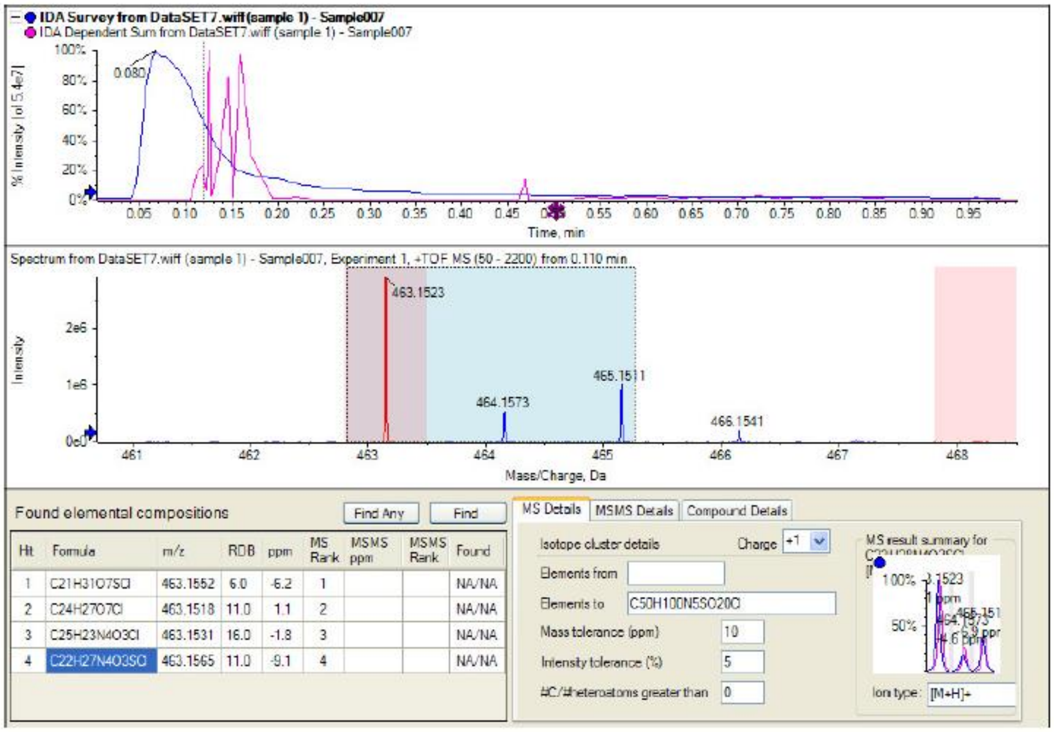

15i .^{1}H NMR

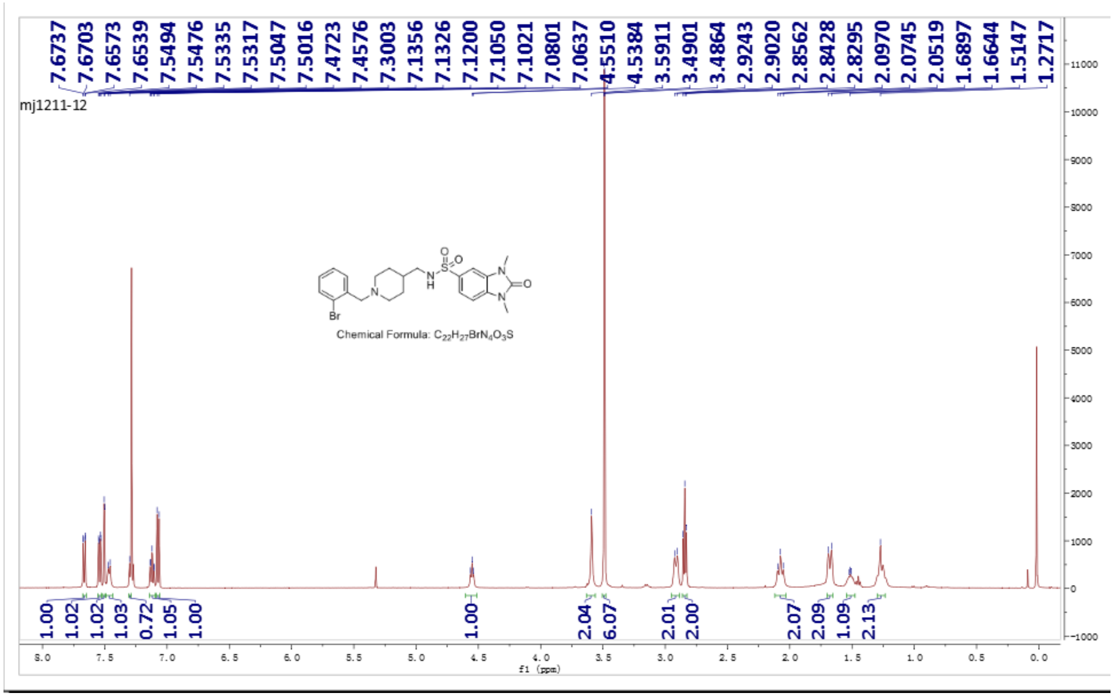

15i. ^{13}C NMR

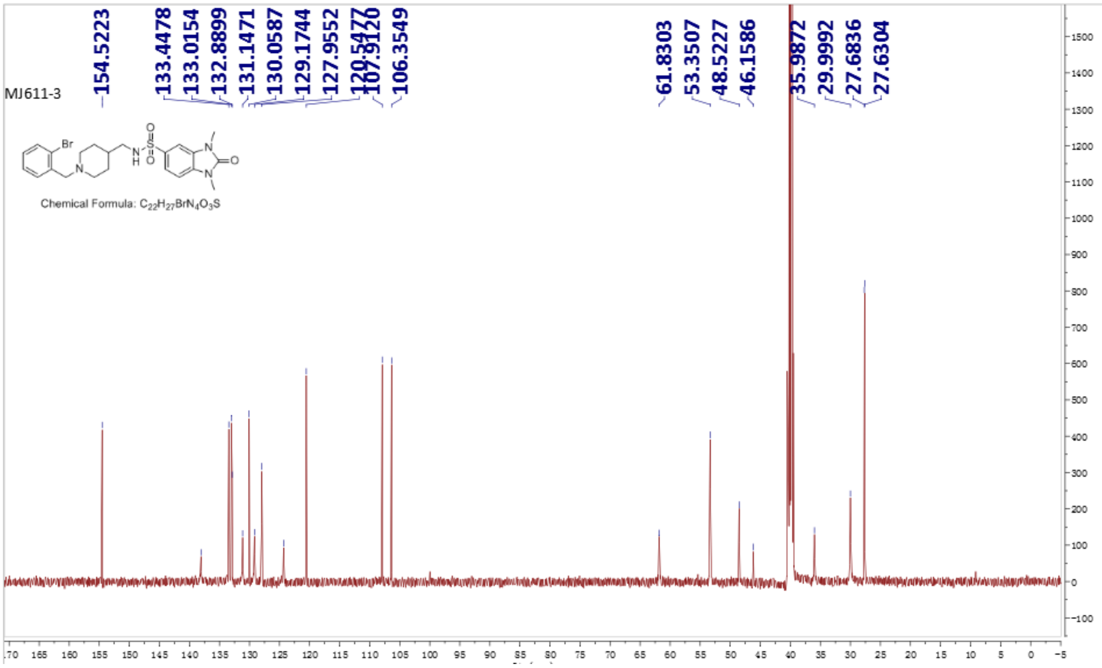

15i.HRMS (ESI)

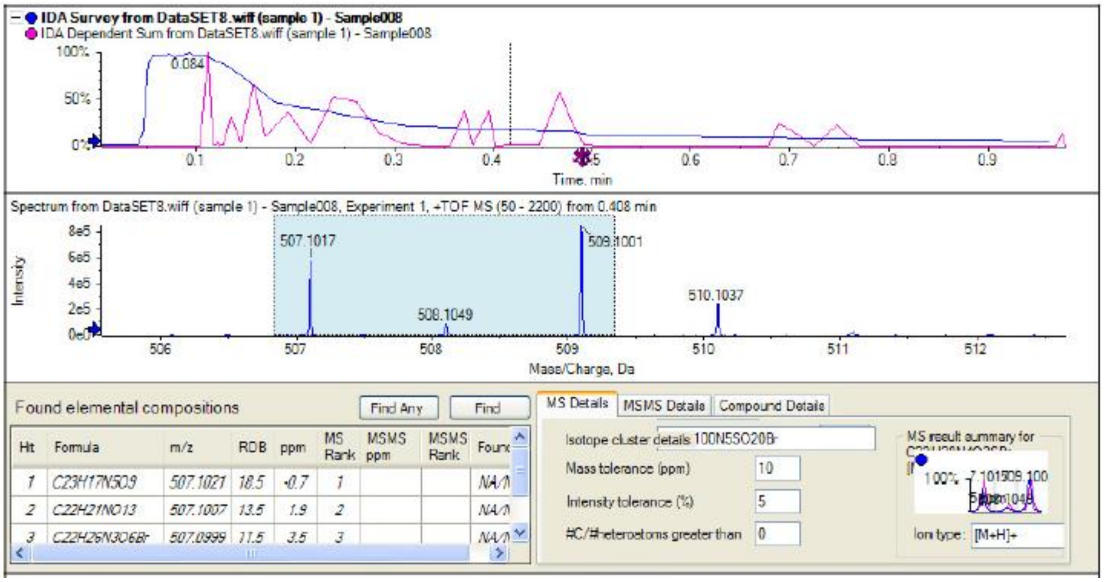

15j. ^{1}H NMR

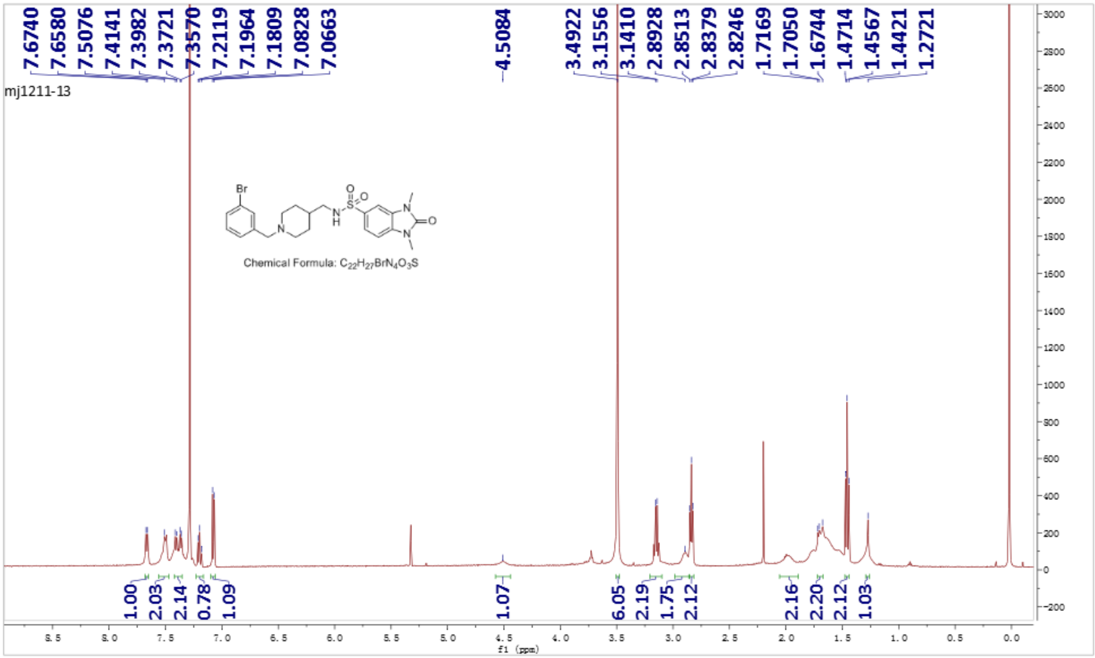

15j. ^{13}C NMR

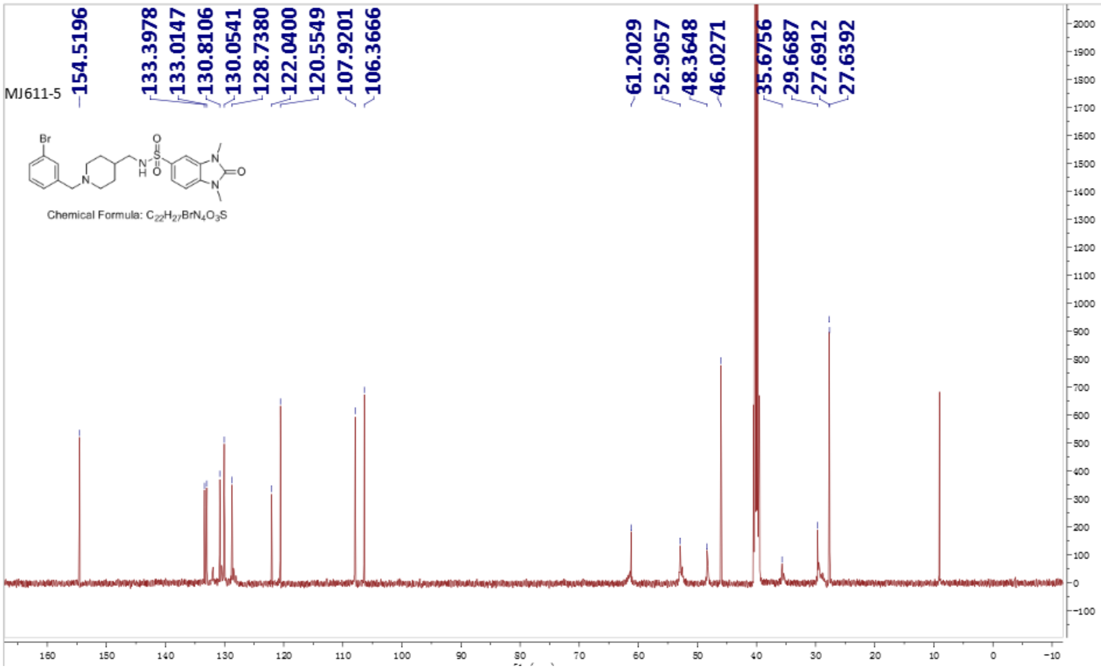

15j.HRMS (ESI)

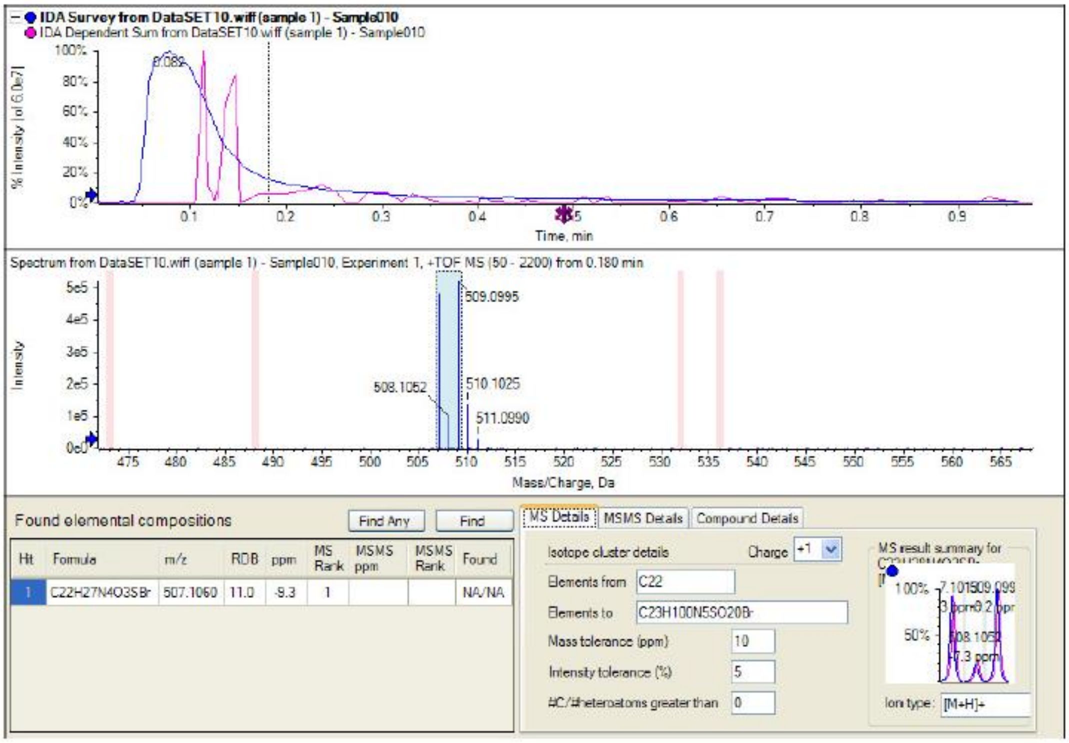

The ^{1}H NMR spectrum of Intermediate compounds

6 .^{1}H NMR

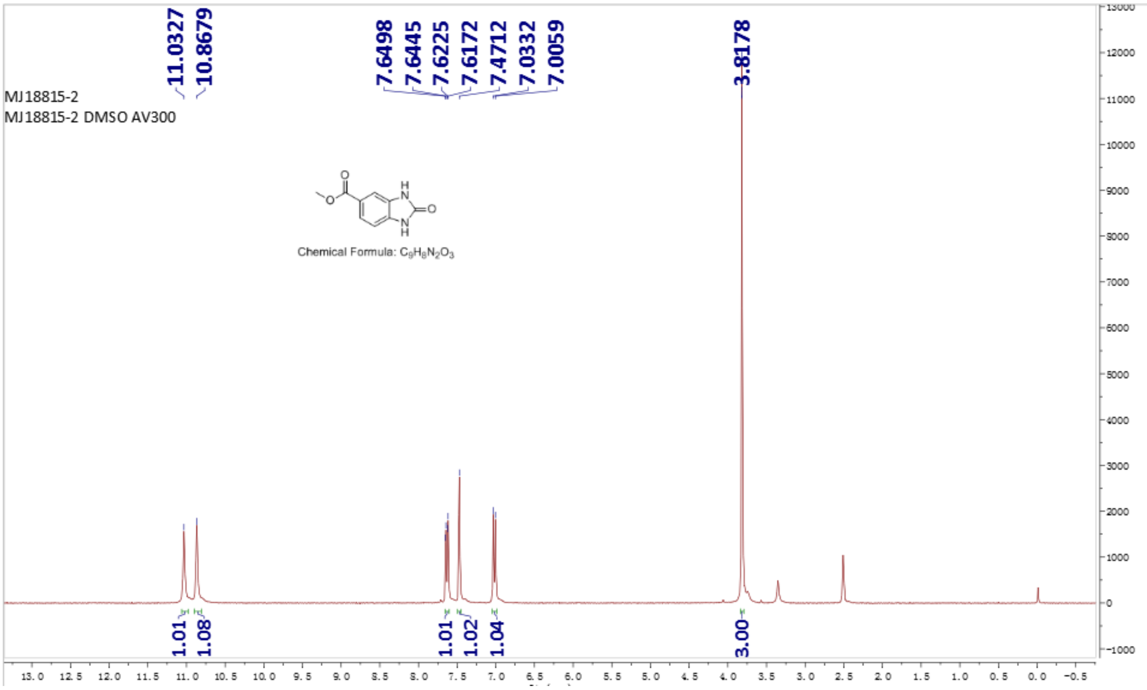

7. ^{1}H NMR

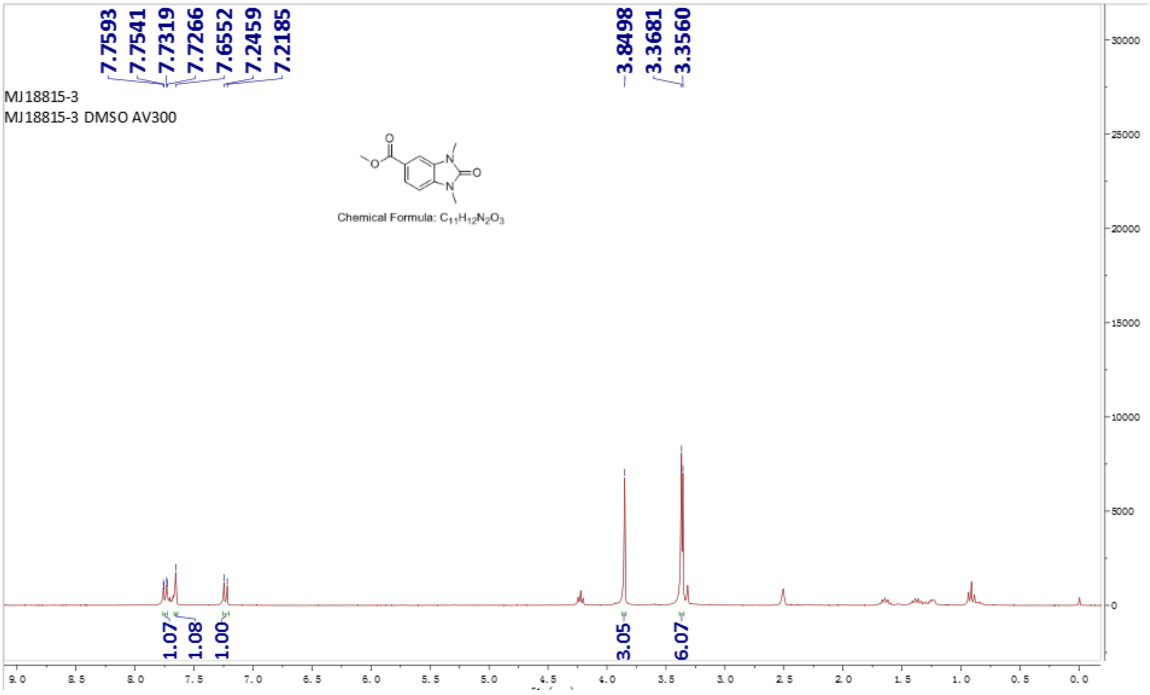

8. ^{1}H NMR

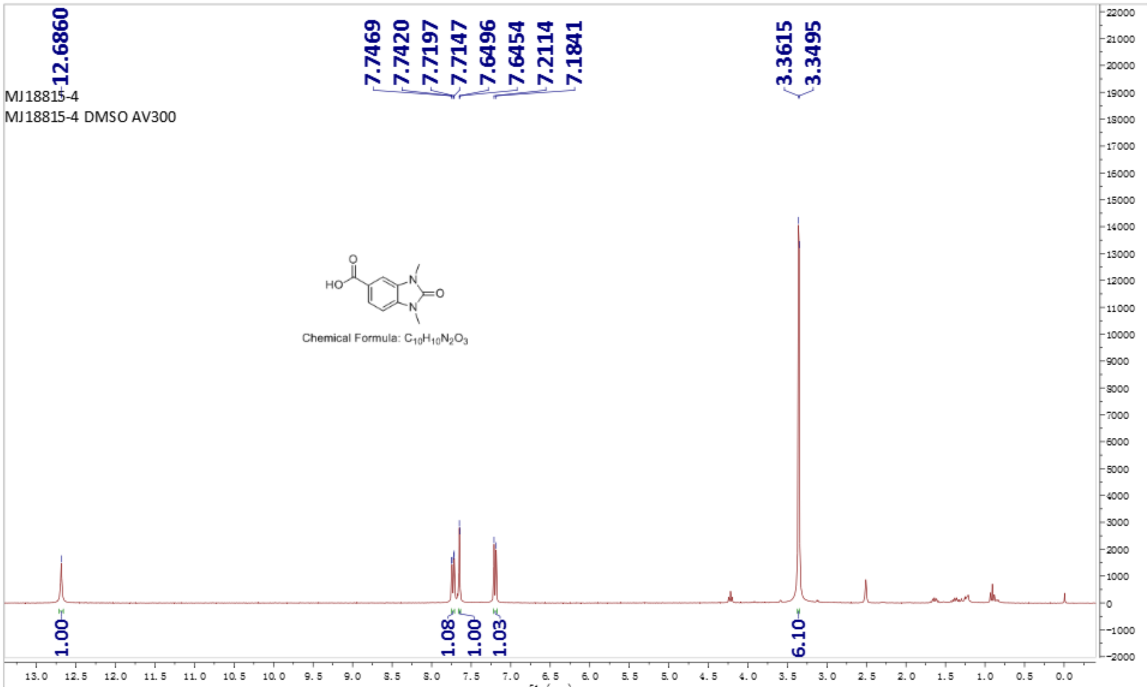

MJ18815-4
MJ18815-4 DMSO AV300
12.6860
7.7469
7.7420
7.7197
7.7147
7.6496
7.6454
7.2114
7.1841
3.3615
3.3495
HO
O
N
N
O
Chemical Formula: $C_{10}H_{10}N_2O_3$
1.00
1.08
1.00
1.03
6.10

References:

30. Ramunno A, Cosconati S, Sartini S, et al. Progresses in the pursuit of aldose reductase inhibitors: The structure-based lead optimization step. Eur J Med Chem 2012;51:216–26.

31. Cheung J, Rudolph MJ, Burshteyn F, et al. Structures of Human Acetylcholinesterase in Complex with Pharmacologically Important Ligands. J Med Chem 2012;55:10282–6.

32. Brus B, Kosak U, Turk S, et al. Discovery, Biological Evaluation, and Crystal Structure of a Novel Nanomolar Selective Butyrylcholinesterase Inhibitor. J Med Chem 2014;57:8167–79.
